# Systematic meta-analysis of the toxicities and side effects of the targeted drug lenvatinib

**DOI:** 10.1080/07853890.2025.2598935

**Published:** 2025-12-24

**Authors:** Huihui Liu, Kai Wang, Yu Sun, Qingwei Li, Jingfei Shi, Shuai Gao, Chao Cui

**Affiliations:** a Department of Hepatology, Qilu Hospital of Shandong University, Jinan, China; b Department of Infectious Disease, Qilu Hospital of Shandong University Dezhou Hospital, Dezhou, China; c School of Clinical Medicine, Shandong Second Medical University, Weifang, China; d Department of Clinical and Basic Medicine, Shandong First Medical University, Jinan, Shandong, China

**Keywords:** Lenvatinib, toxicities and side effects, cancer/tumor therapy, meta-analysis, any grade, grade ≥3

## Abstract

**Background:**

Lenvatinib, an effective targeted drug for various cancers, has clinical medication safety concerns due to its toxicities and side effects.

**Objective:**

This study evaluated lenvatinib-induced any adverse events (any AEs) and nine aspects: vascular toxicities related to the circulatory system (vascular toxicities, blood system, and heart), toxicities of the skin and its appendages (skin/subcutaneous tissue and taste system), toxicities of the respiratory system (respiratory, thoracic, and mediastinal and respiratory tract), toxicities of the nervous system (nervous system and general), toxicities of the digestive system (gastrointestinal and liver), toxicities of the urinary system, toxicities of the endocrine and metabolic system (endocrine and metabolism/nutrition), toxicities of the musculoskeletal system, and other severe toxicities. Toxicities and side effects were stratified by severity into any and ≥3 grades for analysis.

**Patients/Materials and Methods:**

Multiple databases were searched for lenvatinib cancer clinical studies (cohort studies and randomized controlled trials) from inception to December 31, 2024; toxicity and side effect data were extracted and analyzed.

**Results:**

Nine high-quality studies were included, showing that lenvatinib is effective in cancers but has notable toxicities. Taking hypertension as an example, for any grade, the risk ratio (RR) was 2.34 with a 95% confidence interval (CI) of [2.09, 2.62], a Z-value of 14.74, and a *P*-value <0.00001; for grade ≥3, the RR was 2.60 with a 95% CI of [2.21, 3.06], a Z-value of 11.44, and a *P*-value <0.00001.

**Conclusion:**

Lenvatinib is effective for cancer but toxic, and this study supports its rational clinical use.

## HIGHLIGHTS

**Key Messages:**

1. This systematic meta-analysis of lenvatinib (a targeted anticancer drug) clarifies its toxicities and side effects across various cancers, addressing the lack of integrated toxicity data for clinical decision-making.
2. By synthesizing data from 9 high-quality studies, the research confirms that lenvatinib’s therapeutic efficacy is accompanied by significant toxicities and side effects (e.g., elevated hypertension risk), providing actionable evidence for safe clinical use.

**Study Highlights:**

1. Examined the toxicities and side effects of the targeted drug lenvatinib in the treatment of various cancers.
2. Conducted a systematic review and meta-analysis to comprehensively evaluate the toxicities and side effects of lenvatinib across 16 aspects.
3. Included 9 high-quality studies, revealing that lenvatinib’s efficacy was accompanied by significant toxicities and side effects.
4. Taking hypertension as an example, lenvatinib increased the risk of toxicities and side effects of any grade, including grade ≥3.
5. The study provides a scientific basis for the rational clinical use of lenvatinib.

## 1. Introduction

In the development history of tumor treatment drugs, the emergence of targeted drugs has brought a revolutionary breakthrough to cancer treatment [1]. As a novel multi-target tyrosine kinase inhibitor, lenvatinib has attracted extensive attention in the global field of tumor treatment since its research and development. In 2007, Eisai Company took the lead in initiating relevant research on lenvatinib for cancer treatment [2]. In 2015, the U.S. FDA approved lenvatinib for the treatment of locally recurrent or metastatic, progressive radioactive iodine-refractory differentiated thyroid cancer, marking its official entry into the clinical application stage. In 2016, the FDA approved the combination of lenvatinib and everolimus for advanced renal cell carcinoma after failure of anti-angiogenic drugs. In 2018, lenvatinib was approved in China for the treatment of unresectable hepatocellular carcinoma that has not received prior systemic treatment, and in the same year, it was approved in the United States for first-line treatment of liver cancer [3]. Relying on its advantages in the research, development and patent layout of lenvatinib, Eisai Company has become the main developer and marketer of this drug, and through its global marketing network, it has promoted lenvatinib to the tumor treatment markets around the world.

Lenvatinib can selectively inhibit multiple targets, including vascular endothelial growth factor receptor 1–3 (VEGFR1–3), fibroblast growth factor receptor 1–4 (FGFR1–4), platelet-derived growth factor receptor α (PDGFRα), rearranged during transfection (RET), and K-Cell Receptor Tyrosine Kinase (KIT). By blocking tumor angiogenesis and inhibiting tumor cell proliferation and metastasis, it has demonstrated significant therapeutic effects in the treatment of various solid tumors, covering unresectable hepatocellular carcinoma, radioactive iodine-refractory differentiated thyroid cancer, urothelial cancer, metastatic renal cell carcinoma, non-small cell lung cancer, etc., bringing new therapeutic hopes to cancer patients worldwide [3–8]. However, with its widespread application, various toxicities and side effects caused by lenvatinib have gradually become prominent. These toxicities and side effects not only affect the quality of life of patients but also significantly interfere with the treatment outcomes.

Currently, there is a lack of systematic and comprehensive research on the comprehensive evaluation of the toxicities and side effects of lenvatinib [9–12]. In view of this, there is an urgent need for us to conduct a systematic review and meta-analysis, which aims to comprehensively integrate and make full use of the existing research resources, so as to objectively and impartially evaluate the toxicities and side effects of lenvatinib during the treatment process, especially the possible negative impacts on aspects such as the quality of life of patients and the functions of key organs.

## 2. Methods

### 2.1. Ethics approval statement

Ethical approval was not required for this study because it involved the retrieval and synthesis of data from previously published studies.

### 2.2. Literature search

We systematically searched multiple internationally renowned databases, including PubMed, Embase, and Cochrane Library, as well as Chinese databases such as China National Knowledge Infrastructure (CNKI) and Wanfang, covering a time range from the inception of each database until December 31, 2024 (Supplementary Table 1).

The specific search strategy included using exhaustive combinations of keywords such as ‘lenvatinib’, ‘toxicities and side effects’, and combining them with relevant study types (e.g. cohort studies, randomized controlled trials, long-term observational studies, etc.) to ensure comprehensive and accurate identification of all relevant research literature on the toxicities and side effects of lenvatinib.

**Figure 1.**
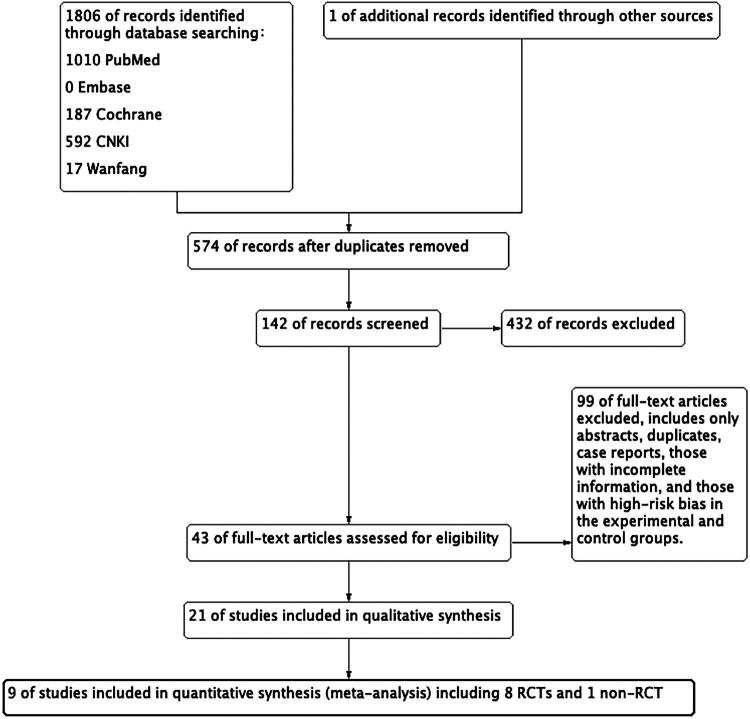
Study selection process. This figure illustrates the data selection process for conducting a systematic review and meta-analysis by searching multiple databases. A total of 1806 citations were identified across five databases. After applying the inclusion and exclusion criteria, 9 studies were ultimately selected and included in the meta-analysis.

Through this systematic literature search method, a total of 1,808 relevant studies were ultimately identified (including 1 additional study retrieved by manual reference checking). After screening based on titles, abstracts, and full texts, 9 high-quality studies were included, involving 6,441 patients (with 3,640 patients in the lenvatinib group). The distribution of citations across databases was as follows: 1,010 from PubMed, 0 from Embase, 187 from the Cochrane Library, 592 from CNKI, and 17 from Wanfang. The search and screening process is detailed in Figure 1, which provides a solid foundation for the subsequent systematic review and meta-analysis.

### 2.3. Inclusion criteria

In accordance with the pre-registered protocol of this study and following the Preferred Reporting Items for Systematic Reviews and Meta-Analyses (PRISMA) guidelines for conducting and reporting results, we included published studies that met the following criteria:

1. Studies must include patients treated with the targeted drug lenvatinib and focus on its toxicities and side effects.
2. Studies should provide detailed data on the toxicities and side effects of lenvatinib, including but not limited to the incidence rate, severity, and management of adverse events (AEs).
3. The study design should be cohort studies, RCTs, or long-term observational studies to ensure the reliability and validity of the data.
4. Studies should include a sufficient sample size to support the statistical significance of the conclusions drawn.

Among the 9 eligible studies finally included in this research, 8 were RCTs involving a total of 4,234 patients (2,297 patients in the lenvatinib group and 1,937 patients in the control group), and 1 was a non-RCT involving 2,207 patients (1,343 patients in the lenvatinib group and 864 patients in the control group). All studies excluded patients with complicated severe underlying diseases or those receiving other interfering treatments. The detailed distribution of patients is presented in Supplementary Table 2.

### 2.4. Exclusion criteria

Studies in the following situations will be excluded from this systematic review and meta-analysis (Supplementary Table 2):

1. Non-clinical studies, including animal experiments, in vitro studies, or cell culture experiments.
2. Studies that did not use the targeted drug lenvatinib for treatment or only involved short-term treatment.
3. Studies that did not provide detailed data on the toxicities and side effects of lenvatinib, such as the incidence rate, severity, and management of AEs.
4. Duplicate publications or studies based on the same dataset but lacking new analyses or conclusions (i.e., studies with repeated data and secondary analyses).
5. Studies with poor methodological quality and high risk of bias, such as those that did not follow standard research design, data collection, or analysis methods.
6. Studies published in non-English languages.
7. Studies from non-RCTs, including master’s and doctoral dissertations, books, study protocols, conference abstracts, case reports, and correspondences.

### 2.5. Data extraction

Data extraction was conducted by two independent reviewers. These two reviewers meticulously extracted key information from each included study, covering the basic characteristics of patients (e.g. age, gender, and duration of illness), the lenvatinib treatment regimen adopted (including dose, frequency, and duration), and specific data on toxicities and side effects. The latter encompassed the types of AEs, their incidence rates, and severity graded according to the Common Terminology Criteria for Adverse Events (CTCAE).

To ensure the accuracy and completeness of data extraction, each reviewer will use a standardized form pre-designed by the research team. This form lists in detail each data point that needs to be extracted, thereby assisting the reviewers in systematically collecting information.

During the data extraction process, if any uncertain or ambiguous information is encountered, the reviewers will attempt to clarify it by consulting the full text of the original study, contacting the study authors, or referring to other relevant literature. Especially when evaluating multiple studies from the same research team or the same study site, the reviewers will pay special attention to the potential overlap in inclusion criteria, recruitment periods, and types of interventions, in order to avoid duplicate counting or inclusion of the same patient data.

For multiple articles reporting on the same study population, our principle is to include only the most recent publication, unless different publications provide additional information that helps reduce the risk of bias. Any disagreements or questions that arise during the data extraction process will be resolved through discussion with other members of the research team or consultation with a senior expert serving as a third reviewer, to ensure the accuracy and reliability of the final data.

Two evaluators independently screened the literature, with the authors’ names, journal titles, years, and countries concealed. They extracted data and assessed the quality of the studies included. The core information extracted included the following: 9 categories of toxicities and side effects (graded as ‘any grade’ and ‘grade ≥3’ per the CTCAE, covering the circulatory system, skin and its appendages, respiratory system, etc.), patient grouping (lenvatinib group vs. other treatment groups), and basic characteristics such as study design, sample size, gender ratio, disease type, and treatment regimen. The detailed extracted results are summarized in Table 1, and Supplementary Table 3 lists basic data including abstracts of the latest published studies.

**Table 1.** Characteristics of studies included in the current meta-analysis.

Author (year)	Country [Asian, n (%)]	Study Design (RCTs/non-RCTs)	Median Age [years (rang)]	Sex (n/N, %)		Number of Patients (N)	Disease Type	Group (treatment group vs control group)	Dosage of medication (treatment group vs control group)
				Male	Female				
Casadei-Gardini et al. (2023)	1197/1343 (89.2%) vs 768/864 (88.9%)	non-RCTs	72 (65–79) vs 72 (64–78)	1058/1343 (78.8%) vs 682/864 (79.0%)	285/1343 (21.2 %) vs 182/864 (21.2%)	1343 vs 864	unresectable hepatocellular carcinoma	lenvatinib vs atezolizumab + bevacizumab	lenvatinib was administered (12 mg if baseline bodyweight was 60 kg or 8 mg if baseline body weight was <60 kg, given once daily orally) vs 1200 mg of atezolizumab plus 15 mg per kilogramme of body weight of bevacizumab intranously every 3 weeks
Haddad et al. (2017)	NR	RCTs	NR	125/261 (47.9%) vs 75/131 (57.3%)	136/261 (52.1%) vs 56/131(42.7%)	261 vs 131	differentiated cancer of the thyroid	lenvatinib vs placebo	lenvatinib (24 mg once daily) vs placebo in 28-day continuous cycles
Kiyota et al. (2017)	52/379 (13.7%) vs 32/204 (15.7%)	RCTs	63 (27–89) vs 62 (21–81)	186/379 (49.1%) vs 118/204 (57.8%)	193/379 (50.9%) vs 86/204 (42.2%)	379 vs 204	Radioiodine-Refractory Differentiated Thyroid Cancer	lenvatinib vs placebo	lenvatinib (24 mg/day; 28-day cycle) or placebo
Kudo et al. (2018)	334/478 (69.9%) vs 326/476 (68.5%)	RCTs	63.0 (20–88) vs 62.0 (22–88)	405/478 (84.7%) vs 401/476 (84.2%)	73/478 (15.3%) vs 75/476 (15.8%)	478 vs 476	unresectable hepatocellular carcinoma	lenvatinib vs sorafenib	oral lenvatinib (12 mg/day for bodyweight ≥60 kg or 8 mg/day for bodyweight <60 kg) vs sorafenib 400 mg twice-daily in 28-day cycles
Matsubara et al. (2024)	NR	RCTs	74 (66–79) vs 73 (67–78)	169/245 (69.0%) vs 184/242 (76.0%)	76/245 (31.0%) vs 58/242 (24.0%)	245 vs 242	Advanced Urothelial Carcinoma	lenvatinib plus pembrolizumab vs placebo plus pembrolizumab	lenvatinib 20 mg orally once daily plus pembrolizumab 200 mg intravenously every 3 wk vs placebo orally once daily plus pembrolizumab 200 mg intravenously every 3 wk
Motzer et al. (2015)	NR	RCTs	64 (41–79) vs 59 (37–77)	39/52 (75.0%) vs 38/50 (76.0%)	13/52 (25.0%) vs 12/50 (24.0%)	52 vs 50	metastatic renal cell carcinoma	lenvatinib vs everolimus	lenvatinib (24 mg/day) orally in continuous 28-day cycle vs everolimus (10 mg/day) orally in continuous 28-day cycle
Nair et al. (2021)	NR	RCTs	NR	405/478 (84.7%) vs 401/476 (84.2%)	73/478 (15.3%) vs 75/476 (15.8%)	478 vs 476	Unresectable Hepatocellular Carcinoma	lenvatinib vs sorafenib	lenvatinib (12 mg orally once daily for patients with a baseline body weight≥ 60 kg and 8 mg orally once daily for patients with a baseline body weight < 60 kg) vs sorafenib (400 mg orally twice daily)
Yang et al. (2024)	NR	RCTs	66 (34–85) vs 66 (37–87)	230/309 (74.4%) vs 224/314 (71.3%)	79/309 (25.6%) vs 90/314 (28.7%)	309 vs 314	First-Line Metastatic NSCLC With Programmed Cell Death-Ligand 1 Tumor Proportion Score of at least 1% (LEAP-007)	lenvatinib plus pembrolizumab vs placebo plus pembrolizumab	oral lenvatinib 20 mg vs placebo administered once daily
Zheng et al. (2021)	103/103 (100%) vs 48/48 (100%)	RCTs	61.0 (28–80) vs 60.0 (22–80)	57/103 (55.3%) vs 21/48 (43.8%)	46/103 (44.7%) vs 27/48 (56.3%)	103 vs 48	radioiodine-refractory differentiated thyroid cancer	lenvatinib vs placebo	lenvatinib 24 mg/day vs placebo in 28-day cycles

NR: Not Reported.

### 2.6. Risk of bias assessment and results

To ensure the accuracy and reliability of the study results, two independent researchers assessed the risk of bias for all included studies. For RCTs, the Cochrane Risk of Bias Tool was used to evaluate quality, specifically addressing six aspects: selection bias (random sequence generation and allocation concealment), performance bias (blinding of participants, personnel), detection bias (blinding of outcome assessment), attrition bias (incomplete outcome data), reporting bias (selective reporting), and other bias. For non-RCT studies, the Newcastle-Ottawa Scale was employed for quality evaluation, adopting a 22-point scoring system covering nine dimensions: representativeness of the exposed cohort (maximum 4 points), selection of the non-exposed cohort (maximum 2 points), ascertainment of exposure (maximum 3 points), demonstration that outcome of interest was not present at baseline (maximum 2 points), comparability of cohorts based on the design or analysis (maximum 2 points), assessment of outcomes (maximum 3 points), was follow-up long enough for outcomes to occur (maximum 1 point), and adequacy of follow-up of cohorts (maximum 1 point). The total score (ranging from 0 to 22 points) was used to characterize the risk of bias, with higher scores indicating lower risk of bias and higher study quality. We defined studies with a total score ≥16 as high-quality, 10–15 as moderate-quality, and ≤9 as low-quality.

The final assessment results showed that the risk of bias in RCTs was detailed in Supplementary Figure 1 and Supplementary Table 4, while the risk of bias in non-RCTs was detailed in Supplementary Table 5. These results provided an important basis for the accurate interpretation of the meta-analysis findings.

### 2.7. Data analysis and statistical methods

To comprehensively and thoroughly investigate the toxicities and side effects of the targeted drug lenvatinib, we will utilize professional statistical software to conduct systematic and detailed analysis of the collected data. The specific strategies will encompass the following key aspects:

Firstly, we will perform descriptive statistical analysis to intuitively reveal the basic characteristics and distribution patterns of the data, laying a foundation for subsequent in-depth analysis.

Subsequently, for indicators of lenvatinib’s toxicities and side effects, we will employ meta-analysis methods. In this process, we will use a random effects model to estimate risk ratio (RR) and their 95% CIs for the incidence and severity of various toxicities and side effects. This analysis will be based on data extracted from each full-text study after quality assessment, fully leveraging the characteristics of binomial or Poisson distributions. For continuous indicators of toxicities and side effects (such as changes in hypertension, diarrhea, palmar-plantar erythrodysesthesia syndrome, and liver function abnormalities), we will calculate the weighted average difference between the baseline value and the longest follow-up time for each study, thereby estimating the combined effect. To achieve this, we will use professional software such as STATA (version 17.0) and R Studio (version 1.3.1093) for targeted meta-analysis.

In assessing statistical heterogeneity, we will adopt the Cochran Q statistic and I^2^ statistic. Specifically, when the P-value is less than 0.1 and the I^2^ value is greater than or equal to 50%, we will consider that there is high heterogeneity among studies; whereas when the I^2^ value is less than 50%, we will consider that the heterogeneity among studies is within an acceptable range and select an appropriate model accordingly for combined effect estimation.

To further explore the sources of potential high heterogeneity and enhance the reliability of results, we will conduct both meta-regression analysis and subgroup analysis. For meta-regression analysis, we will incorporate pre-specified continuous and categorical covariates (including publication year, sample size, patient age, study region, and lenvatinib dosage) into the model to identify factors contributing to heterogeneity; a P-value < 0.05 will be considered indicative of a significant heterogeneity source. For subgroup analysis, we will stratify the data by key clinical and methodological factors (such as disease type, treatment duration, and risk of bias level) to compare effect sizes across subgroups, and calculate the P value for interaction to assess whether the intervention effect differs significantly between subgroups. The combination of these two methods enables targeted identification of heterogeneity sources and accurate interpretation of pooled results.

Furthermore, to comprehensively include all relevant data, regardless of the chosen effect measure, we will use continuity correction methods (such as adding 0.5) to handle trials with zero events, ensuring a balanced sample size between the two groups and thereby enhancing the accuracy and reliability of the meta-analysis.

Finally, to evaluate potential publication bias, we will utilize funnel plot testing methods combined with rigorous verification using Egger’s test or Begg’s test. The funnel plot results showed that the scattered points were evenly and symmetrically distributed (Supplementary Figure s 2–11), indicating no significant publication bias in the included studies, which ensures the reliability of the meta-analysis results.

**Figure 2.**
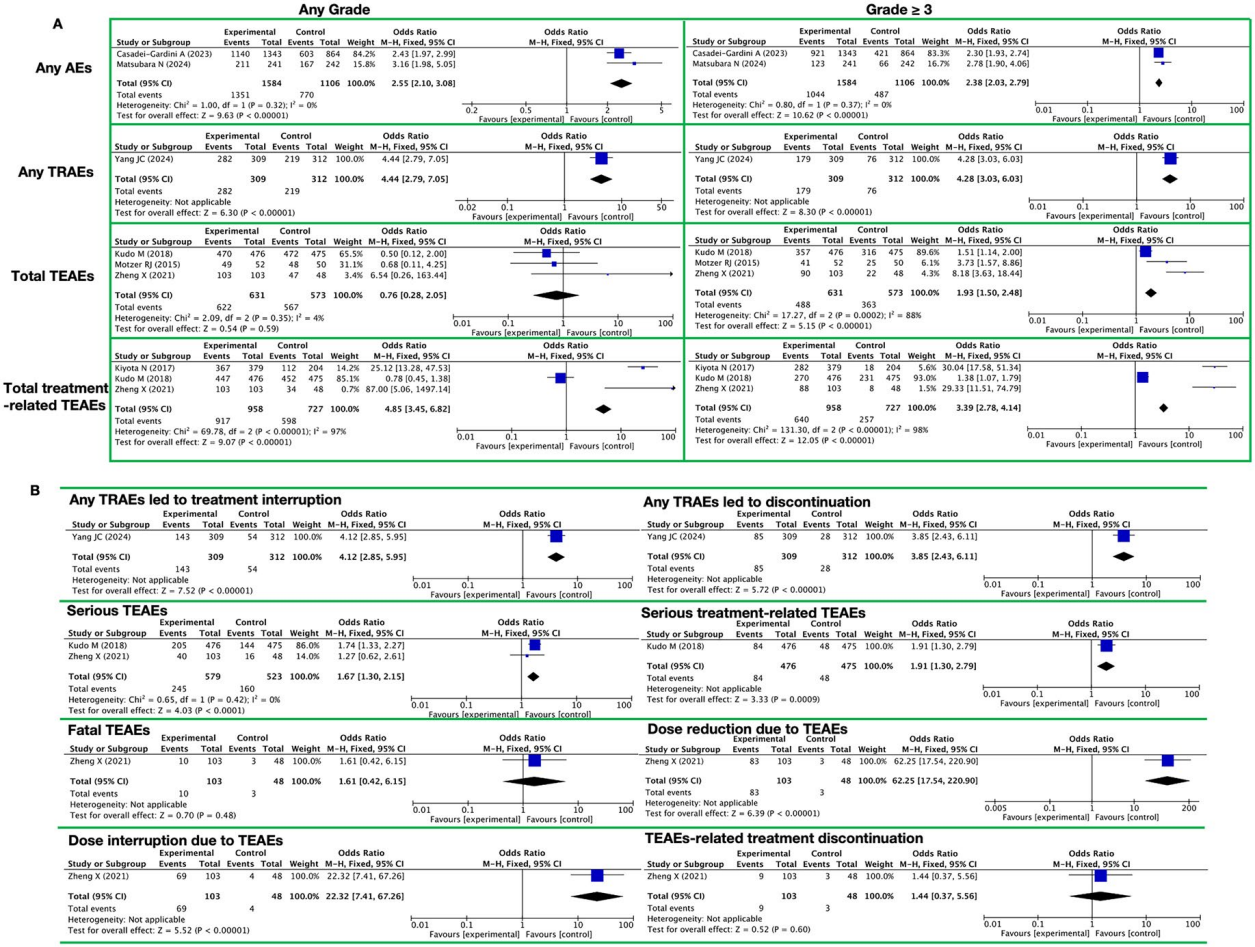
Meta-analysis of lenvatinib for any AEs. A. Meta-analysis of any AEs, any TRAEs, total TEAEs and total treatment-related TEAEs B. Meta-analysis of lenvatinib for other any AEs (any grade) in patients

**Figure 3.**
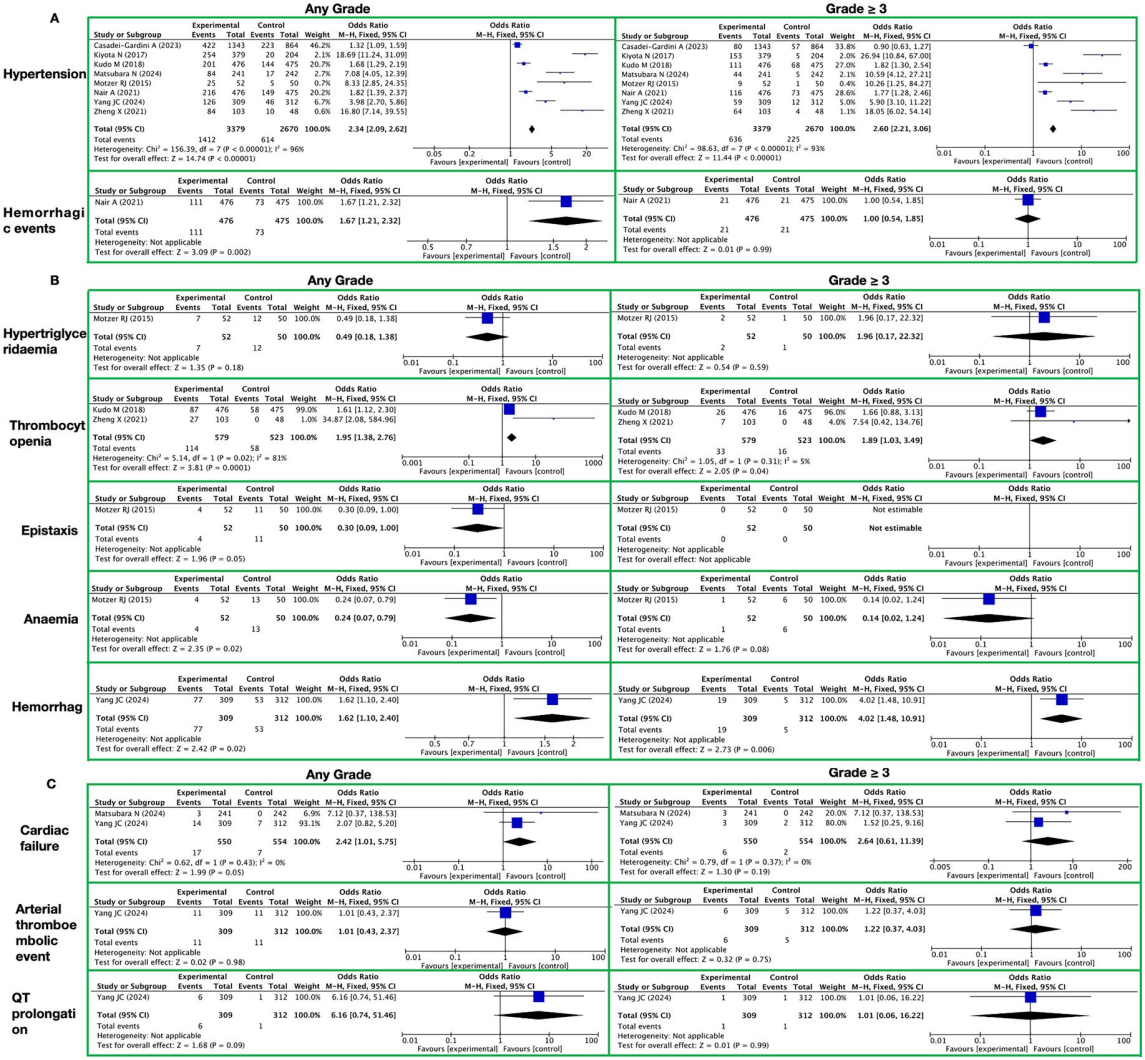
Meta-analysis of the circulatory system-related toxicity of lenvatinib. A. Vascular Toxicities It presents vascular toxicities, with toxicity grades (any grade, grade ≥ 3) on the horizontal axis and specific toxicity types (hypertension, hemorrhagic events) on the vertical axis. B. Blood System It presents blood system-related toxic reactions, with toxicity grades (any grade, grade ≥ 3) on the horizontal axis and specific toxicity types (hypertriglyceridaemia, thrombocytopenia, epistaxis, anaemia, hemorrhage) on the vertical axis. C. Heart It presents heart-related toxic reactions, with toxicity grades (any grade, grade ≥ 3) on the horizontal axis and specific toxicity types (cardiac failure, arterial thromboembolic event, QT prolongation) on the vertical axis.

**Figure 4.**
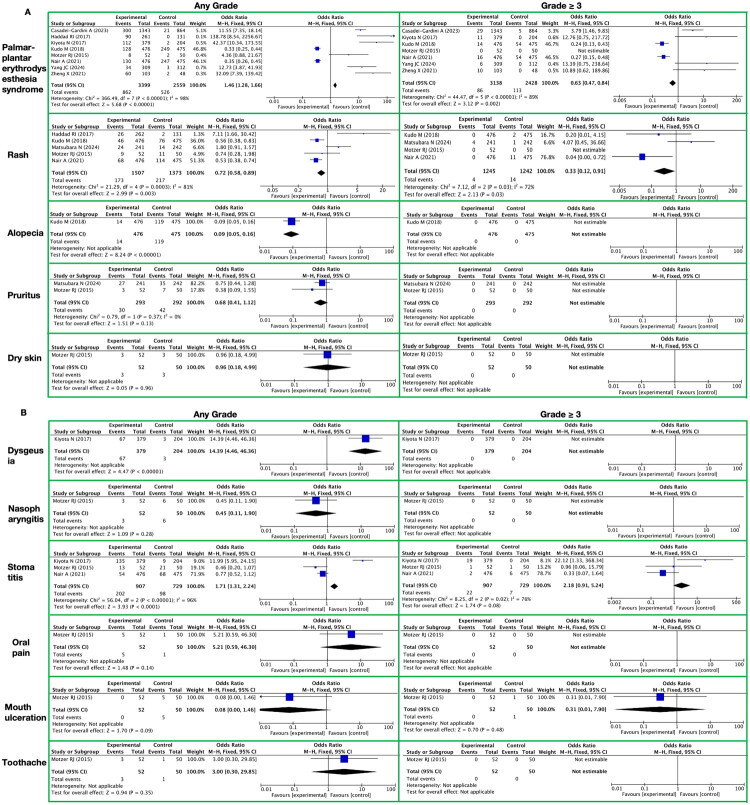
Meta-analysis of the toxicity of lenvatinib on the skin and sppendages. A. Skin/Subcutaneous Tissue It presents skin/subcutaneous tissue-related toxic reactions, with toxicity grades (any grade, grade ≥ 3) on the horizontal axis and specific toxicity types (palmar-plantar erythrodysesthesia syndrome, rash, alopecia, pruritus, dry skin) on the vertical axis. B. Taste System It presents taste system-related toxic reactions, with toxicity grades (any grade, grade ≥ 3) on the horizontal axis and specific toxicity types (dysgeusia, nasopharyngitis, stomatitis, oral pain, mouth ulceration, toothache) on the vertical axis.

**Figure 5.**
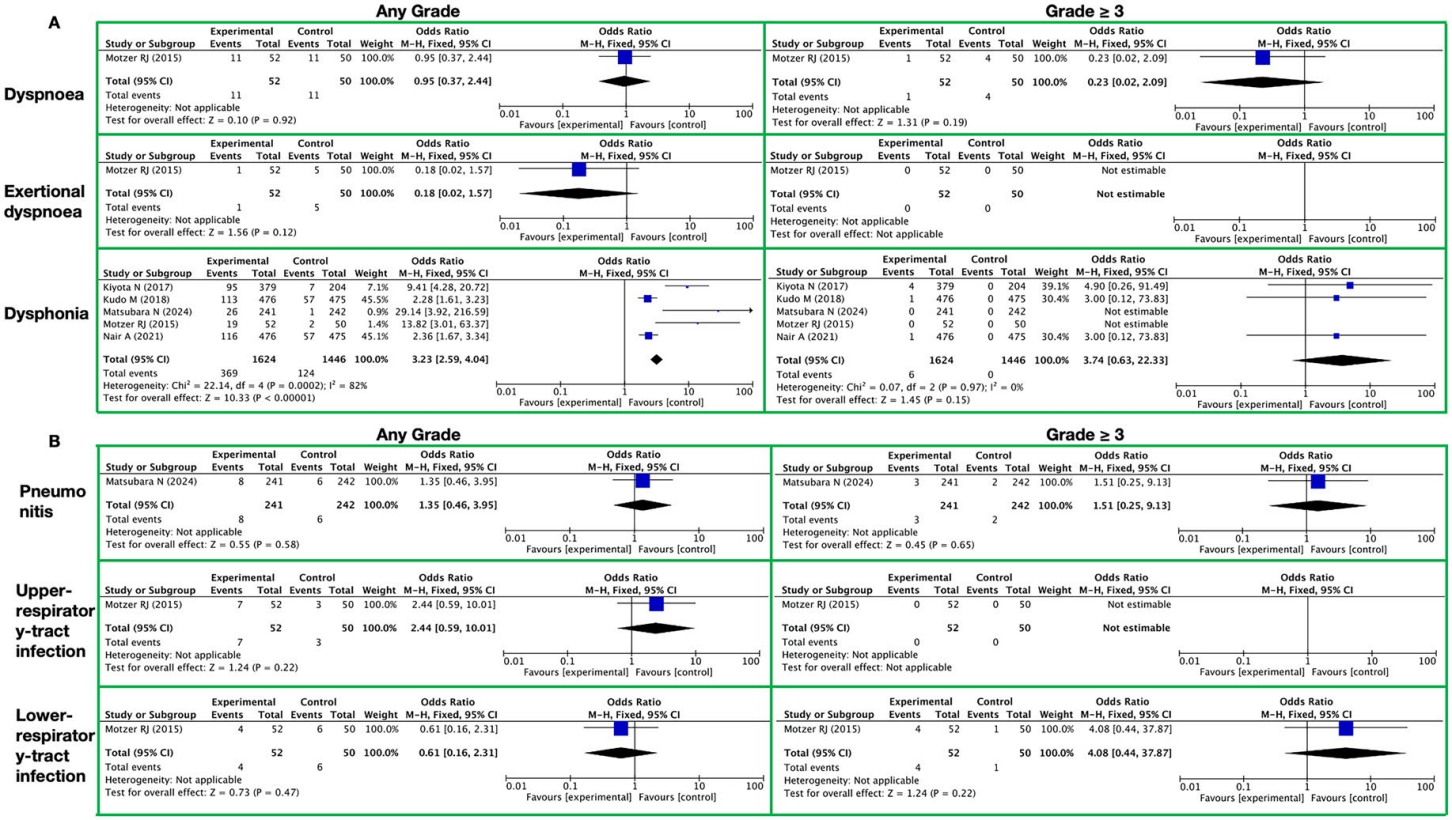
Meta-analysis of the respiratory system toxicity of lenvatinib. A. Respiratory, Thoracic, and Mediastinal It presents respiratory, thoracic, and mediastinal-related toxic reactions, with toxicity grades (any grade, grade ≥ 3) on the horizontal axis and specific toxicity types (dyspnoea, exertional dyspnoea, dysphonia) on the vertical axis. B. Respiratory Tract It presents respiratory tract-related toxic reactions, with toxicity grades (any grade, grade ≥ 3) on the horizontal axis and specific toxicity types (pneumonitis, upper-respiratory-tract infection, lower-respiratory-tract infection) on the vertical axis.

**Figure 6.**
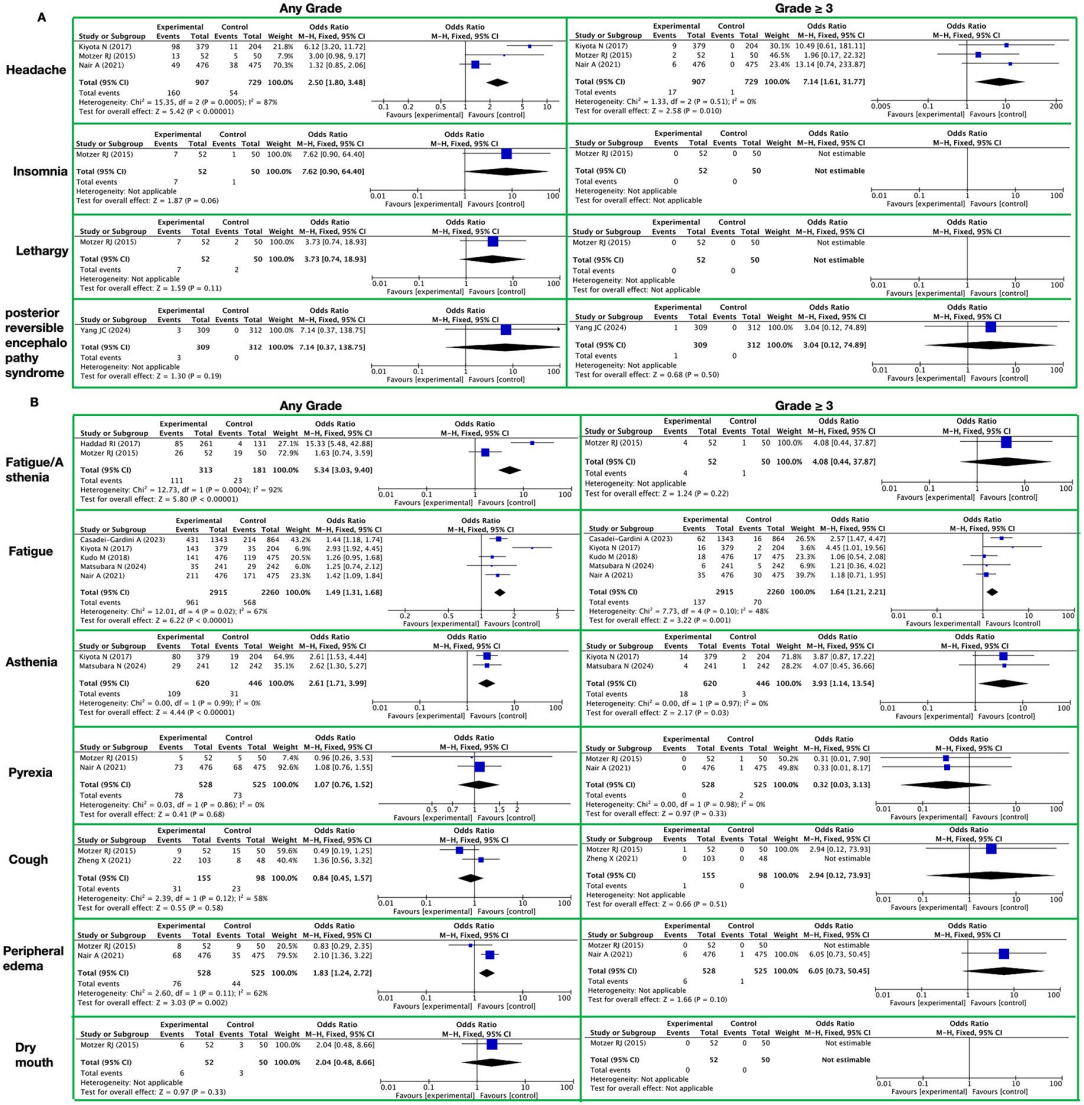
Meta-analysis of the toxicity of lenvatinib to the nervous system. A. Nervous System It presents nervous system-related toxic reactions, with toxicity grades (any grade, grade ≥ 3) on the horizontal axis and specific toxicity types (headache, insomnia, lethargy, posterior reversible encephalopathy syndrome) on the vertical axis. B. General It presents general toxic reactions, with toxicity grades (any grade, grade ≥ 3) on the horizontal axis and specific toxicity types (fatigue/asthenia, fatigue, asthenia, pyrexia, cough, peripheral edema, dry mouth) on the vertical axis.

**Figure 7.**
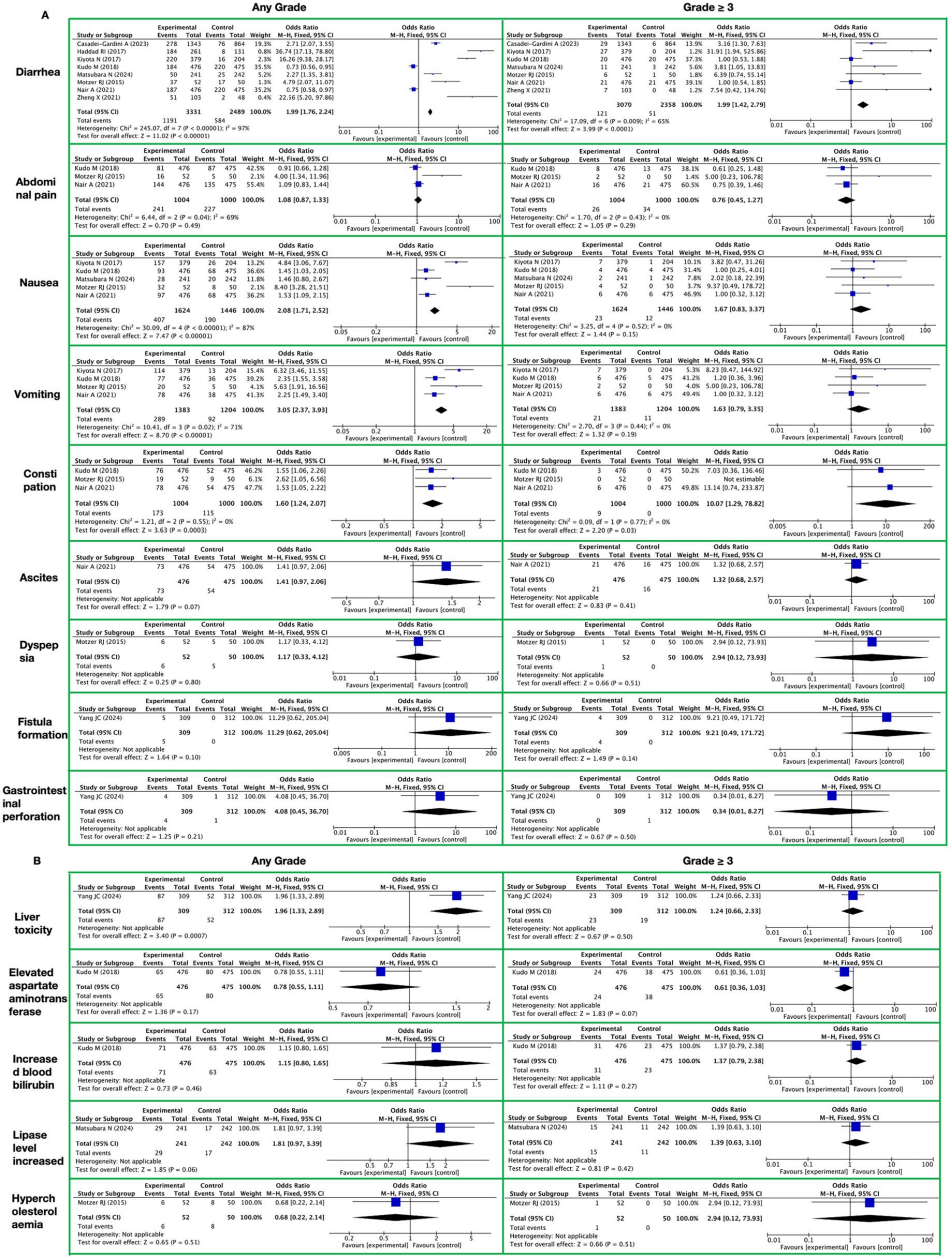
Meta-analysis of the toxicity of lenvatinib to the digestive system. A. Gastrointestinal It presents gastrointestinal-related toxic reactions, with toxicity grades (any grade, grade ≥ 3) on the horizontal axis and specific toxicity types (diarrhea, abdominal pain, nausea, vomiting, constipation, ascites, dyspepsia, fistula formation, gastrointestinal perforation) on the vertical axis. B. Liver It presents liver-related toxic reactions, with toxicity grades (any grade, grade ≥ 3) on the horizontal axis and specific toxicity types (liver toxicity, elevated aspartate aminotransferase, increased blood bilirubin, lipase level increased, hypercholesterolaemia) on the vertical axis.

**Figure 8.**
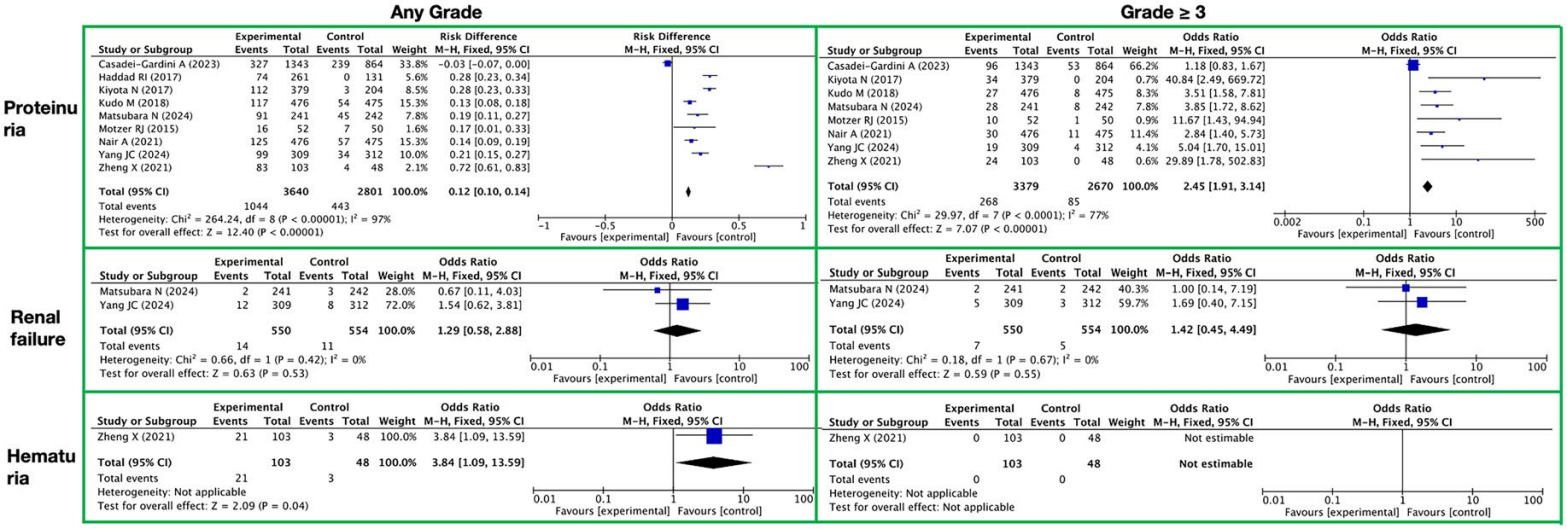
Meta-analysis of the toxicity of lenvatinib to the urinary system. It presents urinary system-related toxic reactions, with toxicity grades (any grade, grade ≥ 3) on the horizontal axis and specific toxicity types (proteinuria, renal failure, hematuria) on the vertical axis.

**Figure 9.**
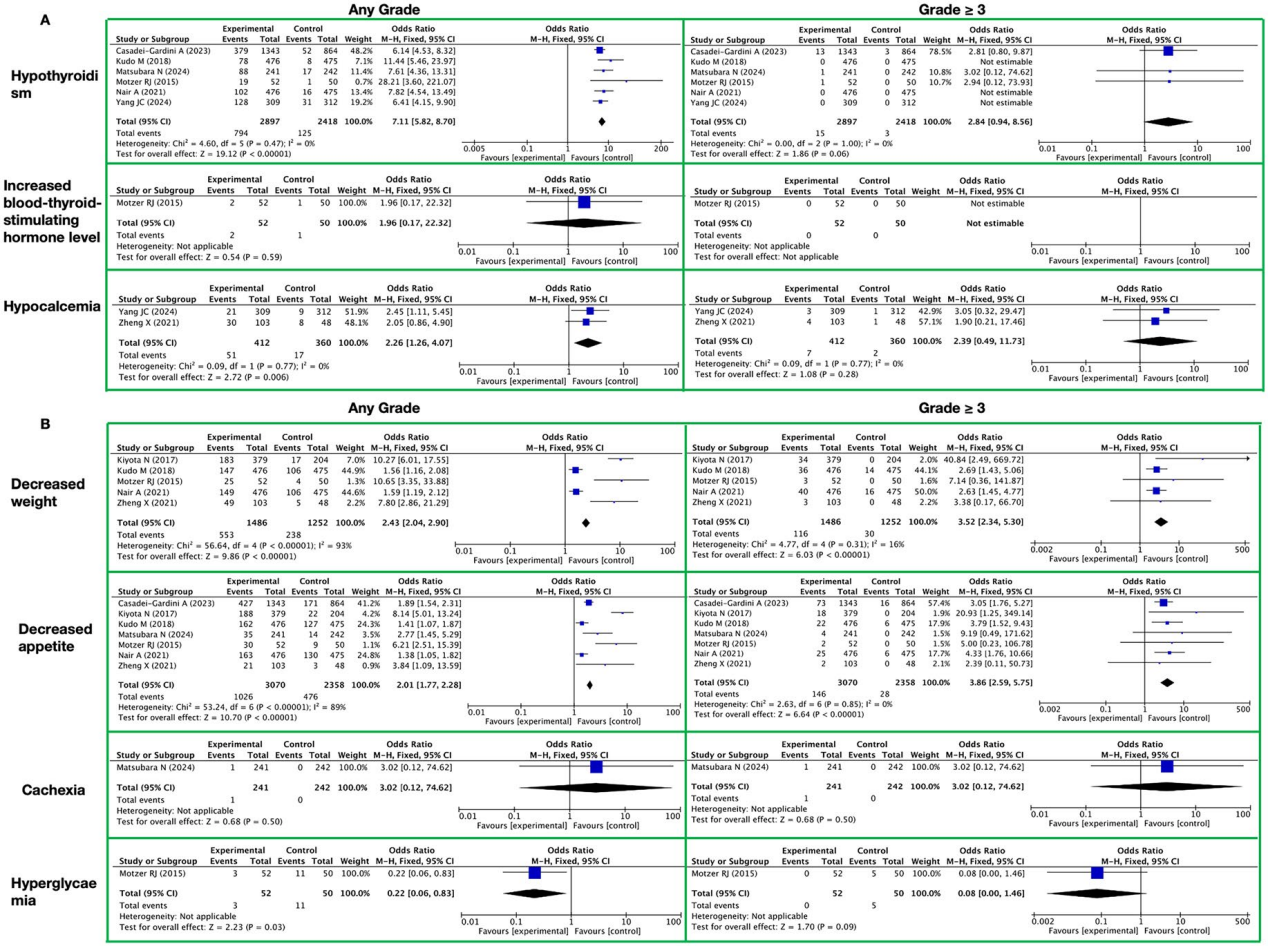
Meta-analysis of the endocrine and metabolic toxicity of lenvatinib. A. Endocrine It presents endocrine-related toxic reactions, with toxicity grades (any grade, grade ≥ 3) on the horizontal axis and specific toxicity types (hypothyroidism, increased blood-thyroid-stimulating hormone level, hypocalcemia) on the vertical axis. B. Metabolism/Nutrition It presents metabolism/nutrition-related toxic reactions, with toxicity grades (any grade, grade ≥ 3) on the horizontal axis and specific toxicity types (decreased weight, decreased appetite, cachexia, hyperglycaemia) on the vertical axis.

**Figure 10.**
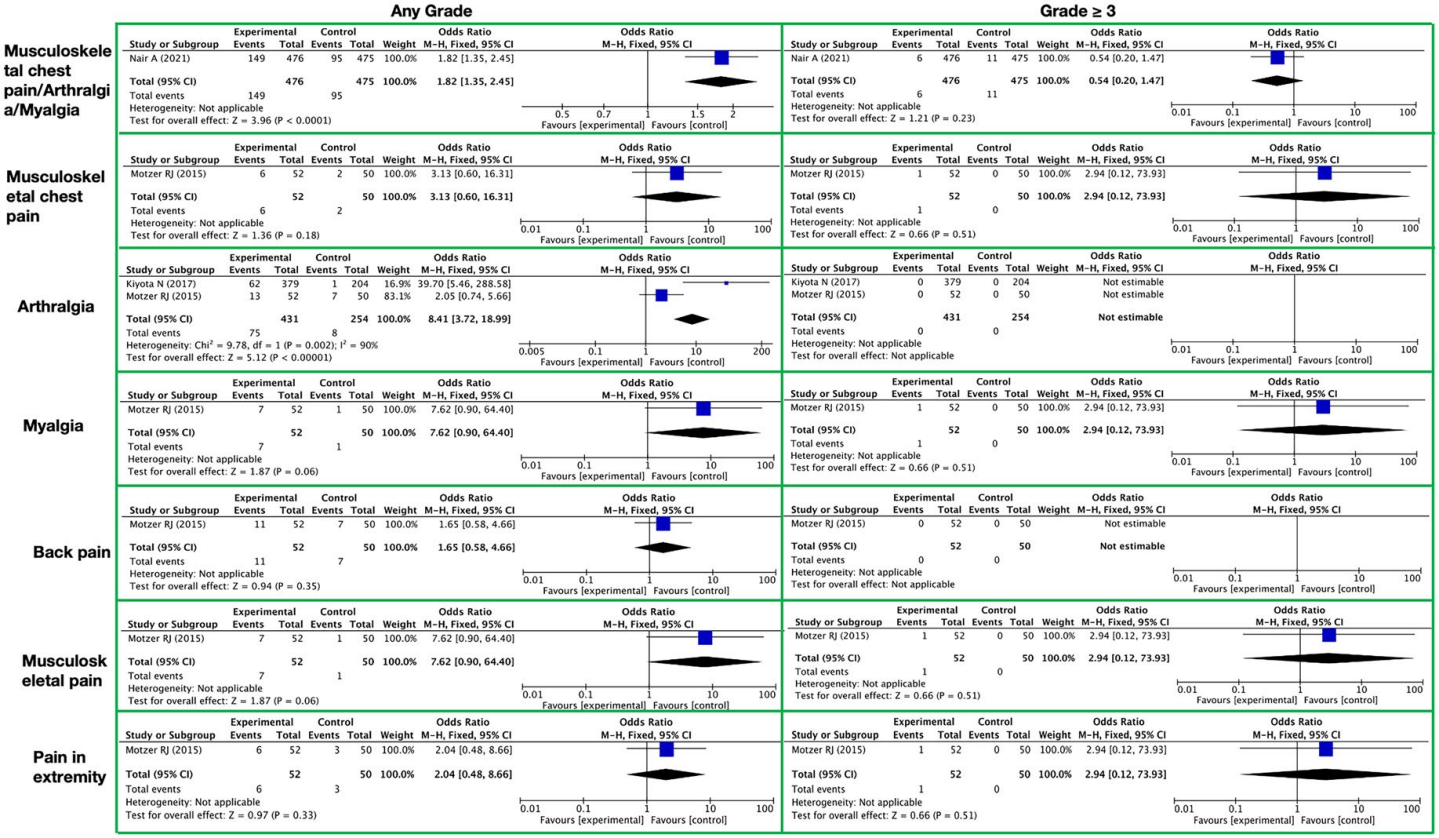
Meta-analysis of the toxicity of lenvatinib to the musculoskeletal system. It presents musculoskeletal system-related toxic reactions, with toxicity grades (any grade, grade ≥ 3) on the horizontal axis and specific toxicity types (musculoskeletal chest pain/arthralgia/myalgia, musculoskeletal chest pain, arthralgia, myalgia, back pain, musculoskeletal pain, pain in extremity) on the vertical axis.

**Figure 11.**
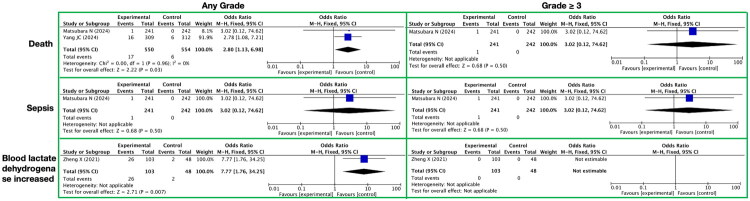
Meta-analysis of other severe toxicities of lenvatinib. It presents other severe toxicities, with toxicity grades (any grade, grade ≥ 3) on the horizontal axis and specific toxicity types (death, sepsis, blood lactate dehydrogenase increased) on the vertical axis.

## 3. Results

Through a rigorous literature search and screening process, 9 high-quality studies were included, involving 6,441 patients (with 3,640 patients in the lenvatinib group). Combined with systematic risk of bias assessment and data analysis, the scientific validity and reliability of the results were ensured, which provides key evidence for the clinical evaluation of lenvatinib-induced toxicities and side effects (Figure 1).

### 3.1. Characteristics of included studies

Among the 9 included studies, 8 adopted a RCTs design. These studies involved a total of 4,234 patients, of which 2,297 patients received lenvatinib treatment as the experimental group, while the remaining 1,937 patients received other treatment regimens or served as the control group. To ensure the accuracy and reliability of the study results, all included studies excluded patients with other concurrent severe diseases or those receiving other treatments that might affect the study outcomes. One study adopted a non-RCT design (real-world observational study), in which 1,343 patients received lenvatinib treatment and 864 patients received other treatment regimens.

In terms of treatment methods, the included studies mainly focused on the therapeutic effects and toxicities and side effects of lenvatinib under specific diseases or conditions. These studies generally covered multiple treatment cycles and carried out long-term follow-ups on patients to comprehensively evaluate the abnormal indicators of any AEs of lenvatinib and its nine aspects, including vascular toxicities related to the circulatory system (vascular toxicities, blood system, and heart), toxicities of the skin and its appendages (skin/subcutaneous tissue and taste system), toxicities of the respiratory system (respiratory, thoracic, and mediastinal and respiratory tract), toxicities of the nervous system (nervous system and general), toxicities of the digestive system (gastrointestinal and liver), toxicities of the urinary system, toxicities of the endocrine and metabolic system (endocrine and metabolism/nutrition), toxicities of the musculoskeletal system, and other severe toxicities. The evaluation of toxicities and side effects was conducted by dividing them into two grades: any grade and grade ≥3.

Differences in patients’ baseline characteristics (e.g. age, gender, disease type) and treatment regimens (lenvatinib dosage, frequency) were controlled through subgroup analysis (lenvatinib treatment group vs. other treatment groups). The key baseline characteristics are summarized in Table 1, which provides contextual support for the stratified interpretation of subsequent toxicities and side effect analyses.

### 3.2. Analysis of any AEs

In the systematic meta-analysis of the toxicities and side effects of the targeted drug lenvatinib, we comprehensively evaluated the AEs (Figure 2 and Supplementary Table 6), and the main results are as follows:

Firstly, regarding any AEs (Figure 2A), lenvatinib significantly increased the risk of AEs at any grade (RR = 2.55, 95% confidence interval [CI] [2.10, 3.08], *p* < 0.00001). The risk was also significant for AEs of grade ≥3 (RR = 2.38, 95% CI [2.03, 2.79], *p* < 0.00001).

Secondly, we analyzed the treatment-related adverse events (TRAEs), Total treatment-emergent adverse events (TEAEs), and Total treatment-related TEAEs (Figure 2A):

- For any TRAEs, the RRs at any grade and grade ≥3 were 4.44 (95% CI [2.79, 7.05], P < 0.00001) and 4.28 (95% CI [3.03, 6.03], P < 0.00001), respectively.
- For total TEAEs, the effect at any grade was not significant (RR = 0.76, P = 0.59), but the RR increased to 1.93 for grade ≥3 (95% CI [1.50, 2.48], P < 0.00001).
- For total treatment-related TEAEs, the RRs at any grade and grade ≥3 were 4.85 and 3.39, respectively (both P < 0.00001).

In addition, lenvatinib significantly increased the risks of various toxicities and side events (Figure 2B): treatment interruption due to TRAEs (RR = 4.12, *p* < 0.00001) and treatment discontinuation (RR = 3.85, *p* < 0.00001); Serious TEAEs (RR = 1.67, *p* < 0.0001) and Serious treatment-related TEAEs (RR = 1.91, *p* = 0.009); dose reduction due to TEAEs (RR = 62.25, *p* < 0.00001) and dose interruption (RR = 22.32, *p* < 0.00001). However, it had no significant effect on Fatal TEAEs (RR = 1.61, *p* = 0.48), and it did not significantly increase the risk of treatment discontinuation due to TEAEs (RR = 1.44, *p* = 0.60).

### 3.3. Analysis of toxicities and side effects

#### 3.3.1. Toxicity related to the circulatory system

##### 3.3.1.1. Vascular

In the systematic meta-analysis of the toxicities and side effects of the targeted drug lenvatinib, we particularly focused on its impact on the patients’ vascular system, and mainly evaluated the incidences of hypertension and hemorrhagic events (see Figure 3A and Supplementary Table 7).

Regarding hypertension, the study showed that in toxicities and side effects of any grade, the incidence of hypertension in the lenvatinib treatment group was significantly higher than that in the control group, with a RR of 2.34 (95% CI [2.09, 2.62]; *Z* = 14.74; *p* < 0.00001). In toxicities and side effects of grade 3 and above, the incidence of hypertension in the lenvatinib treatment group was still significantly higher than that in the control group, with an RR of 2.60 (95% CI [2.21, 3.06]; *Z* = 11.44; *p* < 0.00001). This indicates that the use of lenvatinib is significantly associated with an increased incidence of hypertension in patients.

Regarding hemorrhagic events, the study showed that in toxicities and side effects of any grade, the incidence of hemorrhagic events in the lenvatinib treatment group was significantly higher than that in the control group, with an RR of 1.67 (95% CI [1.21, 2.32]; *Z* = 3.09; *p* = 0.002). However, in toxicities and side effects of grade 3 and above, there was no significant difference in the incidence of hemorrhagic events between the lenvatinib treatment group and the control group, with an RR of 1.00 (95% CI [0.54, 1.85]; *Z* = 0.01; *p* = 0.99). This suggests that the use of lenvatinib may increase the incidence of hemorrhagic events in patients, but has no significant impact on severe hemorrhagic events.

##### 3.3.1.2. Blood system

During long-term treatment with lenvatinib, the impact on the blood system is mainly manifested in five aspects: hypertriglyceridemia, thrombocytopenia, epistaxis, anemia, and hemorrhage (Figure 3B and Supplementary Table 7).

For any grade of toxicities and side effects, the results showed that the incidence of hypertriglyceridemia was relatively low, with an RR of 0.49, 95% CI [0.18, 1.38], Z-value of 1.35, and P-value of 0.18, indicating no significant increase in risk. The incidence of thrombocytopenia was higher, with an RR of 1.95, 95% CI [1.38, 2.76], Z-value of 3.81, and P-value less than 0.0001, showing a significant increase in risk. For epistaxis, the RR was 0.30, 95% CI [0.09, 1.00], Z-value of 1.96, and P-value of 0.05, which, although close to significance, indicated an overall decreased risk. The RR for anemia was 0.24, 95% CI [0.07, 0.79], Z-value of 2.35, and P-value of 0.02, indicating a significant decrease in risk. The RR for hemorrhage was 1.62, 95% CI [1.10, 2.40], Z-value of 2.42, and P-value of 0.02, showing an increased risk.

For grade ≥ 3 toxicities and side effects, the results showed that the RR for hypertriglyceridemia was 1.96, but with a very wide 95% CI of [0.17, 22.32], a Z-value of 0.54, and a P-value of 0.59, indicating substantial uncertainty regarding the risk of occurrence at this grade). The RR for thrombocytopenia was 1.89, with a 95% CI of [1.03, 3.49], a Z-value of 2.05, and a P-value of 0.04, showing an increased risk of occurrence at this grade. No cases of epistaxis were observed at grade 3 or above. The RR for anemia was 0.14, with a 95% CI of [0.02, 1.24], a Z-value of 1.76, and a P-value of 0.08, which, although close to significance, indicated an overall decreased risk of occurrence at this grade. The RR for hemorrhage was 4.02, with a 95% CI of [1.48, 10.91], a Z-value of 2.73, and a P-value of 0.006, indicating a significant increase in the risk of occurrence at this grade.

##### 3.3.1.3. Heart

In the systematic meta-analysis focusing on the toxicities and side effects of the targeted drug lenvatinib, we conducted an in-depth exploration of the cardiac system-related toxicities and side effects, primarily including cardiac failure, arterial thromboembolic events, and QT prolongation (Figure 3C and Supplementary Table 7).

In terms of toxicities at any grade, the incidence of heart failure was significantly elevated with a RR of 2.42 (95% CI: [1.01, 5.75]; Z-value: 1.99; P-value approaching significance at 0.05) in patients treated with lenvatinib. This result suggests that lenvatinib therapy may increase the risk of heart failure. However, for arterial thromboembolic events and QT prolongation, the RRs associated with lenvatinib therapy did not demonstrate statistically significant differences. Specifically, the RR for arterial thromboembolic events was 1.01 (95% CI: [0.43, 2.37]; P-value: 0.98). For QT prolongation, although the RR increased to 6.16, the 95% CI was extremely wide ([0.74, 51.46]; P-value: 0.09), suggesting that the strength of this association may be influenced by other factors.

When considering toxicities of grade 3 or higher (grade ≥3), the impact of lenvatinib therapy on heart failure, arterial thromboembolic events, and QT prolongation did not reach statistical significance. Specifically, the RR for heart failure was 2.64 (95% CI: [0.61, 11.39]; P-value: 0.19); for arterial thromboembolic events, the RR was 1.22 (95% CI: [0.37, 4.03]; P-value: 0.75); and for QT interval prolongation, the RR was close to 1, at 1.01, with an extremely wide 95% CI of [0.06, 16.22] and a P-value of 0.99. These results indicate that, among toxicities of grade 3 or higher, lenvatinib therapy has a limited impact on cardiac-related toxicities.

#### 3.3.2. Toxicity of the skin and its appendages

##### 3.3.2.1. Skin/subcutaneous tissue

In the systematic meta-analysis of the toxicities and side effects of the targeted drug lenvatinib, the results concerning skin/subcutaneous tissue toxicities and side effects indicated that the use of lenvatinib is associated with a higher incidence rate of skin/subcutaneous tissue toxicities and side effects, primarily manifested in palmar-plantar erythrodysesthesia syndrome and rash (Figure 4A and Supplementary Table 8).

Specifically, in terms of any grade of toxicities and side effects, the incidence rate of palmar-plantar erythrodysesthesia syndrome significantly increased, with a RR of 1.46 (95% CI [1.28, 1.66]; *Z* = 5.68; *p* < 0.00001). Meanwhile, the incidence rate of rash relatively decreased, with an RR of 0.72 (95% CI [0.58, 0.89]; *Z* = 2.99; *p* = 0.003) However, for toxicities and side effects of grade 3 or higher, the incidence rate of palmar-plantar erythrodysesthesia syndrome decreased, with an RR of 0.63 (95% CI [0.47, 0.84]; *Z* = 3.12; *p* = 0.002), and the incidence rate of rash decreased even more significantly, with an RR of 0.33 (95% CI [0.12, 0.91]; *Z* = 2.13; *p* = 0.03).

Additionally, the study also evaluated other toxicities and side effects, including alopecia, pruritus, and dry skin. The results showed that, in any grade of toxicities and side effects, the incidence rate of alopecia was extremely low, with a RR of 0.09 (95% CI [0.05, 0.16]; *Z* = 8.24; *p* < 0.00001)). However, no significant differences were observed in the incidence rates of pruritus and dry skin, with RRs of 0.68 (95% CI [0.41, 1.12]; *Z* = 1.51; *p* = 0.13) and 0.96 (95% CI [0.18, 4.99]; *Z* = 0.05; *p* = 0.96), respectively. For toxicities and side effects of grade 3 or higher, the incidence rates of alopecia, pruritus, and dry skin could not be estimated.

##### 3.3.2.2. Taste system

In a systematic meta-analysis focusing on the toxicities and side effects of the targeted drug lenvatinib, we conducted an in-depth exploration of toxicities related to the taste system, primarily including dysgeusia, nasopharyngitis, stomatitis, oral pain, mouth ulceration, and toothache (Figure 4B and Supplementary Table 8).

In toxicities and side effects of any grade, the RR of taste disorders with lenvatinib treatment was significantly elevated to 14.39, with a 95% CI of [4.46, 46.36], a Z-value of 4.47, and a P-value less than 0.00001, indicating that lenvatinib treatment significantly increases the risk of taste disorders. However, for nasopharyngitis, oral pain, mouth ulceration, and toothache, the RRs with lenvatinib treatment did not show statistically significant differences. Specifically, the RR for nasopharyngitis was 0.45, with a 95% CI of [0.11, 1.90] and *p* = 0.28; the RR for stomatitis was 1.71, which although elevated, had a 95% CI of [1.31, 2.24] and a P-value less than 0.0001, suggesting that the strength of association may be influenced by other factors; the RR for oral pain was 5.21, but the 95% CI was extremely wide, ranging from [0.59, 46.30], with *p* = 0.14; the RR for mouth ulceration was extremely low at 0.08, with a 95% CI of [0.00, 1.46] and *p* = 0.09; and the RR for toothache was 3.00, but it was also affected by a wide confidence interval, with a 95% CI of [0.30, 29.85] and *p* = 0.35.

When considering toxicities and side effects of grade 3 or higher (grade ≥3), no lenvatinib-related toxicities and side effects were observed for taste disorders, nasopharyngitis, oral pain, and toothache, thus preventing the calculation of RRs. For stomatitis, although the RR with lenvatinib treatment was elevated to 2.18, the 95% CI was [0.91, 5.24] with *p* = 0.08, failing to demonstrate a significant increase in the risk of grade 3 or higher toxicities and side effects. The RR for mouth ulceration decreased to 0.31 in grade 3 or higher toxicities and side effects, but the 95% CI was similarly wide, ranging from [0.01, 7.90] with *p* = 0.48, indicating uncertainty in the strength of association.

#### 3.3.3. Toxicity of the respiratory system

##### 3.3.3.1. Respiratory, thoracic, and mediastinal

In a systematic meta-analysis on the toxicities and side effects of the targeted drug lenvatinib, we delved into its impact on patients’ respiratory, thoracic, and mediastinal systems, and conducted a comparative analysis with a control group that did not use lenvatinib (Figure 5A and Supplementary Table 9). The following are the key findings:

During the long-term administration of lenvatinib, we observed a series of toxicities and side effects related to the respiratory, thoracic, and mediastinal systems, with certain differences in their occurrence frequency compared to the control group. Specifically, for any grade of toxicities and side effects, there was no significant difference in the incidence rate of dyspnea between the lenvatinib treatment group and the control group, with a RR of 0.95 (95% CI [0.37, 2.44]; *Z* = 0.10; *p* = 0.92). The incidence rate of exertional dyspnea in the lenvatinib treatment group was extremely low and showed no statistical difference compared to the control group (RR 0.18; 95% CI [0.02, 1.57]; *Z* = 1.56; *p* = 0.12). However, the incidence rate of dysphonia was significantly higher in the lenvatinib treatment group compared to the control group, with an RR of 3.23 (95% CI [2.59, 4.04]; *Z* = 10.33; *p* < 0.00001).

Further analyzing the grade ≥3 toxicities and side effects, we found that the incidence rate of dyspnea in the lenvatinib treatment group remained low and showed no significant difference compared to the control group (RR 0.23; 95% CI [0.02, 2.09]; *Z* = 1.31; *p* = 0.19). Notably, no grade ≥3 toxicities and side effects of exertional dyspnea were observed in the lenvatinib treatment group. Regarding dysphonia, although the incidence rate in the lenvatinib treatment group was higher than that in the control group, the difference between the two groups did not reach statistical significance for grade 3 and above due to factors such as limited sample size (RR 3.74; 95% CI [0.63, 22.33]; *Z* = 1.45; *p* = 0.15).

##### 3.3.3.2. Respiratory tract

In the systematic meta-analysis focusing on the toxicities and side effects of the targeted drug lenvatinib, we paid particular attention to its impact within the respiratory tract category (Figure 5B and Supplementary Table 9). Below are the detailed assessment results concerning the toxicities and side effects related to pneumonitis, upper-respiratory-tract infection, and lower-respiratory-tract infection.

In toxicities and side effects of any grade, the RR of pneumonia occurrence with lenvatinib treatment was 1.35, but the 95% CI was wide, ranging from [0.46, 3.95], with a Z-value of 0.55 and a P-value of 0.58. This indicated that the association between lenvatinib and the risk of pneumonia was not significant. Similarly, for upper-respiratory-tract infection, the RR was 2.44, with a 95% CI of [0.59, 10.01], a Z-value of 1.24, and a P-value of 0.22, showing no statistically significant difference. For lower-respiratory-tract infection, the RR was 0.61, with a 95% CI of [0.16, 2.31], a Z-value of 0.73, and a P-value of 0.47. Again, no significant association was observed between lenvatinib treatment and the risk of lower-respiratory-tract infection.

When considering toxicities and side effects of grade ≥3, the RR for pneumonia with lenvatinib treatment increased slightly to 1.51; however, the 95% CI remained wide, ranging from [0.25, 9.13], with a Z-value of 0.45 and a P-value of 0.65. This failed to demonstrate a significant increase in the risk of high-grade pneumonia with lenvatinib. Notably, no cases of upper-respiratory-tract infection were observed among the grade 3 or higher toxicities and side effects, thus preventing the calculation of a RR. For lower-respiratory-tract infection, in grade 3 or higher toxicities and side effects, the RR increased to 4.08, but the 95% CI was equally wide, ranging from [0.44, 37.87], with a Z-value of 1.24 and a P-value of 0.22. This indicated that the association between lenvatinib and the risk of high-grade lower-respiratory-tract infection was also not statistically significant.

#### 3.3.4. Toxicity of the nervous system

##### 3.3.4.1. Nervous system

In the systematic meta-analysis focusing on the toxicities and side effects of the targeted drug lenvatinib, we specifically assessed its long-term impact on patients’ nervous systems, encompassing aspects such as headache, insomnia, lethargy, and posterior reversible encephalopathy syndrome (Figure 6A and Supplementary Table 10). The following is a detailed summary and conclusion of the relevant results:

During long-term treatment with lenvatinib, neurological abnormalities were observed, mainly manifesting as headache, insomnia, lethargy, and posterior reversible encephalopathy syndrome. The incidence rates of these toxicities and side effects exhibited certain differences compared to the control group that did not receive lenvatinib.

Among the any grade toxicities and side effects, the incidence rate of headache was significantly higher in the lenvatinib treatment group compared to the control group, with an RR of 2.50 (95% CI [1.80, 3.48]; *Z* = 5.42; *p* < 0.00001). There was also a trend of increased incidence of insomnia in the lenvatinib treatment group, although it did not reach statistical significance (RR 7.62; 95% CI [0.90, 64.40]; *Z* = 1.87; *p* = 0.06). The incidence rates of lethargy and posterior reversible encephalopathy syndrome did not show significant differences between the lenvatinib treatment group and the control group (RRs were 3.74 and 7.14, respectively; 95% CIs were [0.74, 18.93] and [0.37, 138.75], respectively; Z-values were 1.59 and 1.30, respectively; P-values were 0.11 and 0.19, respectively).

Upon further analysis of grade ≥3 toxicities and side effects, we found that the incidence rate of headache was significantly elevated in the lenvatinib treatment group, with an RR of 7.14 (95% CI [1.61, 31.77]; *Z* = 2.58; *p* = 0.010). Notably, no grade 3 or higher toxicities and side effects of insomnia or lethargy were observed in the lenvatinib treatment group. Regarding posterior reversible encephalopathy syndrome, although the incidence rate was higher in the lenvatinib treatment group compared to the control group, the difference between the two groups did not reach statistical significance at the severe grade 3 or higher level (RR 3.04; 95% CI [0.12, 74.89]; *Z* = 0.68; *p* = 0.50).

##### 3.3.4.2. General

In our systematic meta-analysis focusing on the toxicities and side effects of the targeted drug lenvatinib, we paid particular attention to the drug’s impact on patients’ general condition, especially regarding fatigue/asthenia, fatigue, asthenia, and other symptoms such as pyrexia, cough, peripheral edema, and dry mouth (Figure 6B and Supplementary Table 10).

Regarding fatigue/asthenia, fatigue, and asthenia, the study found that lenvatinib significantly increased the incidence of these symptoms among any grade of toxicities and side effects. Specifically, the RR for fatigue/asthenia was 5.35 with a 95% CI of [3.03, 9.40] and a P-value of less than 0.00001. For fatigue, the RR was 1.49 with a 95% CI of [1.31, 1.68] and a P-value of less than 0.00001. The RR for asthenia was 2.61 with a 95% CI of [1.71, 3.99] and a P-value of less than 0.00001. However, among grade ≥3 toxicities and side effects, while lenvatinib still increased the incidence of fatigue and asthenia (RR for fatigue was 1.64 with a 95% CI of [1.21, 2.21] and *p* = 0.001; RR for asthenia was 3.93 with a 95% CI of [1.14, 13.54] and *p* = 0.03), the impact on fatigue/asthenia did not reach statistical significance (RR was 4.08 with a 95% CI of [0.44, 37.87] and *p* = 0.22).

Furthermore, we also analyzed the impact of lenvatinib on other general symptoms in patients. Across all grades of toxicities and side effects, lenvatinib had no significant effect on the incidence of pyrexia, cough, and dry mouth (RR for pyrexia was 1.07 with a 95% CI of [0.76, 1.52] and *p* = 0.68; RR for cough was 0.84 with a 95% CI of [0.45, 1.57] and *p* = 0.58; RR for dry mouth could not be calculated due to no occurrences of the symptom). However, lenvatinib significantly increased the incidence of peripheral edema (RR was 1.83 with a 95% CI of [1.24, 2.72] and *p* = 0.002). For grade 3 or higher toxicities and side effects, lenvatinib’s impact on pyrexia, cough, peripheral edema, and dry mouth did not reach statistical significance, although there was an increased risk for peripheral edema (RR was 6.05 with a 95% CI of [0.73, 50.45] and *p* = 0.10), and for dry mouth, no patients experienced this grade of the symptom.

#### 3.3.5. Toxicity of the digestive system

##### 3.3.5.1. Gastrointestinal

During the long-term administration of lenvatinib, we observed a series of toxicities and side effects on the gastrointestinal system (Figure 7A and Supplementary Table 11). The incidence of these toxicities and side effects was significantly higher compared to the control group not receiving lenvatinib.

In terms of any grade toxicities and side effects, lenvatinib was associated with nearly a twofold increased risk of diarrhea (RR 1.99; 95% CI [1.76, 2.24]; *p* < 0.00001), as well as significantly increased risks of nausea and vomiting (RR 2.08; 95% CI [1.71, 2.52]; *p* < 0.00001 and RR 3.05; 95% CI [2.37, 3.93]; *p* < 0.00001, respectively). Additionally, there was an increased risk of constipation (RR 1.60; 95% CI [1.24, 2.07]; *p* = 0.0003). However, the increase in the risk of abdominal pain was not significant (RR 1.08; 95% CI [0.87, 1.33]; *p* = 0.49).

When considering toxicities and side effects of grade ≥3, the risk of diarrhea remained significantly increased (RR 1.99; 95% CI [1.42, 2.79]; *p* < 0.0001). Notably, although the increase in risk for constipation was highly significant in the grade ≥3 category (RR 10.07; 95% CI [1.29, 78.82]; *p* = 0.03), its absolute incidence may be low as this result was based on a smaller number of events. In contrast, the increases in risk for abdominal pain, nausea, and vomiting in the grade ≥3 category were not statistically significant.

Apart from the aforementioned common gastrointestinal toxicities and side effects, we also analyzed the impact of lenvatinib on ascites, dyspepsia, fistula formation, and gastrointestinal perforation. In the any grade category, the risks of these toxicities and side effects increased, but none reached statistical significance. Similarly, in the grade ≥3 category, the increases in risk for these toxicities and side effects were also not statistically significant. However, it is noteworthy that, although the incidence of fistula formation and gastrointestinal perforation is low, once they occur, they may have severe consequences for patients.

##### 3.3.5.2. Liver

During long-term lenvatinib treatment, we observed a series of abnormal effects on liver function, specifically manifested as elevated aspartate aminotransferase (AST) levels, increased blood bilirubin levels, elevated lipase levels, and hypercholesterolemia (Figure 7B and Supplementary Table 11). Compared with the control group not receiving lenvatinib, the incidence of these liver function abnormalities was higher in the lenvatinib-treated group. This conclusion is based on comprehensive data analysis from multiple studies.

Specifically, lenvatinib treatment interferes with the normal metabolic processes of hepatocytes, leading to abnormal fluctuations in liver function indicators. Although there are some variations in the reported incidence of liver function abnormalities across studies, overall, the incidence in the lenvatinib group is generally higher than that in the control group.

When assessing toxicities and side reactions of any grade, we found that the RR for overall liver function abnormalities associated with lenvatinib treatment was 1.96 (95% CI [1.33, 2.89]) with a Z-value of 3.40 and a P-value of 0.0007, indicating a significant correlation between lenvatinib treatment and liver function abnormalities. However, for specific liver function indicators such as elevated AST levels, increased blood bilirubin levels, elevated lipase levels, and hypercholesterolemia, although the RRs fluctuated, none reached statistical significance (all P-values > 0.05).

When assessing high-grade (grade ≥ 3) toxicities and side reactions, the RR for overall liver function abnormalities associated with lenvatinib treatment decreased to 1.24 (95% CI [0.66, 2.33]) with a Z-value of 0.67 and a P-value of 0.50, failing to demonstrate a statistically significant difference. Similarly, for specific liver function indicators, the RRs also did not reach statistical significance (all P-values > 0.05). Notably, although the RR for hypercholesterolemia among high-grade toxicities and side reactions was relatively high (2.94), due to the extremely wide confidence interval (95% CI [0.12, 73.93]), this result is unstable and should be interpreted with caution.

#### 3.3.6. Toxicity of the urinary system

During long-term lenvatinib treatment, toxicities and side reactions affecting the renal/urinary system are mainly manifested in three aspects: proteinuria, renal failure, and hematuria (Figure 8 and Supplementary Table 12). The specific results are as follows:

For toxicities and side reactions of any grade, the incidence of proteinuria increased significantly with an RR of 0.12 (95% CI [0.10, 0.14]), a Z-value of 12.40, and a P-value less than 0.00001, indicating a significant correlation between lenvatinib treatment and the occurrence of proteinuria. In contrast, the RR for renal failure was 1.29 (95% CI [0.58, 2.88]) with a Z-value of 0.63 and a P-value of 0.53, failing to show a statistically significant difference. The RR for hematuria was 3.84 (95% CI [1.09, 13.59]) with a Z-value of 2.09 and a P-value of 0.04, suggesting a possible association between lenvatinib treatment and the occurrence of hematuria, although this conclusion should be interpreted with caution.

In toxicities and side reactions of grade ≥3, the RR for proteinuria significantly increased to 2.45 (95% CI [1.91, 3.14]) with a Z-value of 7.07 and a P-value less than 0.00001, further confirming a significant correlation between lenvatinib treatment and severe proteinuria. For renal failure, the RR of high-grade toxicities and side reactions was 1.42 (95% CI [0.45, 4.49]) with a Z-value of 0.59 and a P-value of 0.55, also failing to show a statistically significant difference. Notably, no occurrence of hematuria was observed in high-grade toxicities and side reactions, which may suggest a relatively low risk of severe hematuria associated with lenvatinib treatment.

#### 3.3.7. Endocrine and metabolic toxicity

##### 3.3.7.1. Endocrine

In evaluating the impact of lenvatinib on toxicities and side effects related to the endocrine system in patients, we observed the following results (Figure 9A and Supplementary Table 13):

For toxicities and side effects of any grade, lenvatinib treatment significantly increased the risk of hypothyroidism in patients, with an RR of 7.11, a 95% CI of [5.82, 8.70], a Z-value of 19.12, and a P-value less than 0.00001, indicating a highly statistically significant association. Meanwhile, the association between lenvatinib treatment and increased blood-thyroid-stimulating hormone levels was not statistically significant, with an RR of 1.96, a 95% CI of [0.17, 22.32], a Z-value of 0.54, and a P-value of 0.59. Additionally, lenvatinib treatment also increased the risk of hypocalcemia in patients, with an RR of 2.26, a 95% CI of [1.26, 4.07], a Z-value of 2.72, and a P-value of 0.006, demonstrating a statistically significant association.

In the analysis of toxicities and side effects with a grade of ≥3, although a certain trend was observed in the association between lenvatinib treatment and hypothyroidism in patients, it did not reach statistical significance, with an RR of 2.84, a 95% CI of [0.94, 8.56], a Z-value of 1.86, and a P-value of 0.06. Notably, among the patients included in this study, no significant increase in grade ≥3 toxicities and side effects related to elevated blood-thyroid-stimulating hormone levels was observed with lenvatinib treatment. For hypocalcemia, although lenvatinib treatment increased its risk, this association also did not reach statistical significance, with an RR of 2.39, a 95% CI of [0.49, 11.73], a Z-value of 1.08, and a P-value of 0.28.

##### 3.3.7.2. Metabolism/nutrition

In our systematic meta-analysis focusing on the toxicities and side effects of the targeted drug lenvatinib, we paid particular attention to its impact within the metabolism/nutrition category. Specifically, we conducted a detailed evaluation of its toxicities and side effects on decreased weight, decreased appetite, cachexia, and hyperglycaemia (Figure 9B and Supplementary Table 13).

For decreased weight and decreased appetite, the results showed that lenvatinib significantly increased the risk of these two toxicities and side effects. In any grade toxicities and side effects, the RR for decreased weight was 2.43, with a 95% CI of [2.04, 2.90] and a P-value less than 0.00001, indicating that the risk of weight loss was 2.43 times higher in patients treated with lenvatinib compared to the control group. Similarly, for decreased appetite in any grade toxicities and side effects, the RR was 2.01, with a 95% CI of [1.77, 2.28] and a P-value also less than 0.00001. When the toxicities and side effect grade ≥3, the risks of decreased weight and decreased appetite further increased, with RRs of 3.52 and 3.86, respectively, and both were statistically significant (P-values less than 0.00001).

Furthermore, we also evaluated the impact of lenvatinib on cachexia and hyperglycaemia. However, the results indicated considerable uncertainty in the association between lenvatinib and cachexia. For toxicities and side effects of any grade and grade 3 or higher, the RR for cachexia was 3.02, but the 95% CI was extremely wide ([0.12, 74.62]), and the P-values were both 0.50. This suggested that the result may be influenced by factors such as sample size or study heterogeneity, and no definitive conclusion could be drawn. Regarding hyperglycaemia, lenvatinib showed a trend of reducing the risk in toxicities and side effects of any grade (RR = 0.22, 95% CI = [0.06, 0.83], *p* = 0.03). However, for toxicities and side effects of grade 3 or higher, this association became non-significant (RR = 0.08, 95% CI = [0.00, 1.46], *p* = 0.09), indicating that the impact of lenvatinib on hyperglycaemia may be complex and requires further in-depth study.

#### 3.3.8. Toxicity of the musculoskeletal system

For the combined symptom of musculoskeletal chest pain/arthralgia/myalgia, in terms of any grade toxicities and side effects, the RR was 1.82, with a 95% CI of [1.35, 2.45], a Z-value of 3.96, and a P-value of less than 0.00001, indicating that lenvatinib may increase the risk of these symptoms. However, for grade ≥3 toxicities and side effects, the RR decreased to 0.54, with a 95% CI of [0.20, 1.47], a Z-value of 1.21, and a P-value of 0.23, at which point the association between lenvatinib and these symptoms was no longer significant (Figure 10 and Supplementary Table 14).

For the symptom of musculoskeletal chest pain alone, in any grade toxicities and side effects, the RR was 3.13; however, the 95% CI was wide, ranging from [0.60, 16.31], with a Z-value of 1.36 and a P-value of 0.18, indicating that the result was not yet statistically significant. In grade 3 or higher toxicities and side effects, despite an RR as high as 2.94, the 95% CI was equally very wide, spanning from [0.12, 73.93], with a Z-value of 0.66 and a P-value of 0.51. Therefore, a clear association between lenvatinib and this symptom cannot be established (Figure 10 and Supplementary Table 14).

For the symptoms of arthralgia and myalgia, lenvatinib demonstrated higher RR values in any grade toxicities and side effects, with 8.41 for arthralgia and 7.62 for myalgia (Figure 10 and Supplementary Table 14). Specifically, for arthralgia, the 95% CI was [3.72, 18.99], the Z-value was 5.12, and the P-value was less than 0.00001. For myalgia, the 95% CI was [0.90, 64.40], the Z-value was 1.87, and the P-value was 0.06. However, in grade 3 or higher toxicities and side effects, no AEs of arthralgia were observed. For myalgia, although the RR remained at 2.94, the 95% CI was equally very wide ([0.12, 73.93]), with a Z-value of 0.66 and a P-value of 0.51. Therefore, an association between lenvatinib and these symptoms in grade 3 or higher could not be established.

Additionally, lenvatinib also affected other symptoms within the musculoskeletal/connective tissue category (Figure 10 and Supplementary Table 14). In any grade toxicities and side effects, the RR for back pain was 1.65, but the 95% CI was wide ([0.58, 4.66]), with a Z-value of 0.94 and a P-value of 0.35. The RR for musculoskeletal pain was 7.62, with a 95% CI of [0.90, 64.40], a Z-value of 1.87, and a P-value of 0.06. The RR for pain in extremity was 2.04, with a 95% CI of [0.48, 8.66], a Z-value of 0.97, and a P-value of 0.33. In grade 3 or higher toxicities and side effects, no AEs of back pain were observed. For musculoskeletal pain and pain in extremity, although the RRs were both 2.94, the 95% CIs were equally very wide ([0.12, 73.93]), with Z-values of 0.66 and P-values of 0.51. Therefore, a clear association between lenvatinib and these symptoms in grade 3 or higher could not be established.

#### 3.3.9. Other severe toxicities

In our systematic meta-analysis on the toxicities of the targeted drug lenvatinib, we further evaluated other potential serious toxicities, primarily including death, sepsis, and increased blood lactate dehydrogenase (Figure 11 and Supplementary Table 15).

Among toxicities of any grade, lenvatinib therapy was significantly associated with an increased risk of death. Specifically, the RR for death in the lenvatinib group compared to the control group was 2.80, with a 95% CI of [1.13, 6.98], a Z-value of 2.22, and a P-value of 0.03, indicating that lenvatinib therapy may increase the risk of death in patients. For sepsis, although the RR in the lenvatinib group was as high as 3.02, a definitive association between lenvatinib and sepsis could not be established due to the extremely wide 95% CI of [0.12, 74.62] and a P-value of 0.50. Additionally, lenvatinib therapy was also significantly associated with an increased risk of blood lactate dehydrogenase elevation, with an RR of 7.77, a 95% CI of [1.76, 34.25], a Z-value of 2.71, and a P-value of 0.007, suggesting that lenvatinib may have an impact on the liver or other related metabolic pathways.

When considering toxicities of grade 3 or higher (grade ≥3), the association between lenvatinib therapy and the risks of death and sepsis remained uncertain. Specifically, the RRs for both death and sepsis were 3.02, but with extremely wide 95% CIs of [0.12, 74.62] and P-values of 0.50 for both, suggesting that these results may be influenced by factors such as sample size, patient characteristics, or other unknowns. Notably, no cases of increased blood lactate dehydrogenase were observed among the grade 3 or higher toxicities, which may be due to the lower sensitivity of this marker to lenvatinib therapy or factors such as sample selection.

## 4. Discussion

Lenvatinib has demonstrated promising therapeutic effects in the treatment of various cancers/tumors. However, the accompanying toxicities and side effects are quite significant and should not be overlooked [13–17]. Since its launch and application, clinical physicians and researchers have shown an increasing level of attention to its toxicities and side effects. Although multiple previous RCTs have reported toxicities and side event data for lenvatinib, this information is scattered across different studies and cancer types, lacking a systematic integration and quantitative analysis of the toxicity profile. This makes it difficult for clinicians to accurately balance efficacy and risks when formulating treatment plans, and also fails to provide clear treatment decision-making references for patients.

In this study, a meta-analysis was conducted to comprehensively evaluate any AEs associated with lenvatinib. The results showed that lenvatinib significantly increased the risk of toxicities and side events in patients during the treatment process, especially in terms of treatment-related toxicities and side events, high-grade toxicities and side events (grade ≥3), and dose adjustments due to toxicities and side events. This study included 8 RCTs and 1 non-RCT. Compared with the results of existing single RCTs, the core value of this study lies not only in data integration and toxicity profile characterization, but more importantly in providing key evidence for patient-physician shared decision-making and the optimization of toxicities and side effect management strategies. The specific supplementary value is reflected in the following aspects: 1) Advantage in data integration: It is the first time that toxicity data from 9 high-quality studies (including 8 RCTs and 1 non-RCT) have been subjected to unified quantitative analysis, generating pooled effect sizes for toxicities across various systems. This overcomes the limitations of small sample sizes and inconsistent results in individual studies, and provides more robust evidence-based support for clinical decision-making. 2) Comprehensiveness of toxicity profile: Based on the integrated data from 8 RCTs and 1 non-RCT, and building on previous research reports, this study further systematically characterized adverse reactions involving 9 organ systems, clearly distinguished between toxicities of any grade and grade ≥3, and accurately defined the incidence risk and severity of core toxicities and side effects such as hypertension, palmar-plantar erythrodysesthesia syndrome, and diarrhea. This provides clear targets for formulating ‘early monitoring and timely intervention’ strategies to reduce toxicity. 3) Support for clinical decision-making: It identified the main types of toxicity that lead to dose adjustment and treatment discontinuation (e.g. high-grade hypertension, severe diarrhea). On the one hand, this helps physicians communicate treatment risks with patients in advance, enabling patient-participated shared decision-making. On the other hand, it provides actionable evidence for individualized medication (e.g. dose adjustment protocols for high-risk patients) and early intervention (e.g. prophylactic use of antihypertensive drugs).

Notably, among the 9 studies included in this research, 3 used placebo as the control (all were RCTs). Therefore, these toxicity risk assessment results are more applicable for comparison with no treatment or best supportive care, and cautious interpretation is required when weighing efficacy against toxicity in comparison with other active anticancer drugs. This characteristic is directly related to the accuracy of patient-physician shared decision-making: when selecting treatment regimens, patients and clinicians not only need to understand the toxicities and side reactions of lenvatinib compared with ‘no treatment’ (as clearly established in this study), but also must integrate toxicity comparison data between lenvatinib and other similar targeted drugs from other studies to make the most suitable individualized choice for patients. This also represents an important reference dimension provided by this study for clinical decision-making.

Secondly, this study comprehensively evaluated the toxicities and side effects of lenvatinib in nine aspects, namely, vascular toxicities related to the circulatory system (vascular toxicities, blood system, and heart), toxicities of the skin and its appendages (skin/subcutaneous tissue and taste system), toxicities of the respiratory system (respiratory, thoracic, and mediastinal and respiratory tract), toxicities of the nervous system (nervous system and general), toxicities of the digestive system (gastrointestinal and liver), toxicities of the urinary system, toxicities of the endocrine and metabolic system (endocrine and metabolism/nutrition), toxicities of the musculoskeletal system, and other severe toxicities. The evaluation levels were subdivided into two levels: any grade and grade ≥3. This stratification not only clearly presents the risk differences of toxicities across different systems, but also provides a basis for formulating hierarchical toxicity reduction strategies—for instance, enhanced monitoring and intervention are required for grade ≥3 hypertension, while basic nursing measures can be adopted for grade 1–2 rash.

The results indicated that lenvatinib exhibits a broad spectrum of toxicities affecting multiple systems. In the vascular system, lenvatinib significantly increased the incidence of hypertension and hemorrhage events. It is important to note that there was considerable variability in the prevalence of hypertension across the included studies. This may be associated with factors such as baseline patient characteristics (e.g. proportion of patients with a history of essential hypertension, age distribution), lenvatinib dosage and treatment duration, and frequency of blood pressure monitoring. Therefore, the pooled results of hypertension in this study should be interpreted with caution. In terms of skin/subcutaneous tissue, the primary manifestations were palmar-plantar erythrodysesthesia syndrome and rash. The respiratory, thoracic, and mediastinal toxicities included dyspnea and hoarseness. In the nervous system, there was a notable increase in the incidence of headache. The blood system was affected mainly by thrombocytopenia and an increased risk of hemorrhage. The gastrointestinal system demonstrated significant toxicities, including diarrhea, nausea, vomiting, and constipation. Additionally, lenvatinib led to a substantial increase in the incidence and severity of proteinuria in the urinary system, an overall increase in liver function abnormalities, and a significant elevation in symptoms such as fatigue/asthenia. In the endocrine system, lenvatinib therapy increased the risk of hypothyroidism and hypocalcemia. In the musculoskeletal system, lenvatinib may induce a variety of toxicities. In the metabolism/nutrition category, the primary toxicities were weight loss and decreased appetite. In the cardiac system, lenvatinib showed a trend of increasing the risk of heart failure. Furthermore, lenvatinib significantly increased the risk of mortality. The value of these results lies in the following aspects: 1) Providing specific basis for patient-physician shared decision-making: Patients can clearly understand the potential toxicity risks of treatment (e.g. whether quality of life-impairing palmar-plantar erythrodysesthesia syndrome may occur), while physicians can assess tolerance based on patients’ underlying diseases (e.g. history of hypertension) to jointly select a treatment regimen. 2) Offering targets for optimizing toxicity reduction strategies: For high-incidence and severe toxicities (e.g. hypertension, proteinuria), clinical practice can establish a full-process management plan featuring ‘pre-treatment screening, regular monitoring during treatment, and timely management of toxicities’, so as to reduce the interference of toxicities on treatment. 3) Highlighting the importance of patients’ self-reported treatment efficacy and toxicities: Subjective symptoms identified in this study, such as fatigue and decreased appetite, can only be detected in a timely manner relying on patients’ active reporting. This suggests that future studies should strengthen the inclusion of patient-reported outcomes to improve the toxicity assessment system.

However, this study also has certain limitations [18–20]. First, among the 9 included studies, 3 used placebo as the control (all were RCTs). This means that the conclusion of increased lenvatinib-related toxicity risk drawn in this study mainly reflects the comparison with no treatment, rather than a direct head-to-head comparison with other active anticancer treatment regimens. To a certain extent, this limits the trade-off decision-making between different treatment options for patients and physicians, which constitutes a major limitation of this study. Second, the relatively limited number of studies and sample size included may have affected the broad applicability of the results [21]. Additionally, this limitation leads to insufficient statistical power for analyzing some rare toxicities (e.g. severe heart failure), making it difficult to support the formulation of more precise toxicity reduction strategies. Third, variations exist in patient characteristics, treatment regimens, and assessment criteria across different studies. These variations may exert a certain impact on the interpretation of results, and also pose challenges to the promotion of standardized toxicity reduction strategies based on the findings of this study [22]. In addition, this study assessed potential publication bias using funnel plots combined with Egger’s test or Begg’s test. The results revealed a certain degree of publication bias, which may lead to the overrepresentation of positive findings and thus undermine the persuasiveness of the study results.

## 5. Conclusions

This study systematically integrated data from 9 high-quality studies *via* meta-analysis, comprehensively mapped the toxicities and side effect profile of lenvatinib involving 9 organ systems, and clarified the incidence risks of toxicities of any grade and grade ≥ 3. It provides critical evidence-based support for formulating clinical toxicity mitigation strategies featuring ‘early monitoring and timely intervention’ as well as for patient-physician shared decision-making. Considering the study limitations, the conclusions are more applicable to the comparison of toxicity risks between lenvatinib and placebo (or no treatment), and should be interpreted with caution when weighing different active treatment regimens, with integration of evidence from other studies.

Therefore, to more comprehensively assess the toxicities of lenvatinib, more high-quality clinical trials with large sample sizes need to be conducted in the future. Efforts should focus on the following directions to address clinical needs: 1) Conduct head-to-head studies comparing lenvatinib with other active anticancer drugs to supplement toxicity comparison data between different treatment regimens, thereby providing more comprehensive evidence for patient-physician shared decision-making; 2) Strictly control patient enrollment criteria and treatment regimens to reduce study heterogeneity, and provide reliable evidence for formulating standardized and generalizable toxicity reduction strategies; 3) Strengthen the collection and analysis of patient-reported outcomes, conduct in-depth studies on the impact of subjective toxicities (such as fatigue and pain) on patients’ quality of life, improve the toxicity assessment system, and promote patient-centered toxicity management; 4) Further explore the mechanisms underlying lenvatinib-induced toxicities and side effects (e.g. the association between hypertension development and endothelial function), so as to provide a theoretical basis for developing targeted toxicity-reducing drugs and optimizing medication regimens (such as dose timing adjustment). Ultimately, this will help reduce toxicities and side effects and improve patients’ treatment efficacy and quality of life. 5) Currently, most clinical studies reporting the toxicities and side effects of lenvatinib are retrospective, with limited evidence strength. Therefore, there is an urgent need to conduct large-sample randomized prospective studies to obtain more reliable toxicity data and provide higher-level evidence-based support for clinical practice. These represent important directions for future research and also urgent needs in clinical medication practice. (The complete abbreviations are shown in Table 2.)

**Table 2.** List of abbreviations (according to alphabetical order).

Abbreviations	Full name
AEs	adverse events
AST	aspartate aminotransferase
CI	confidence interval
FDA	Food and Drug Administration
FGFR	Fibroblast Growth Factor Receptor
KIT	K-Cell Receptor Tyrosine Kinase
NR	Not Reported
PDGFRα	Platelet - Derived Growth Factor Receptor Alpha
RCT	randomized controlled trial
RET	Rearranged during Transfection
RR	risk ratio
TEAEs	treatment-emergent adverse events
TRAEs	treatment-related adverse events
TSH	thyroid-stimulating hormone
VEGFR	Vascular Endothelial Growth Factor Receptor
WBC	white blood cell

## Supplementary Material

Supplemental Material

## Data Availability

Article data available from the corresponding author upon reasonable request.
