# Supplementary material for: Systematic meta-analysis of the toxicities and side effects of the targeted drug lenvatinib
Source: Ann Med. 2025 Dec 24;58(1):2598935. doi: 10.1080/07853890.2025.2598935 (PMC12777875; doi:10.1080/07853890.2025.2598935)
Supplement: Supplemental Material [file IANN_A_2598935_SM0031.zip › suppl_data/Supplementary Figure caption.docx]

**Supplementary Figure 1. Assessment of Bias Risk in RCTs**

A. Risk of bias graph.

B. Risk of bias summary.

**Supplementary Figure 2. Evaluation of Publication Bias of Lenvatinib for Any AEs**

A. Meta-analysis of any AEs, any TRAEs, total TEAEs and total treatment-related TEAEs

B. Meta-analysis of lenvatinib for other any AEs (any grade) in patients

**Supplementary Figure 3. Evaluation of Publication Bias of Lenvatinib on Circulatory System-Related Toxicities**

**A. Vascular Toxicities**

It presents the publication bias evaluation results of lenvatinib-induced vascular toxicities, with toxicity grades (any grade, grade ≥ 3) on the horizontal axis and specific toxicity types (hypertension, hemorrhagic events) on the vertical axis.

**B. Blood System**

It presents the publication bias evaluation results of lenvatinib-induced blood system toxicities, with toxicity grades (any grade, grade ≥ 3) on the horizontal axis and specific toxicity types (hypertriglyceridaemia, thrombocytopenia, epistaxis, anaemia, hemorrhag) on the vertical axis.

**C. Heart**

It presents the publication bias evaluation results of lenvatinib-induced heart toxicities, with toxicity grades (any grade, grade ≥ 3) on the horizontal axis and specific toxicity types (cardiac failure, arterial thromboembolic event, QT prolongation) on the vertical axis.

**Supplementary Figure 4. Evaluation of Publication Bias of Lenvatinib on Toxicities of the Skin and its Appendages**

**A. Skin/Subcutaneous Tissue**

It presents the publication bias evaluation results of lenvatinib-induced skin/subcutaneous tissue toxicities, with toxicity grades (any grade, grade ≥ 3) on the horizontal axis and specific toxicity types (palmar-plantar erythrodysesthesia syndrome, rash, alopecia, pruritus, dry skin) on the vertical axis.

**B. Taste System**

It presents the publication bias evaluation results of lenvatinib-induced taste system toxicities, with toxicity grades (any grade, grade ≥ 3) on the horizontal axis and specific toxicity types (dysgeusia, nasopharyngitis, stomatitis, oral pain, mouth ulceration, toothache) on the vertical axis.

**Supplementary Figure 5. Evaluation of Publication Bias of Lenvatinib on Toxicities of the Respiratory System**

**A. Respiratory, Thoracic, and Mediastinal**

It presents the publication bias evaluation results of lenvatinib-induced respiratory, thoracic, and mediastinal toxicities, with toxicity grades (any grade, grade ≥ 3) on the horizontal axis and specific toxicity types (dyspnoea, exertional dyspnoea, dysphonia) on the vertical axis.

**B. Respiratory Tract**

It presents the publication bias evaluation results of lenvatinib-induced respiratory tract toxicities, with toxicity grades (any grade, grade ≥ 3) on the horizontal axis and specific toxicity types (pneumonitis, upper-respiratory-tract infection, lower-respiratory-tract infection) on the vertical axis.

**Supplementary Figure 6. Evaluation of Publication Bias of Lenvatinib on Toxicities of the Nervous System**

**A. Nervous System**

It presents the publication bias evaluation results of lenvatinib-induced nervous system toxicities, with toxicity grades (any grade, grade ≥ 3) on the horizontal axis and specific toxicity types (headache, insomnia, lethargy, posterior reversible encephalopathy syndrome) on the vertical axis.

**B. General**

It presents the publication bias evaluation results of lenvatinib-induced general toxicities, with toxicity grades (any grade, grade ≥ 3) on the horizontal axis and specific toxicity types (fatigue/asthenia, fatigue, asthenia, pyrexia, cough, peripheral edema, dry mouth) on the vertical axis.

**Supplementary Figure 7. Evaluation of Publication Bias of Lenvatinib on Toxicities of the Digestive System**

**A. Gastrointestinal**

It presents the publication bias evaluation results of lenvatinib-induced gastrointestinal toxicities, with toxicity grades (any grade, grade ≥ 3) on the horizontal axis and specific toxicity types (diarrhea, abdominal pain, nausea, vomiting, constipation, ascites, dyspepsia, fistula formation, gastrointestinal perforation) on the vertical axis.

**B. Liver**

It presents the publication bias evaluation results of lenvatinib-induced liver toxicities, with toxicity grades (any grade, grade ≥ 3) on the horizontal axis and specific toxicity types (liver toxicity, elevated aspartate aminotransferase, increased blood bilirubin, lipase level increased, hypercholesterolaemia) on the vertical axis.

**Supplementary Figure 8. Evaluation of Publication Bias of Lenvatinib on Toxicities of the Urinary System**

It presents the publication bias evaluation results of lenvatinib-induced urinary system toxicities, with toxicity grades (any grade, grade ≥ 3) on the horizontal axis and specific toxicity types (proteinuria, renal failure, hematuria) on the vertical axis.

**Supplementary Figure 9. Evaluation of Publication Bias of Lenvatinib on Toxicities of the Endocrine and Metabolic Systems**

**A. Endocrine**

It presents the publication bias evaluation results of lenvatinib-induced endocrine toxicities, with toxicity grades (any grade, grade ≥ 3) on the horizontal axis and specific toxicity types (hypothyroidism, increased blood-thyroid-stimulating hormone level, hypocalcemia) on the vertical axis.

**B. Metabolism/Nutrition**

It presents the publication bias evaluation results of lenvatinib-induced metabolism/nutrition toxicities, with toxicity grades (any grade, grade ≥ 3) on the horizontal axis and specific toxicity types (decreased weight, decreased appetite, cachexia, hyperglycaemia) on the vertical axis.

**Supplementary Figure 10. Evaluation of Publication Bias of Lenvatinib on Toxicities of the Musculoskeletal System**

It presents the publication bias evaluation results of lenvatinib-induced musculoskeletal system toxicities, with toxicity grades (any grade, grade ≥ 3) on the horizontal axis and specific toxicity types (musculoskeletal chest pain/arthralgia/myalgia, musculoskeletal chest pain, arthralgia, myalgia, back pain, musculoskeletal pain, pain in extremity) on the vertical axis.

**Supplementary Figure 11. Evaluation of Publication Bias of Lenvatinib on Other Severe Toxicities**

It presents the publication bias evaluation results of lenvatinib-induced other severe toxicities, with toxicity grades (any grade, grade ≥ 3) on the horizontal axis and specific toxicity types (death, sepsis, blood lactate dehydrogenase increased) on the vertical axis.
