# Supplementary material for: Systematic meta-analysis of the toxicities and side effects of the targeted drug lenvatinib
Source: Ann Med. 2025 Dec 24;58(1):2598935. doi: 10.1080/07853890.2025.2598935 (PMC12777875; doi:10.1080/07853890.2025.2598935)
Supplement: Supplemental Material [file IANN_A_2598935_SM0031.zip › suppl_data/Supplementary Table 1.docx]

**Supplementary Table 1. Search Strategy**

**（from database inception to Dec 31, 2024）**

| **Databases** | | **Searching terms** |
| --- | --- | --- |
| English-language | PubMed | 1.(Lenvatinib[MeSH Terms]) OR (Lenvatinib)) AND (("1963/01/01"[Date - Publication] : "2024/12/31"[Date - Publication])  2. "toxic"[All Fields] OR "toxical"[All Fields] OR "toxically"[All Fields] OR "toxicant"[All Fields] OR "toxicant s"[All Fields] OR "toxicants"[All Fields] OR "toxicated"[All Fields] OR "toxication"[All Fields] OR "toxicities"[All Fields] OR "toxicity"[MeSH Subheading] OR "toxicity"[All Fields] OR "toxicity s"[All Fields] OR "toxics"[All Fields] OR ("adverse effects"[MeSH Subheading] OR ("adverse"[All Fields] AND "effects"[All Fields]) OR "adverse effects"[All Fields] OR ("side"[All Fields] AND "effects"[All Fields]) OR "side effects"[All Fields]) OR (("adverse"[All Fields] OR "adversely"[All Fields] OR "adverses"[All Fields]) AND ("reaction"[All Fields] OR "reaction s"[All Fields] OR "reactions"[All Fields]))) AND 1963/01/01:2024/12/31[Date - Publication]  3. 1 and 2 |
|  | Embase | 1. ('lenvatinib'/exp OR lenvatinib OR lenvatinib:ti,ab,kw) AND ([cochrane review]/lim OR [controlled clinical trial]/lim OR [systematic review]/lim OR [randomized controlled trial]/lim OR [meta analysis]/lim) AND [<1966-2024]/py AND [31-12-2024]/sd  2.(toxicity OR (side AND effects)) AND adverse AND reactions AND ([cochrane review]/lim OR [controlled clinical trial]/lim OR [systematic review]/lim OR [randomized controlled trial]/lim OR [meta analysis]/lim) AND [<1966-2024]/py AND [31-12-2024]/sd  3. 1 and 2 |
|  | Cochrane Library | 1. (Lenvatinib):ti,ab,kw OR (Lenvatinib) with Cochrane Library publication date Between Jan 1963 and Dec 2024, in Cochrane Reviews, Cochrane Protocols, Trials, Clinical Answers, Editorials, Special Collections 2. (toxicity):ti,ab,kw OR (side effects):ti,ab,kw OR (adverse reactions):ti,ab,kw with Cochrane Library publication date Between Jan 1963 and Dec 2024, in Cochrane Reviews, Cochrane Protocols, Trials, Clinical Answers, Editorials, Special Collections 3. 1and 2 |
| Chinese-language | CNKI | 1. (主题:仑伐替尼) OR (篇关摘:仑伐替尼(模糊)) OR (关键词:仑伐替尼(模糊)) OR(篇名:仑伐替尼(模糊))OR(全文:仑伐替尼(模糊))OR(摘要:仑伐替尼(模糊))OR (小标题:仑伐替尼(模糊)) and(发表时间:(1915-01-01-2024-12-31)  2. (主题:毒副作用)OR(篇关摘:毒副作用(模糊))OR(关键词:毒副作用(模糊))OR(篇名:毒副作用(模糊))OR(全文:毒副作用(模糊))OR(摘要:毒副作用(模糊))OR(小标题:毒副作用(模糊)) and(发表时间:(1915-01-01-2024-12-31)  3. 1 and 2 |
|  | Wanfang | 1. 全部:(仑伐替尼)) and 发表时间:1900-2024  2. 全部:(毒副作用) and 发表时间:1900-2024  3. 1 and 2 |
