# Supplementary material for: Systematic meta-analysis of the toxicities and side effects of the targeted drug lenvatinib
Source: Ann Med. 2025 Dec 24;58(1):2598935. doi: 10.1080/07853890.2025.2598935 (PMC12777875; doi:10.1080/07853890.2025.2598935)
Supplement: Supplemental Material [file IANN_A_2598935_SM0031.zip › suppl_data/Supplementary Table 10.docx]

**Supplementary Table 10. Meta-analysis of the Toxicity of Lenvatinib to the Nervous System**

| **Author (year)** | **Any Grade** | | | | | | | | | | **Grade ≥ 3** | | | | | | | | | |
| --- | --- | --- | --- | --- | --- | --- | --- | --- | --- | --- | --- | --- | --- | --- | --- | --- | --- | --- | --- | --- |
|  | **Nervous System n/N (%)** | | | | **General n/N (%)** | | | | | | **Nervous System n/N (%)** | | | | **General n/N (%)** | | | | | |
|  | **Headache** | **Insomnia** | **Lethargy** | **Posterior Reversible Encephalopathy Syndrome** | **Fatigue** | **Asthenia** | **Pyrexia** | **Cough** | **Peripheral Edema** | **Dry Mouth** | **Headache** | **Insomnia** | **Lethargy** | **Posterior Reversible Encephalopathy Syndrome** | **Fatigue** | **Asthenia** | **Pyrexia** | **Cough** | **Peripheral Edema** | **Dry Mouth** |
| Casadei-Gardini et al. (2023) | NR | NR | NR | NR | 431/1343 (32.1%) vs 214/864 (24.8%) | NR | NR | NR | NR | NR | NR | NR | NR | NR | 62/1343 (4.7%) vs 16/864 (1.9%) | NR | NR | NR | NR | NR |
| Haddad et al. (2017) | NR | NR | NR | NR | 85/261 (32.6%) vs 4/131 (3.1%) | | NR | NR | NR | NR | NR | NR | NR | NR | NR | | NR | NR | NR | NR |
| Kiyota et al. (2017) | 98/379 (25.9%) vs 11/204 (5.4%) | NR | NR | NR | 143/379 (37.3%) vs 35/204 (17.2%) | 80/379 (21.1%) vs 19/204 (9.3%) | NR | NR | NR | NR | 9/379 (2.4%) vs 0/204 (0%) | NR | NR | NR | 16/379 (4.2%) vs 2/204 (1.0%) | 14/379 (3.7%) vs 2/204 (1.0%) | NR | NR | NR | NR |
| Kudo et al. (2018) | NR | NR | NR | NR | 141/476 (29.6%) vs 119/475 (25.1%) | NR | NR | NR | NR | NR | NR | NR | NR | NR | 18/476 (3.8%) vs 17/475 (3.6%) | NR | NR | NR | NR | NR |
| Matsubara et al. (2024) | NR | NR | NR | NR | 35/241 (14.5%) vs 29/242 (12.0%) | 29/241 (12.0%) vs 12/242 (5.0%) | NR | NR | NR | NR | NR | NR | NR | NR | 6/241 (2.5%) vs 5/242 (2.1%) | 4/241 (1.7%) vs 1/242 (0.41%) | NR | NR | NR | NR |
| Motzer et al. (2015) | 13/52 (25.0%) vs 5/50 (10.0%) | 7/52 (13.5%) vs 1/50 (2.0%) | 7/52 (13.5%)vs 2/50 (4.0%) | NR | 26/52 (50%) vs 19/50 (38.0%) | | 5/52 (9.6%) vs 5/50 (10.0%) | 9/52 (17.3%) vs 15/50 (30.0%) | 8/52 (15.4%) vs 9/50 (18.0%) | 6/52 (11.5%) vs 3/50 (6.0%) | 2/52 (3.8%) vs 1/50 (2.0%) | 0/52 (0%) vs 0/50 (0%) | 0/52 (0%)vs 0/50 (0%) | NR | 4/52 (7.7%) vs 1/50 (2.0%) | | 0/52 (0%) vs 1/50 (2.0%) | 1/52 (1.9%) vs 0/50 (0%) | 0/52 (0%) vs 0/50 (0%) | 0/52 (0%) vs 0/50 (0%) |
| Nair et al. (2021) | 49/476 (10%) vs 38/475 (8%) | NR | NR | NR | 211/476 (44%) vs 171/475 (36%) | NR | 73/476 (15%) vs 68/475 (14%) | NR | 68/476 (14%) vs 35/475 (7%) | NR | 6/476 (1%) vs 0/475 (8%) | NR | NR | NR | 35/476 (7%) vs 30/475 (6%) | NR | 0/476 (0%) vs 1/475 (0.2%) | NR | 6/476 (1%) vs 1/475 (0.2%) | NR |
| Yang et al. (2024) | NR | NR | NR | 3/309 (1.0%) vs 0/312 (0%) | NR | NR | NR | NR | NR | NR | NR | NR | NR | 1/309 (0.3%) vs 0/312 (0%) | NR | NR | NR | NR | NR | NR |
| Zheng et al. (2021) | NR | NR | NR | NR | NR | NR | NR | 22/103 (21.4%) vs 8/48 (16.7%) | NR | NR | NR | NR | NR | NR | NR | NR | NR | 0/103 (0%) vs 0/48 (0%) | NR | NR |

NR: Not Reported.
