# Supplementary material for: Systematic meta-analysis of the toxicities and side effects of the targeted drug lenvatinib
Source: Ann Med. 2025 Dec 24;58(1):2598935. doi: 10.1080/07853890.2025.2598935 (PMC12777875; doi:10.1080/07853890.2025.2598935)
Supplement: Supplemental Material [file IANN_A_2598935_SM0031.zip › suppl_data/Supplementary Table 11.docx]

**Supplementary Table 11. Meta-analysis of the Toxicity of Lenvatinib to the Digestive System**

| **Author (year)** | **Any Grade** | | | | | | | | | | | | | **Grade ≥ 3** | | | | | | | | | | | | |
| --- | --- | --- | --- | --- | --- | --- | --- | --- | --- | --- | --- | --- | --- | --- | --- | --- | --- | --- | --- | --- | --- | --- | --- | --- | --- | --- |
|  | **Gastrointestinal n/N (%)** | | | | | | | | | **Liver n/N (%)** | | | | **Gastrointestinal n/N (%)** | | | | | | | | | **Liver n/N (%)** | | | |
|  | **Diarrhea** | **Abdominal Pain** | **Nausea** | **Vomiting** | **Constipation** | **Ascites** | **Dyspepsia** | **Fistula Formation** | **Gastrointestinal Perforation** | **Elevated AST** | **Increased Blood Bilirubin** | **Lipase Level Increased** | **Hypercholesterolaemia** | **Diarrhea** | **Abdominal Pain** | **Nausea** | **Vomiting** | **Constipation** | **Ascites** | **Dyspepsia** | **Fistula Formation** | **Gastrointestinal Perforation** | **Elevated AST** | **Increased Blood Bilirubin** | **Lipase Level Increased** | **Hypercholesterolaemia** |
| Casadei-Gardini et al. (2023) | 278/1343 (20.7%) vs 76/864 (8.8%) | NR | NR | NR | NR | NR | NR | NR | NR | NR | NR | NR | NR | 29/1343 (2.2%) vs 6/864 (0.7%) | NR | NR | NR | NR | NR | NR | NR | NR | NR | NR | NR | NR |
| Haddad et al. (2017) | 184/261 (70.5%) vs 8/131 (6.1%) | NR | NR | NR | NR | NR | NR | NR | NR | NR | NR | NR | NR | NR | NR | NR | NR | NR | NR | NR | NR | NR | NR | NR | NR | NR |
| Kiyota et al. (2017) | 220/379 (58.0%) vs 16/ 204 (7.8%) | NR | 157/379 (41.4%) vs 26/204 (12.7%) | 114/379  (30.1%) vs 13/204 (6.4%) | NR | NR | NR | NR | NR | NR | NR | NR | NR | 27/379 (7.1%) vs 0/ 204 (7.8%) | NR | 7/379 (1.8%) vs 1/204 (0.5%) | 7/379  (1.8%) vs 0/204 (0%) | NR | NR | NR | NR | NR | NR | NR | NR | NR |
| Kudo et al. (2018) | 184/476 (38.7%) vs 220/475 (46.3%) | 81/476 (17.0%) vs 87/475 (18.3%) | 93/476 (19.5%) vs 68/475 (14.3%) | 77/476 (16%) vs 36/475 (8%) | 76/476 (16.2%) vs 52/475 (10.9%) | NR | NR | NR | NR | 65/476 (13.7%) vs 80/475 (16.8%) | 71/476 (14.9%) vs 63/475 (13.3%) | NR | NR | 20/476 (4.2%) vs 20/475 (4.2%) | 8/476 (1.7%) vs 13/475 (2.7%) | 4/476 (0.8%) vs 4/475 (0.8%) | 6/476 (1.3%) vs 5/475 (1.1%) | 3/476 (0.6%) vs 0/475 (0%) | NR | NR | NR | NR | 24/476 (5.0%) vs 38/475 (8.0%) | 31/476 (6.5%) vs 23/475 (4.8%) | NR | NR |
| Matsubara et al. (2024) | 50/241 (20.7%) vs 25/242 (10.3%) | NR | 28/241 (11.6%) vs 20/242 (8.3%) | NR | NR | NR | NR | NR | NR | NR | NR | 29/241 (12.0%) vs 17/242 (7.0%) | NR | 11/241 (4.6%) vs 3/242 (1.2%) | NR | 2/241 (0.8%) vs 1/242 (0.4%) | NR | NR | NR | NR | NR | NR | NR | NR | 15/241 (6.2%) vs 11/242 (4.5%) | NR |
| Motzer et al. (2015) | 37/52 (71.2%) vs 17/50 (34.0%) | 16/52 (30.8%) vs 5/50 (10.0%) | 32/52 (61.5%) vs 8/50 (16.0%) | 20/52 (38.5%) vs 5/50 (10.0%) | 19/52 (36.5%) vs 9/50 (18.0%) | NR | 6/52 (11.5%) vs 5/50 (10.0%) | NR | NR | NR | NR | NR | 6/52 (11.5%) vs 8/50 (16.0%) | 6/52 (11.5%) vs 1/50 (2.0%) | 2/52 (3.8%) vs 0/50 (0%) | 4/52 (7.7%) vs 0/50 (0%) | 2/52 (3.8%) vs 0/50 (0%) | 0/52 (0%) vs 0/50 (0%) | NR | 1/52 (1.9%) vs 0/50 (0%) | NR | NR | NR | NR | NR | 1/52 (1.9%) vs 0/50 (0%) |
| Nair et al. (2021) | 187/476 (39%) vs 220/475 (46%) | 144/476 (30%) vs 135/475 (28%) | 97/476 (20%) vs 68/475 (14%) | 78/476 (16%) vs 38/475 (8%) | 78/476 (16%) vs 54/475 (11%) | 73/476 (15%) vs 54/475 (11%) | NR | NR | NR | NR | NR | NR | NR | 21/476 (4%) vs 21/475 (4%) | 16/476 (3%) vs 21/475 (4%) | 6/476 (1%) vs 6/475 (1%) | 6/476 (1%) vs 6/475 (1%) | 6/476 (1%) vs 0/475 (0%) | 21/476 (4%) vs 16/475 (3%) | NR | NR | NR | NR | NR | NR | NR |
| Yang et al. (2024) | NR | NR | NR | NR | NR | NR | NR | 5/309 (1.6%) vs 0/312 (0%) | 4/309 (1.3%) vs 1/312 (0.3%) | 87/309 (28.2%) vs 52/312 (16.7%) | | | | NR | NR | NR | NR | NR | NR | NR | 4/309 (1.3%) vs 0/312 (0%) | 0/309 (0%) vs 1/312 (0.3%) | 23/309 (7.4%) vs 19/312 (6.1%) | | | |
| Zheng et al. (2021) | 51/103 (49.5%) vs 2/48 (4.2%) | NR | NR | NR | NR | NR | NR | NR | NR | NR | NR | NR | NR | 7/103 (6.8%) vs 0/48 (0%) | NR | NR | NR | NR | NR | NR | NR | NR | NR | NR | NR | NR |

NR: Not Reported.
