# Supplementary material for: Systematic meta-analysis of the toxicities and side effects of the targeted drug lenvatinib
Source: Ann Med. 2025 Dec 24;58(1):2598935. doi: 10.1080/07853890.2025.2598935 (PMC12777875; doi:10.1080/07853890.2025.2598935)
Supplement: Supplemental Material [file IANN_A_2598935_SM0031.zip › suppl_data/Supplementary Table 12.docx]

**Supplementary Table 12. Meta-analysis of the Toxicity of Lenvatinib to the Urinary System**

| **Author (year)** | **Any Grade** | | | **Grade ≥ 3** | | |
| --- | --- | --- | --- | --- | --- | --- |
|  | **Proteinuria**  **n/N (%)** | **Renal Failure**  **n/N (%)** | **Hematuria**  **n/N (%)** | **Proteinuria**  **n/N (%)** | **Renal Failure**  **n/N (%)** | **Hematuria**  **n/N (%)** |
| Casadei-Gardini et al. (2023) | 327/1343 (24.4%)vs 239/864 (27.6%) | NR | NR | 96/1343 (7.1%) vs 53/864 (6.1%) | NR | NR |
| Haddad et al. (2017) | 74/261 (28.4%)vs 0/131 (0%) | NR | NR | NR | NR | NR |
| Kiyota et al. (2017) | 112/379 (29.6%) vs 3/204 (1.5%) | NR | NR | 34/379 (9.0%) vs 0/204 (0%) | NR | NR |
| Kudo et al. (2018) | 117/476 (24.6%) vs 54/475 (11.4%) | NR | NR | 27/476 (5.7%) vs 8/475 (1.7%) | NR | NR |
| Matsubara et al. (2024) | 91/241 (37.8%) vs 45/242 (18.6%) | 2/241 (0.8%) vs 3/242 (1.2%) | NR | 28/241 (11.6%) vs 8/242 (3.3%) | 2/241 (0.8%) vs 2/242 (0.8%) | NR |
| Motzer et al. (2015) | 16/52 (30.8%) vs 7/50 (14.0%) | NR | NR | 10/52 (19.2%) vs 1/50 (2.0%) | NR | NR |
| Nair et al. (2021) | 125/476 (26%) vs 57/475 (12%) | NR | NR | 30/476 (6%) vs 11/475 (2%) | NR | NR |
| Yang et al. (2024) | 99/309 (32.0%) vs 34/312 (10.9%) | 12/309 (3.9%) vs 8/312 (2.6%) | NR | 19/309 (6.1%) vs 4/312 (1.3%) | 5/309 (1.6%) vs 3/312 (1.0%) | NR |
| Zheng et al. (2021) | 83/103 (80.6%) vs 4/48 (8.3%) | NR | 21/103 (20.4%) vs 3/48 (6.3%) | 24/103 (23.3%) vs 0/48 (0%) | NR | 0/103 (0%) vs 0/48 (0%) |

NR: Not Reported.
