# Supplementary material for: Systematic meta-analysis of the toxicities and side effects of the targeted drug lenvatinib
Source: Ann Med. 2025 Dec 24;58(1):2598935. doi: 10.1080/07853890.2025.2598935 (PMC12777875; doi:10.1080/07853890.2025.2598935)
Supplement: Supplemental Material [file IANN_A_2598935_SM0031.zip › suppl_data/Supplementary Table 13.docx]

**Supplementary Table 13. Meta-analysis of the Endocrine and Metabolic Toxicity of Lenvatinib**

| **Author (year)** | **Any Grade** | | | | | | | **Grade ≥ 3** | | | | | | |
| --- | --- | --- | --- | --- | --- | --- | --- | --- | --- | --- | --- | --- | --- | --- |
|  | **Endocrine n/N (%)** | | | **Metabolism/Nutrition n/N (%)** | | | | **Endocrine n/N (%)** | | | **Metabolism/Nutrition n/N (%)** | | | |
|  | **Hypothyroidism** | **Increased Blood-Thyroid-Stimulating Hormone Level** | **Hypocalcemia** | **Decreased Weight** | **Decreased Appetite** | **Cachexia** | **Hyperglycaemia** | **Hypothyroidism** | **Increased Blood-Thyroid-Stimulating Hormone Level** | **Hypocalcemia** | **Decreased Weight** | **Decreased Appetite** | **Cachexia** | **Hyperglycaemia** |
| Casadei-Gardini et al。(2023) | 379/1343 (28.2%) vs 52/864 (6.0%) | NR | NR | NR | 427/1343 ( 31.8%) vs 171/864 (19.8%) | NR | NR | 13/1343 (0.9%) vs 3/864 (0.4%) | NR | NR | NR | 73/1343 ( 5.4%) vs 16/864 (1.8%) | NR | NR |
| Haddad et al. (2017) | NR | NR | NR | NR | NR | NR | NR | NR | NR | NR | NR | NR | NR | NR |
| Kiyota et al. (2017) | NR | NR | NR | 183/379 (48.3%) vs 17/204 (8.3%) | 188/379 (49.6%) vs 22/204 (10.8%) | NR | NR | NR | NR | NR | 34/379 (9.0%) vs 0/204 (0%) | 18/379 (4.7%) vs 0/204 (0%) | NR | NR |
| Kudo et al. (2018) | 78/476 (16.4%) vs 8/475 (1.7%) | NR | NR | 147/476 (30.9%) vs 106/475 (22.3%) | 162/476 (34.0%) vs 127/475 (26.7%) | NR | NR | 0/476 (0%) vs 0/475 (0%) | NR | NR | 36/476 (7.6%) vs 14/475 (2.9%) | 22/476 (4.6%) vs 6/475 (1.3%) | NR | NR |
| Matsubara et al. (2024) | 88/241 (36.5%) vs 17/242 (7.0%) | NR | NR | NR | 35/241 (14.5%) vs 14/242 (5.8%) | 1/241 (0.4%) vs 0/242 (0%) | NR | 1/241 (0.4%) vs 0/242 (0%) | NR | NR | NR | 4/241 (1.7%) vs 0/242 (0%) | 1/241 (0.4%) vs 0/242 (0%) | NR |
| Motzer et al. (2015) | 19/52 (36.5%) vs 1/50 (2.0%) | 2/52 (3.8%) vs 1/50 (2.0%) | NR | 25/52 (48.1%) vs 4/50 (8.0%) | 30/52 (57.7%) vs 9/50 (18.0%) | NR | 3/52 (5.8%) vs 11/50 (22.0%) | 1/52 (1.9%) vs 0/50 (0%) | 0/52 (0%) vs 0/50 (0%) | NR | 3/52 (5.8%) vs 0/50 (0%) | 2/52 (3.8%) vs 0/50 (0%) | NR | 0/52 (0%) vs 5/50 (10.0%) |
| Nair et al. (2021) | 102/476 (21%) vs 16/475 (3%) | NR | NR | 149/476 (31%) vs 106/475 (22%) | 163/476 (34%) vs 130/475 (27%) | NR | NR | 0/476 (0%) vs 0/475 (0%) | NR | NR | 40/476 (8%) vs 16/475 (3%) | 25/476 (5%) vs 6/475 (1%) | NR | NR |
| Yang et al. (2024) | 128/309 (41.4%) vs 31/312 (9.9%) | NR | 21/309 (6.8%) vs 9/312 (2.9%) | NR | NR | NR | NR | 0/309 (0%) vs 0/312 (0%) | NR | 3/309 (1.0%) vs 1/312 (0.3%) | NR | NR | NR | NR |
| Zheng et al. (2021) | NR | NR | 30/103 (29.1%) vs 8/48 (16.7%) | 49/103 (47.6%) vs 5/48 (10.4%) | 21/103 (20.4%) vs 3/48 (6.3%) | NR | NR | NR | NR | 4/103 (3.9%) vs 1/48 (2.1%) | 3/103 (2.9%) vs 0/48 (0%) | 2/103 (1.9%) vs 0/48 (0%) | NR | NR |

NR: Not Reported.
