# Supplementary material for: Systematic meta-analysis of the toxicities and side effects of the targeted drug lenvatinib
Source: Ann Med. 2025 Dec 24;58(1):2598935. doi: 10.1080/07853890.2025.2598935 (PMC12777875; doi:10.1080/07853890.2025.2598935)
Supplement: Supplemental Material [file IANN_A_2598935_SM0031.zip › suppl_data/Supplementary Table 14.docx]

**Supplementary Table 14. Meta-analysis of the Toxicity of Lenvatinib to the Musculoskeletal System**

| **Author (year)** | **Any Grade** | | | | | | **Grade ≥ 3** | | | | | |
| --- | --- | --- | --- | --- | --- | --- | --- | --- | --- | --- | --- | --- |
|  | **Musculoskeletal Chest Pain**  **n/N (%)** | **Arthralgia**  **n/N (%)** | **Myalgia**  **n/N (%)** | **Back Pain**  **n/N (%)** | **Musculoskeletal Pain**  **n/N (%)** | **Pain in Extremity**  **n/N (%)** | **Musculoskeletal Chest Pain**  **n/N (%)** | **Arthralgia**  **n/N (%)** | **Myalgia**  **n/N (%)** | **Back pain**  **n/N (%)** | **Musculoskeletal Pain**  **n/N (%)** | **Pain in Extremity**  **n/N (%)** |
| Casadei-Gardini et al。(2023) | NR | NR | NR | NR | NR | NR | NR | NR | NR | NR | NR | NR |
| Haddad et al. (2017) | NR | NR | NR | NR | NR | NR | NR | NR | NR | NR | NR | NR |
| Kiyota et al. (2017) | NR | 62/379 (16.4%) vs 1/204 (0.5%) | NR | NR | NR | NR | NR | 0/379 (0%) vs 0/204 (0%) | NR | NR | NR | NR |
| Kudo et al. (2018) | NR | NR | NR | NR | NR | NR | NR | NR | NR | NR | NR | NR |
| Matsubara et al. (2024) | NR | NR | NR | NR | NR | NR | NR | NR | NR | NR | NR | NR |
| Motzer et al. (2015) | 6/52 (11.5%) vs 2/50 (4.0 %) | 13/52 (25.0%) vs 7/50 (14.0%) | 7/52 (13.5%)vs 1/50 (2.0%) | 11/52 (21.2%) vs 7/50 (14.0%) | 7/52 (13.5%) vs 1/50 (2.0%) | 6/52 (11.5%) vs 3/50 (6%) | 1/52 (1.9%) vs 0/50 (0 %) | 0/52 (0%) vs 0/50 (0%) | 1/52 (1.9%)vs 0/50 (0%) | 0/52 (0%) vs 0/50 (0%) | 1/52 (1.9%) vs 0/50 (0%) | 1/52 (1.9%) vs 0/50 (0%) |
| Nair et al. (2021) | 149/476 (31%) vs 95/475 (20%) | | | NR | NR | NR | 6/476 (1%) vs 11/475 (2%) | | | NR | NR | NR |
| Yang et al. (2024) | NR | NR | NR | NR | NR | NR | NR | NR | NR | NR | NR | NR |
| Zheng et al. (2021) | NR | NR | NR | NR | NR | NR | NR | NR | NR | NR | NR | NR |

NR: Not Reported.
