# Supplementary material for: Systematic meta-analysis of the toxicities and side effects of the targeted drug lenvatinib
Source: Ann Med. 2025 Dec 24;58(1):2598935. doi: 10.1080/07853890.2025.2598935 (PMC12777875; doi:10.1080/07853890.2025.2598935)
Supplement: Supplemental Material [file IANN_A_2598935_SM0031.zip › suppl_data/Supplementary Table 15.docx]

**Supplementary Table 15. Meta-analysis of Other Severe Toxicities of Lenvatinib**

| **Author (year)** | **Any Grade** | | | **Grade ≥ 3** | | |
| --- | --- | --- | --- | --- | --- | --- |
|  | **Death**  **n/N (%)** | **Sepsis**  **n/N (%)** | **Blood Lactate Dehydrogenase Increased**  **n/N (%)** | **Death**  **n/N (%)** | **Sepsis**  **n/N (%)** | **Blood Lactate Dehydrogenase Increased**  **n/N (%)** |
| Casadei-Gardini et al. (2023) | NR | NR | NR | NR | NR | NR |
| Haddad et al. (2017) | NR | NR | NR | NR | NR | NR |
| Kiyota et al. (2017) | NR | NR | NR | NR | NR | NR |
| Kudo et al. (2018) | NR | NR | NR | NR | NR | NR |
| Matsubara et al. (2024) | 1/241 (0.4%) vs 0/242 (0%) | 1/241 (0.4%) vs 0/242 (0%) | NR | 1/241 (0.4%) vs 0/242 (0%) | 1/241 (0.4%) vs 0/242 (0%) | NR |
| Motzer et al. (2015) | NR | NR | NR | NR | NR | NR |
| Nair et al. (2021) | NR | NR | NR | NR | NR | NR |
| Yang et al. (2024) | 16/309 (5.2%) vs 6/312 (1.9%) | NR | NR | NR | NR | NR |
| Zheng et al. (2021) | NR | NR | 26/103 (26.2%) vs 2/48 (4.2%) | NR | NR | 0/103 (0%) vs 0/48 (0%) |

NR: Not Reported.
