# Supplementary material for: Systematic meta-analysis of the toxicities and side effects of the targeted drug lenvatinib
Source: Ann Med. 2025 Dec 24;58(1):2598935. doi: 10.1080/07853890.2025.2598935 (PMC12777875; doi:10.1080/07853890.2025.2598935)
Supplement: Supplemental Material [file IANN_A_2598935_SM0031.zip › suppl_data/Supplementary Table 2.docx]

**Supplementary Table 2. Eligibility Criteria of Included Studies**

|  | **Inclusion criteria** | **Exclusion criteria** |
| --- | --- | --- |
| **Participants** | The study must include patients treated with the targeted drug lenvatinib. | There are no specific exclusions, but the study must involve human patients. |
| **Intervention** | The intervention is lenvatinib treatment, and the study needs to focus on its toxic and side effects. | Studies that do not utilize the targeted drug lenvatinib for treatment, or that only involve short-term treatment, will be excluded. |
| **Outcomes** | The study should provide detailed data regarding the toxic and side effects of lenvatinib, including but not limited to the incidence rate of adverse reactions, severity, and management approaches. | Studies that do not utilize the targeted drug lenvatinib for treatment, or that only involve short-term treatment, will be excluded. |
| **Study**  **design/Settings** | • The study design should be a cohort study, a randomized controlled trial (RCT), or a long-term observational study.  • The study should include a sufficient sample size to support the statistical significance of the conclusions drawn. | • Non-clinical studies, including animal experiments, in vitro studies, or cell culture experiments, will be excluded.  • Studies that have been published previously or are based on the same dataset but lack new analyses or conclusions will be excluded.  • Studies with poor methodological quality and high risk of bias, such as those that do not follow standard research design, data collection, or analysis methods, will be excluded. |
