# Supplementary material for: Systematic meta-analysis of the toxicities and side effects of the targeted drug lenvatinib
Source: Ann Med. 2025 Dec 24;58(1):2598935. doi: 10.1080/07853890.2025.2598935 (PMC12777875; doi:10.1080/07853890.2025.2598935)
Supplement: Supplemental Material [file IANN_A_2598935_SM0031.zip › suppl_data/Supplementary Table 3.docx]

**Supplementary Table 3. Abstracts and Newly Published Studies**

| **Title** | **Author (Year)** | **Journals** | **Study Design** | **Groups** | **Sample Size** | **Principal Findings (Toxic and side effects)** |
| --- | --- | --- | --- | --- | --- | --- |
| [Pembrolizumab with or Without Lenvatinib as First-line Therapy for Patients with Advanced Urothelial Carcinoma (LEAP-011): A Phase 3, Randomized, Double-Blind Trial](https://pubmed.ncbi.nlm.nih.gov/37778952/) | Matsubara, et al. (2024) | Eur Urol | A Phase 3, Randomized, Double-Blind Trial | Lenvatinib plus pembrolizumab vs Placebo plus pembrolizumab | 241 vs 242 | **1. High Incidence of Any AEs:** 87.6% of patients experienced AEs compared to 69.0% in the control group.  **2. Hypertension:** Notably, 34.9% of patients developed hypertension, significantly higher than the 7.0% in the control group.  **3. Skin/Subcutaneous Tissue Reactions:**  • Rash occurred in 10.0% vs 5.8%.  • Pruritus was reported in 11.2% vs 14.5%.  **4. Respiratory, Thoracic, and Mediastinal Issues:** Dysphonia was observed in 10.8% of patients, a much higher rate compared to 0.4% in the control group.  **5. Gastrointestinal Disturbances:**  • Diarrhea affected 20.7% vs 10.3%.  • Nausea was experienced by 11.6% vs 8.3%.  **6. Renal/Urinary Problems:**  • Proteinuria was common, affecting 37.8% vs 18.6%.  • Renal failure occurred in 0.8% vs 1.2%, although the difference is not substantial.  **7. Liver Dysfunction:** Increased lipase levels were found in 12.0% vs 7.0%.  **8. General Symptoms:**  • Fatigue was reported in 14.5% vs 12.0%.  • Asthenia affected 12.0% vs 5.0%.  **9. Endocrine Disorders:** Hypothyroidism was prevalent, affecting 36.5% vs 7.0%.  **10. Metabolism/Nutrition Issues:**  • Decreased appetite was observed in 14.5% vs 5.8%.  • Cachexia was rare, affecting 0.4% vs 0%.  **11. Respiratory System Complications:** Pneumonitis occurred in 3.3% vs 2.5%.  **12. Cardiac Problems:** Cardiac failure was reported in 1.2% vs 0%.  **13. Other Serious Adverse Events:**  • Death was reported in 0.4% vs 0%.  • Sepsis affected 0.4% vs 0%, indicating a low but notable risk. |
| Pembrolizumab With or Without Lenvatinib for First-Line Metastatic NSCLC With Programmed Cell Death-Ligand 1 Tumor Proportion Score of at least 1% (LEAP-007): A Randomized, Double-Blind, Phase 3 Trial | Yang, et al. (2024) | J Thorac Oncol | A Randomized, Double-Blind, Phase 3 Trial | Lenvatinib plus pembrolizumab vs Placebo plus pembrolizumab | 309 vs 312 | **1. High Incidence of TRAEs:**  • 91.3% of patients in the lenvatinib group experienced TRAEs of any grade, compared to 70.2% in the control group.  • Treatment was discontinued due to TRAEs in 46.3% of patients, versus 17.3% in the control group.  • 27.5% of patients stopped treatment due to TRAEs, compared to 9.0% in the control group.  **2. Common Specific Toxicities and Side Effects:**  **• Cardiovascular system:** Hypertension occurred in 40.8% (lenvatinib group) vs. 14.7% (control group).  **• Skin/subcutaneous tissue:** Palmar-plantar erythrodysesthesia syndrome occurred in 51.9% (specific subgroup, lenvatinib) vs. 73.6% (specific subgroup, control); alopecia occurred in 6.2% (lenvatinib group) vs. 36.8% (control subgroup).  **• Nervous system:** Posterior reversible encephalopathy syndrome was less common, with an incidence of 1.0% (lenvatinib group) vs. 0% (control group).  **• Hematological system:** Bleeding occurred in 24.9% (lenvatinib group) vs. 17.0% (control group).  **• Gastrointestinal tract:** The incidence of fistula formation and gastrointestinal perforation was 1.6% and 1.3%, respectively, in the lenvatinib group, compared to 0% and 0.3% in the control group.  **• Renal/urinary system:** Proteinuria occurred in 32.0% (lenvatinib group) vs. 10.9% (control group); renal failure occurred in 3.9% (lenvatinib group) vs. 2.6% (control group).  **• Liver:** Hepatic toxicity occurred in 28.2% (lenvatinib group) vs. 16.7% (control group).  **• Endocrine system:** Hypothyroidism occurred in 41.4% (lenvatinib group) vs. 9.9% (control group); hypocalcemia occurred in 6.8% (lenvatinib group) vs. 2.9% (control group).  **• Heart:** Heart failure occurred in 4.5% (lenvatinib group) vs. 2.2% (control group); the incidence of arterial thromboembolic events was similar, at 3.6% and 3.5%, respectively, for the lenvatinib group and control group; QT prolongation was less common, with an incidence of 1.9% (lenvatinib group) vs. 0.3% (control group).  **3. Severe Toxicities and Side Effects:**  • 5.2% of patients in the lenvatinib group died, compared to 1.9% in the control group. |
