# Supplementary material for: Systematic meta-analysis of the toxicities and side effects of the targeted drug lenvatinib
Source: Ann Med. 2025 Dec 24;58(1):2598935. doi: 10.1080/07853890.2025.2598935 (PMC12777875; doi:10.1080/07853890.2025.2598935)
Supplement: Supplemental Material [file IANN_A_2598935_SM0031.zip › suppl_data/Supplementary Table 4.docx]

**Supplementary Table 4. Assessment of the Risk of Bias using the Cochrane Tools on RCT Studies**

| **Author (Year)** | **Selection bias** | | **Performance bias** | **Detection bias** | **Attrition bias** | **Reporting bias** | **Other bias** |
| --- | --- | --- | --- | --- | --- | --- | --- |
|  | **Random sequence generation** | **Allocation concealment** | **Blinding of participants, personnel** | **Blinding of outcome assessment** | **Incomplete outcome data addressed** | **Selective reporting** |  |
| Haddad et al. (2017) | Low risk | Unclear risk | Low risk | Low risk | Unclear risk | Low risk | Unclear risk |
|  | The study mentioned that "eligible patients were stratified by geographic region, age group, and prior VEGF-targeted therapy and randomly assigned in a 2:1 ratio to receive oral lenvatinib or placebo." Although the specific method used for random sequence generation (such as random number tables, computer-generated, etc.) was not described in detail, the mention of random assignment allows for the assumption that an appropriate randomization method was utilized. | The study did not explicitly describe the method of allocation concealment (such as the use of opaque envelopes, central randomization, etc.). Allocation concealment is a critical step to ensure that study participants and researchers are unaware of the treatment assignments prior to allocation. Due to the missing information, it is not possible to assess whether allocation concealment was adequately implemented. | The study employed a double-blind design, meaning that neither the participants nor the researchers were aware of whether the patients were receiving lenvatinib or placebo. This helps to reduce bias arising from knowledge of the treatment assignment. | Due to the double-blind nature of the study, the outcome assessors (if they are independent and unaware of the treatment assignments) are also unlikely to be influenced by the treatment allocation. Although the blinding status of the outcome assessors is not explicitly mentioned, a double-blind design typically implies that the outcome assessment is also blinded. | The study did not provide detailed information on how incomplete outcome data (such as loss to follow-up, withdrawal, etc.) were handled. Appropriate methods for dealing with incomplete data (such as intention-to-treat analysis, per-protocol set analysis, etc.) are crucial for avoiding bias. Due to the missing information, it is not possible to assess the risk of bias related to the handling of incomplete data. | The study provided detailed information on the study design, inclusion criteria, treatment regimen, endpoints, and assessment methods, and mentioned the primary report (ClinicalTrials.gov identifier). This indicates that the study results were reported comprehensively and transparently, with no indication of selective reporting. | Although the study adhered to the Declaration of Helsinki and local laws and obtained ethical committee approval, specific factors that could introduce other biases (such as conflicts of interest, baseline imbalances, etc.) were not mentioned. Since this information was not explicitly provided, the possibility of other biases cannot be completely ruled out. |
| Kiyota et al. (2017) | Low risk | Low risk | High risk | Low risk | Unclear risk | Low risk | Unclear risk |
|  | The study mentions that patients were randomized in a specific ratio (2:1) to receive either lenvatinib or placebo treatment, but does not specifically detail the method used for random sequence generation. However, given that the study adhered to standard clinical trial design protocols and obtained approval from an ethics committee, typically such studies employ appropriate randomization methods (such as computer-generated random numbers or randomization tables). Therefore, in the absence of contrary evidence, it is assumed that the method for random sequence generation was appropriate, and is rated as having a low risk of bias. | Although the study does not explicitly describe the specific method used for allocation concealment, randomized clinical trials typically employ central randomization, sealed envelopes, or other methods to ensure the concealment of allocation schemes. Given that the study adhered to ethical and regulatory requirements and obtained approval, it is reasonable to infer that allocation concealment was adequately addressed. Therefore, it is assessed as having a low risk of bias. | The study involves a comparison between lenvatinib and placebo, but blinding in pharmacological treatments, especially anticancer drugs, can be challenging due to potential differences in color, shape, taste, and other characteristics of the medications. Despite efforts made by the study to ensure blinding, participants and researchers may still be able to guess the treatment allocation through certain cues, such as side effects or improvements in condition, thereby introducing detection bias. Consequently, it is assessed as having a high risk of bias. | The primary endpoint of the study is progression-free survival (PFS) determined by independent radiological review, which helps to reduce subjectivity in outcome assessment. Therefore, it is assessed as having a low risk of bias. | The study did not provide a detailed description of how incomplete outcome data (such as loss to follow-up, withdrawal, etc.) were handled. Although mention was made of ethical committee approval and standard procedures for clinical trials, these pieces of information are insufficient for a comprehensive assessment of the bias risk associated with handling incomplete data. The absence of a clear handling plan may lead to data missingness or bias, thereby affecting the reliability of the results. | The study provided detailed information on the study design, inclusion criteria, treatment regimen, endpoints, and assessment methods, and mentioned the primary report (ClinicalTrials.gov identifier). This indicates that the study results were reported comprehensively and transparently, with no evidence of selective reporting. Therefore, it is assessed as having a low risk of bias. | Although the study adhered to ethical and legal requirements and obtained approval, specific factors such as conflicts of interest and baseline imbalances were not mentioned, which may introduce additional biases. Furthermore, patients could be categorized into multiple groups (e.g., "no RAI uptake," "disease progression despite RAI avidity," and "extensive RAI exposure" groups), which may obscure differences between the groups and thereby introduce potential bias. |
| Kudo et al. (2018) | Low risk | Low risk | High risk | High risk | Unclear risk | Low risk | Unclear risk |
|  | The study indicates that "the randomization sequence was generated by an independent statistician from the system vendor, and the researchers directly obtained the randomization assignment from the system." This suggests that the generation of the randomization sequence was independent and not influenced by the researchers, thus posing a low risk of bias. | The study utilized an "interactive voice-web response system" for allocation, which served as the method for allocation concealment. This implies that neither the researchers nor the participants were aware of the upcoming treatment group assignment prior to allocation, thereby minimizing selection bias. | The study clearly states, "Due to the open-label nature of the study, blinding of treatment allocation was not performed for patients or researchers." This implies that both participants and researchers were aware of the treatment allocation, which may lead to detection bias. | Although the study mentions that tumor assessments were conducted by local researchers according to mRECIST, it does not explicitly state whether these assessments were performed by blinded personnel unaware of the treatment allocation. Given the open-label nature of the study and the absence of mention regarding blinding of the assessors, there is a risk of outcome assessment bias. However, the information provided is insufficient to determine the exact extent of the bias risk. | The study did not provide detailed information on how incomplete outcome data (such as loss to follow-up, withdrawal, etc.) were handled. This is one of the key factors in assessing the risk of bias, as inappropriate handling can lead to outcome bias. | The study detailed the primary and secondary endpoints and mentioned a comprehensive assessment of the outcomes. Additionally, the study noted that "the research was overseen by an independent data monitoring committee" and that "the corresponding author had full access to all the data in the study and had final responsibility for the decision to submit for publication." These measures help to reduce the risk of selective reporting, as the integrity and accuracy of the data are supervised. | The study design itself (such as an open-label study) may introduce other types of bias, such as performance bias and detection bias. Furthermore, although the study mentions inclusion and exclusion criteria, it does not provide detailed information on whether these criteria were adequately adhered to. Therefore, there is a risk of other potential biases. |
| Matsubara et al. (2024) | Low risk | Unclear risk | Low risk | Low risk | Unclear risk | Low risk | Unclear risk |
|  | The study mentions that "patients were randomly assigned using an interactive response technology system," which indicates that the random sequence was generated through some technological means, and typically, such technology can ensure randomness. Therefore, it is considered that there is a low risk of bias in terms of random sequence generation. | Although the study mentions random allocation, it does not specify the method used for allocation concealment (such as sealed envelopes). | The study indicates that "blinding to treatment assignment was maintained at all study sites," and for patients ineligible for platinum-based therapy, the investigators were blinded to the PD-L1 CPS results (partial blinding). This suggests that participants and personnel were largely blinded to the treatment assignment, and therefore, it is considered that there is a low risk of bias. | Since the study maintained blinding to treatment assignment and no potential unblinding situations were mentioned during the outcome assessment process, it is reasonable to infer that the outcome assessment was also blinded, and there is a low risk of bias. | The study does not specify how incomplete outcome data (such as loss to follow-up, withdrawal, etc.) were handled. | The study mentions the primary study outcomes (such as PFS, OS, etc.) and includes an assessment of safety. Despite the premature termination of the trial, there does not appear to be concealment or selective reporting of unfavorable results for lenvatinib plus pembrolizumab. However, this assessment is subject to some uncertainty due to the lack of a complete dataset and analysis plan provided. | Other potential biases (such as conflicts of interest, flaws in study design, etc.) are not explicitly mentioned in the study. |
| Motzer et al. (2015) | Low risk | Low risk | High risk | Unclear risk | Low risk | Low risk | Unclear risk |
|  | The study mentions that "an external interactive voice response system vendor conducted central randomization using Pocock and Simon's dynamic allocation procedure." This indicates that the random sequence was generated through a reliable method, thus the risk of bias is low. | Although the study did not directly specify the exact method of allocation concealment, central randomization generally implies that allocation is concealed as the allocation information is not known to researchers or participants at the time of patient enrollment. Therefore, it is reasonable to infer that allocation concealment was adequate, and the risk of bias is low. | The study clearly indicates that it is "open-label," meaning that both patients and researchers are aware of the treatment allocation. Consequently, blinding of participants and personnel is not possible, posing a high risk of bias. | Although outcome assessment was based on investigator assessment and RECIST version 1.1, the article did not specify whether the assessors were blinded to the treatment allocation. | The study mentions that patients who were lost to follow-up or still alive at the data cutoff date were censored, with the date being their last known date of survival. This indicates that the researchers appropriately handled incomplete data. | The study provides relatively detailed descriptions of methods and results, with no apparent indications of selective reporting. | Although the study describes the inclusion and exclusion criteria, ethical review, etc., it does not explicitly mention other potential biases such as conflicts of interest or flaws in the study design. |
| Nair et al. (2021) | Low risk | Low risk | High risk | High risk | Unclear risk | Unclear risk | Low risk |
|  | The study states that "patients were randomly assigned in a 1:1 ratio," although the specific randomization method (such as a random number table, computer generation, etc.) was not detailed. However, in the absence of contrary evidence, it is generally considered that mentioning random assignment implies the use of an appropriate method for generating the random sequence. | Although the specific method for allocation concealment (such as central randomization, sealed envelopes, etc.) was not directly mentioned, the study is international in scope and describes a detailed stratified randomization process, which typically suggests a relatively strict allocation control mechanism. Therefore, it is reasonable to infer that allocation concealment may have been appropriate, unless there is contrary evidence. | The study was open-label, meaning that both participants and personnel were aware of the specific drug being administered. This directly violates the principle of blinding and thus poses a high risk of bias. | Although it is not directly stated whether the outcome assessors were aware of the treatment allocation, due to the open-label nature of the study, it is generally inferable that the outcome assessors, especially if they were clinical investigators or personnel involved in the treatment, likely also knew the treatment allocation, thereby potentially affecting the objectivity of the assessments. | The study did not provide specific information on how incomplete outcome data (such as loss to follow-up, withdrawal, etc.) were handled. | The study described primary and secondary efficacy endpoints but did not mention whether there was selective reporting of results. | The study described inclusion and exclusion criteria, ethical review, and other aspects, which typically help to reduce bias. |
| Yang et al. (2024) | Low risk | Unclear risk | Low risk | Low risk | Low risk | Low risk | Low risk |
|  | In the study description, patients were mentioned to have been randomly assigned in a 1:1 ratio to receive oral lenvatinib or placebo. Although the specific method used for generating the random sequence (such as a random number table or computer-generated randomization) was not detailed, it is generally assumed to be at low risk of bias when described as "random" and no obvious violations of randomization principles are mentioned. | The specific method for allocation concealment (such as sealed envelopes or a central randomization system) was not explicitly mentioned in the study description. Allocation concealment is a crucial step in ensuring that the random sequence is not compromised during the allocation process. Due to the lack of specific information, the assessment is therefore of uncertain risk of bias. | The study employed a double-blind design, where neither the patients nor the investigators were aware of the treatment allocation. This design helps to reduce bias arising from knowledge of the treatment allocation, and therefore, it is assessed as being at low risk of bias. | The study mentioned that response assessment was conducted using RECIST version 1.1 by a blinded independent central review (BICR). This implies that the assessment of outcomes was performed by an independent third party who was unaware of the treatment allocation, which helps to reduce detection bias. Therefore, it is assessed as being at low risk of bias. | The study explicitly mentioned the censoring situations in the OS and PFS analyses, including the handling of patients without recorded death or progressive disease (PD) prior to the data cutoff date. This indicates that the researchers took reasonable measures in dealing with incomplete outcome data, and therefore, it is assessed as being at low risk. | The study described multiple primary and secondary endpoints, including overall survival, progression-free survival, objective response rate, safety, and others, and did not mention any instances of selective reporting of results. Therefore, it is assessed as being at low risk of bias. | Despite the overall rigorous design of the study, it mentioned that the open-label design could introduce observer bias or performance bias. However, after considering these potential impacts, the researchers assessed other biases as low risk. Additionally, the study adhered to ethical requirements such as those outlined in the Helsinki Declaration and obtained approval from the relevant ethics committee. These measures help to reduce other forms of bias. Therefore, upon comprehensive consideration, it is assessed as being at low risk of bias, but attention should be given to the potential impacts of the open-label design. |
| Zheng et al. (2021) | Low risk | Low risk | Low risk | Low risk | Unclear risk | Low risk | Low risk |
|  | The study describes the randomization of patients in a 2:1 ratio, although the specific method used for generating the random sequence (such as random number tables, computer randomization programs, etc.) is not elaborated. However, typically, multi-center, Phase III studies employ reliable randomization methods to ensure the unpredictability of the random sequence. In the absence of contrary evidence, it can be presumed to pose a low risk of bias. | Although the study did not directly state the method of allocation concealment, the design of a multicenter, randomized, double-blind, placebo-controlled study generally implies that allocation concealment was properly addressed to prevent researchers and participants from foreseeing the allocation. In the absence of specific information, based on the standards of study design, it can be presumed to pose a low risk of bias. | The study clearly indicates a double-blind design, where neither participants nor personnel are aware of whether the patients are receiving lenvatinib or a placebo. This helps to reduce bias arising from knowledge of the treatment allocation. | Tumor assessment was conducted by an Independent Imaging Review (IIR), which means that the assessors were unaware of the specific treatment received by the patients. This blinded assessment helps to reduce detection bias. | Although the study described the protocols for toxicity management and dose adjustments, it did not elaborate on how data from patients who withdrew from the study due to adverse events or other reasons were handled. The incomplete handling of these data may affect the reliability of the results. | The study provides a detailed description of the design, conduct, and methods for analyzing results, including primary endpoints, secondary endpoints, and statistical analysis methods, and is therefore deemed to be at low risk. | The study adhered to ethical requirements such as the Helsinki Declaration and obtained approval from the relevant ethics committee, which helps to reduce ethical biases. However, it is noteworthy that patients receiving placebo and experiencing confirmed disease progression were eligible to cross over to receive optional open-label lenvatinib treatment. This crossover treatment may affect the assessment of the long-term efficacy and safety of lenvatinib. However, at this stage of evaluation, it is not considered a major source of bias, as it is a treatment option after disease progression. The primary focus is on biases during the randomization phase and blinded assessment. |
