# Supplementary material for: Systematic meta-analysis of the toxicities and side effects of the targeted drug lenvatinib
Source: Ann Med. 2025 Dec 24;58(1):2598935. doi: 10.1080/07853890.2025.2598935 (PMC12777875; doi:10.1080/07853890.2025.2598935)
Supplement: Supplemental Material [file IANN_A_2598935_SM0031.zip › suppl_data/Supplementary Table 5.docx]

**Supplementary Table 5. Assessment of the Risk of Bias using the Newcastle-Ottawa Scale in non-RCT Studies**

| **Author (Year)** | **Representativeness of the exposed cohort (perfect score=4)** | **Selection of the non-**exposed cohort **(perfect score=2)** | **Ascertainment of exposure (perfect score=3)** | **Demonstration that outcome of interest was not present at baseline (perfect score=2)** | **Comparability of cohorts based on the design or analysis (perfect score=2)** | **Assessment of outcomes (perfect score=3)** | **Was follow-up long enough for outcomes to occur (perfect score=1)** | **Adequacy of follow-up of cohorts (perfect score=1)** | **Total number of stars (risk of bias) (perfect score=22)** |
| --- | --- | --- | --- | --- | --- | --- | --- | --- | --- |
| Casadei-Gardini et al. (2023) | 3 | 2 | 3 | 2 | 2 | 3 | 1 | 1 | 19 (low) |
|  | The study included Western and Eastern populations from 42 centers across five countries, which to some extent increased the representativeness of the cohort. However, the selection criteria for these centers and whether they encompassed all possible patient groups were not clearly specified, thus making it impossible to determine its complete representativeness. | The study explicitly mentioned the patient population treated with lenvatinib and, following the approval of atezolizumab plus bevacizumab, the treatment choice was determined by the attending physician. This implies the existence of an unexposed population (i.e., those who did not receive atezolizumab plus bevacizumab), and the selection was based on clinical judgment. Although the specific selection process for the unexposed population was not described in detail, selection based on clinical judgment is common in practical situations. | The study clearly specified the administration routes and dosages of lenvatinib and atezolizumab plus bevacizumab, ensuring accurate determination of exposure. Additionally, the management of treatment interruptions and dose reductions was also mentioned, further enhancing the reliability of exposure determination. | Although it was not directly mentioned whether the outcomes of interest (such as overall survival or progression-free survival) were absent at baseline, the study design included retrospective data collection and adhered to international guidelines for diagnosis and inclusion criteria. This implies that the patient population was relatively homogeneous at baseline and no significant prognostic differences were noted. Therefore, it can be indirectly inferred that there were no significant differences in the outcomes of interest at baseline. | The study utilized Inclination Propensity Treatment Weighting (IPTW) to adjust for differences in baseline characteristics, ensuring comparability between the two groups. This method is effective in addressing selection bias in non-randomized controlled trials. Therefore, it can be considered that the cohorts are comparable based on design and analysis. | The study employed various statistical methods to evaluate the outcomes, including Kaplan-Meier curves, log-rank tests, and Cox regression. These methods are standard in survival analysis and capable of accurately assessing the relationship between treatment and the outcomes of interest. Additionally, the impact of second-line treatment was also considered, enhancing the reliability of the results. | The study mentioned that patients underwent follow-up every 2-3 months, with the follow-up continuing until radiological disease progression or when the imaging became clinically irrelevant. Although the total duration of follow-up was not specifically stated, based on this follow-up frequency and condition, it can be considered that the follow-up duration was sufficiently long to assess the outcomes of interest. | The study explicitly mentioned the frequency and conditions of follow-up and employed various statistical methods to process the follow-up data. This indicates that the follow-up of the cohorts was adequate and capable of accurately assessing the outcomes of interest. | Based on the aforementioned ratings, the risk of bias in this study is relatively low. Although there are minor uncertainties regarding the representativeness of the exposed cohort and some specific details, overall, the study design and methodology are reasonable and capable of controlling bias risks to a certain extent. |
