# Supplementary material for: Systematic meta-analysis of the toxicities and side effects of the targeted drug lenvatinib
Source: Ann Med. 2025 Dec 24;58(1):2598935. doi: 10.1080/07853890.2025.2598935 (PMC12777875; doi:10.1080/07853890.2025.2598935)
Supplement: Supplemental Material [file IANN_A_2598935_SM0031.zip › suppl_data/Supplementary Table 6.docx]

**Supplementary Table 6. Comprehensive Assessment Data of the Impact of Lenvatinib's Toxicities and Side Effects on Patient AEs**

| **Author (year)** | **Any Grade** | | | | | | | | | | | **Grade ≥3** | | |
| --- | --- | --- | --- | --- | --- | --- | --- | --- | --- | --- | --- | --- | --- | --- |
|  | **Any AEs n/N (%)** | | | | | | | | | | | **AEs n/N (%)** | | |
|  | **Any TRAEs** | **Any TRAEs led to treatment interruption** | **Any TRAEs led to discontinuation** | **Total TEAEs** | **Total treatment-related TEAEs** | **Serious TEAEs** | **Serious treatment-related TEAEs** | **Fatal TEAEs** | **Dose reduction due to TEAEs** | **Dose interruption due to TEAEs** | **TEAEs-related treatment discontinuation** | **Any TRAEs** | **Total TEAEs** | **Total treatment-related TEAEs** |
| Casadei-Gardini et al. (2023) | 1140/1343 (84.9%) vs 603/864 (69.8%) | | | | | | | | | | | 921/1343 (68.7%) vs 421/864 (48.8%) | | |
| Haddad et al. (2017) | NR | NR | NR | NR | NR | NR | NR | NR | NR | NR | NR | NR | NR | NR |
| Kiyota et al. (2017) | NR | NR | NR | NR | 367/379 (96.8%) vs 112/204 (54.9%) | NR | NR | NR | NR | NR | NR | NR | NR | 282/379 (74.4%) vs 18/204 (8.8%) |
| Kudo et al. (2018) | NR | NR | NR | 470/476 (98.7%) vs 472/475 (99.4%) | 447/476 (93.9%) vs 452/475 (95.2%) | 205/476 (43.1%) vs 144/475 (30.3%) | 84/476 (17.6%) vs 48/475 (10.1%) | NR | NR | NR | NR | NR | 357/476 (75.0%) vs 316/475 (66.5%) | 270/476 (56.7%) vs 231/475 (48.6%) |
| Matsubara et al. (2024) | 211/241 (87.6%) vs 167/242 (69.0%) | | | | | | | | | | | 123/241 (51.0%) vs 66/242 (27.3%) | | |
| Motzer et al. (2015) | NR | NR | NR | 49/52 (94.2%) vs 48/50 (96%) | NR | NR | NR | NR | NR | NR | NR | NR | 41/52 (78.8%) vs 25/50 (50%) | NR |
| Nair et al. (2021) | NR | NR | NR | NR | NR | NR | NR | NR | NR | NR | NR | NR | NR | NR |
| Yang et al. (2024) | 282/309 (91.3%) vs 219/312 (70.2%) | 143/309 (46.3%) vs 54/312 (17.3%) | 85/309 (27.5%) vs 28/312 (9.0%) | NR | NR | NR | NR | NR | NR | NR | NR | 179/309 (57.9%) vs 76/312 (24.4%) | NR | NR |
| Zheng et al. (2021) | NR | NR | NR | 103/103 (100%) vs 47/48 (97.9%) | 103/103 (100%) vs 34/48 (70.8%) | 40/103 (38.8%) vs 16/48 (33.3%) | NR | 10/103 (9.7%) vs 3/48 (6.3%) | 83/103 (80.6%) vs 3/48 (6.3%) | 69/103 (67.0%) vs 4/48 (8.3%) | 9/103 (8.7%) vs 3/48 (6.3%) | NR | 90/103 (87.4%) vs 22/48 (45.8%) | 88/103 (85.4%) vs 8/48 (16.7%) |

NR: Not Reported.
