# Supplementary material for: Systematic meta-analysis of the toxicities and side effects of the targeted drug lenvatinib
Source: Ann Med. 2025 Dec 24;58(1):2598935. doi: 10.1080/07853890.2025.2598935 (PMC12777875; doi:10.1080/07853890.2025.2598935)
Supplement: Supplemental Material [file IANN_A_2598935_SM0031.zip › suppl_data/Supplementary Table 7.docx]

**Supplementary Table 7. Meta-analysis of the Circulatory System-Related Toxicity of Lenvatinib**

| **Author (year)** | **Any Grade** | | | | | | | | | | **Grade ≥ 3** | | | | | | | | | |
| --- | --- | --- | --- | --- | --- | --- | --- | --- | --- | --- | --- | --- | --- | --- | --- | --- | --- | --- | --- | --- |
|  | **Vascular n/N (%)** | | **Blood system n/N (%)** | | | | | **Heart n/N (%)** | | | **Vascular n/N (%)** | | **Blood system n/N (%)** | | | | | **Heart n/N (%)** | | |
|  | **Hypertension** | **Hemorrhagic Events** | **Hypertriglyceridaemia** | **Thrombocytopenia** | **Epistaxis** | **Anaemia** | **Hemorrhage** | **Cardiac Failure** | **Arterial Thromboembolic Event** | **QT Prolongation** | **Hypertension** | **Hemorrhagic Events** | **Hypertriglyceridaemia** | **Thrombocytopenia** | **Epistaxis** | **Anaemia** | **Hemorrhage** | **Cardiac Failure** | **Arterial Thromboembolic Event** | **QT Prolongation** |
| Casadei-Gardini et al. (2023) | 422/1343 (31.5%)vs 223/864 (25.8%) | NR | NR | NR | NR | NR | NR | NR | NR | NR | 80/1343 (6.0%)vs 57/864 (6.6%) | NR | NR | NR | NR | NR | NR | NR | NR | NR |
| Haddad et al. (2017) | NR | NR | NR | NR | NR | NR | NR | NR | NR | NR | NR | NR | NR | NR | NR | NR | NR | NR | NR | NR |
| Kiyota et al. (2017) | 254/379 (67.0%) vs 20/204 (9.8%) | NR | NR | NR | NR | NR | NR | NR | NR | NR | 153/379 (40.4%) vs 5/204 (2.45%) | NR | NR | NR | NR | NR | NR | NR | NR | NR |
| Kudo et al. (2018) | 201/476 (42.2%) vs 144/475 (30.3%) | NR | NR | 87/476 (18.3%) vs 58/475 (12.2%) | NR | NR | NR | NR | NR | NR | 111/476 (23.3%) vs 68/475 (14.3%) | NR | NR | 26/476 (5.5%) vs 16/475 (3.4%) | NR | NR | NR | NR | NR | NR |
| Matsubara et al. (2024) | 84/241 (34.9%) vs 17/242 (7.0%) | NR | NR | NR | NR | NR | NR | 3/241 (1.2%) vs 0/242 (0%) | NR | NR | 44/241 (18.3%) vs 5/242 (2.1%) | NR | NR | NR | NR | NR | NR | 3/241 (1.2%) vs 0/242 (0%) | NR | NR |
| Motzer et al. (2015) | 25/52 (48.1%) vs 5/50 (10.0%) | NR | 7/52 (13.5%) vs 12/50 (24.0%) | NR | 4/52 (7.7%)vs 11/50 (22%) | 4/52 (7.7%) vs 13/50 (26%) | NR | NR | NR | NR | 9/52 (17.3%) vs 1/50 (2.0%) | NR | 2/52 (3.8%) vs 1/50 (2.0%) | NR | 0/52 (0%)vs 0/50 (0%) | 1/52 (1.9%) vs 6/50 (12.0%) | NR | NR | NR | NR |
| Nair et al. (2021) | 216/476 (45%) vs 149/475 (31%) | 111/476 (23%) vs 73/475 (15%) | NR | NR | NR | NR | NR | NR | NR | NR | 116/476 (24%) vs 73/475 (15%) | 21/476 (4%) vs 21/475 (4%) | NR | NR | NR | NR | NR | NR | NR | NR |
| Yang et al. (2024) | 126/309 (40.8%) vs 46/312 (14.7%) | NR | NR | NR | NR | NR | 77/309 (24.9%) vs 53/312 (17.0%) | 14/309 (4.5%) vs 7/312 (2.2%) | 11/309 (3.6%) vs 11/312 (3.5%) | 6/309 (1.9%) vs 1/312 (0.3%) | 59/309 (19.1%) vs 12/312 (3.8%) | NR | NR | NR | NR | NR | 19/309 (6.1%) vs 5/312 (1.6%) | 3/309 (1.0%) vs 2/312 (0.6%) | 6/309 (1.9%) vs 5/312 (1.6%) | 1/309 (0.3%) vs 1/312 (0.3%) |
| Zheng et al. (2021) | 84/103 (81.6%) vs 10/48 (20.8%) | NR | NR | 27/103 (26.2%) vs 0/48 (0%) | NR | NR | NR | NR | NR | NR | 64/103 (62.1%) vs 4/48 (8.3%) | NR | NR | 7/103 (6.8%) vs 0/48 (0%) | NR | NR | NR | NR | NR | NR |

NR: Not Reported.
