# Supplementary material for: Systematic meta-analysis of the toxicities and side effects of the targeted drug lenvatinib
Source: Ann Med. 2025 Dec 24;58(1):2598935. doi: 10.1080/07853890.2025.2598935 (PMC12777875; doi:10.1080/07853890.2025.2598935)
Supplement: Supplemental Material [file IANN_A_2598935_SM0031.zip › suppl_data/Supplementary Table 8.docx]

**Supplementary Table 8. Meta-analysis of the Toxicity of Lenvatinib on the Skin and Sppendages**

| **Author (year)** | **Any Grade** | | | | | | | | | | | **Grade ≥ 3** | | | | | | | | | | |
| --- | --- | --- | --- | --- | --- | --- | --- | --- | --- | --- | --- | --- | --- | --- | --- | --- | --- | --- | --- | --- | --- | --- |
|  | **Skin/Subcutaneous Tissue n/N (%)** | | | | | **Taste System n/N (%)** | | | | | | **Skin/Subcutaneous Tissue n/N (%)** | | | | | **Taste System n/N (%)** | | | | | |
|  | **Palmar-Plantar Erythrodysesthesia Syndrome** | **Rash** | **Alopecia** | **Pruritus** | **Dry Skin** | **Dysgeusia** | **Nasopharyngitis** | **Stomatitis** | **Oral Pain** | **Mouth Ulceration** | **Toothache** | **Palmar-Plantar Erythrodysesthesia Syndrome** | **Rash** | **Alopecia** | **Pruritus** | **Dry skin** | **Dysgeusia** | **Nasopharyngitis** | **Stomatitis** | **Oral Pain** | **Mouth Ulceration** | **Toothache** |
| Casadei-Gardini et al. (2023) | 300/1343 (22.3%) vs 21/864 (2.5%) | NR | NR | NR | NR | NR | NR | NR | NR | NR | NR | 29/1343 (2.2%) vs 5/864 (0.6%) | NR | NR | NR | NR | NR | NR | NR | NR | NR | NR |
| Haddad et al. (2017) | 90/261 (34.5%) vs 0/131 (0%) | 26/262 (10.0%)vs 2/131 (1.5%) | NR | NR | NR | NR | NR | NR | NR | NR | NR | NR | NR | NR | NR | NR | NR | NR | NR | NR | NR | NR |
| Kiyota et al. (2017) | 112/379 (29.6%) vs 2/204 (1.0%) | NR | NR | NR | NR | 67/379 (17.7%) vs 3/204 (1.5%) | NR | 135/379 (35.6%) vs 9/204 (4.4%) | NR | NR | NR | 11/379 (2.9%) vs 0/204 (0%) | NR | NR | NR | NR | 0/379 (0%) vs 0/204 (0%) | NR | 19/379 (5.0%) vs 0/204 (0%) | NR | NR | NR |
| Kudo et al. (2018) | 128/476 (26.9%) vs 249/475 (52.4%) | 46/476 (9.7%) vs 76/475 (16.0%) | 14/476 (2.9%) vs 119/475 (25.1%) | NR | NR | NR | NR | NR | NR | NR | NR | 14/476 (2.9%) vs 54/475 (11.4%) | 0/476 (0%) vs 2/475 (0.4%) | 0/476 (0%) vs 0/475 (0%) | NR | NR | NR | NR | NR | NR | NR | NR |
| Matsubara et al. (2024) | NR | 24/241 (10.0%) vs 14/242 (5.8%) | NR | 27/241 (11.2%) vs 35/242 (14.5%) | NR | NR | NR | NR | NR | NR | NR | NR | 4/241 (1.7%) vs 1/242 (0.4%) | NR | 0/241 (0%) vs 0/242 (0%) | NR | NR | NR | NR | NR | NR | NR |
| Motzer et al. (2015) | 8/52 (15.4%) vs 2/50 (4.0%) | 9/52 (17.3%) vs 11/50 (22.0%) | NR | 3/52 (5.8%) vs 7/50 (14.0%) | 3/52 (5.8%) vs 3/50 (6.0%) | NR | 3/52 (5.8%) vs 6/50 (12%) | 13/52 (25%) vs 21/50 (42.0%) | 5/52 (9.6%) vs 1/50 (2.0%) | 0/52 (0%) vs 5/50 (10.0%) | 3/52 (5.8%) vs 1/50 (2.0%) | 0/52 (0%) vs 0/50 (0%) | 0/52 (0%) vs 0/50 (0%) | NR | 0/52 (0%) vs 0/50 (0%) | 0/52 (0%) vs 0/50 (0%) | NR | 0/52 (0%) vs 0/50 (0%) | 1/52 (1.9%) vs 1/50 (2.0%) | 0/52 (0%) vs 0/50 (0%) | 0/52 (0%) vs 1/50 (2.0%) | 0/52 (0%) vs 0/50 (0%) |
| Nair et al. (2021) | 130/476 (27%) vs 247/475 (52%) | 68/476 (14%) vs 114/475 (24%) | NR | NR | NR | NR | NR | 54/476 (11%) vs 68/475 (14%) | NR | NR | NR | 16/476 (3%) vs 54/475 (11%) | 0/476 (0%) vs 11/475 (2%) | NR | NR | NR | NR | NR | 2/476 (0.4%) vs 6/475 (1%) | NR | NR | NR |
| Yang et al. (2024) | 34/309 (11.0%) vs 3/312 (1.0%) | NR | NR | NR | NR | NR | NR | NR | NR | NR | NR | 6/309 (1.9%) vs 0/312 (0%) | NR | NR | NR | NR | NR | NR | NR | NR | NR | NR |
| Zheng et al. (2021) | 60/103 (58.3%) vs 2/48 (4.2%) | NR | NR | NR | NR | NR | NR | NR | NR | NR | NR | 10/103 (9.7%) vs 0/48 (0%) | NR | NR | NR | NR | NR | NR | NR | NR | NR | NR |

NR: Not Reported.
