# Supplementary material for: Systematic meta-analysis of the toxicities and side effects of the targeted drug lenvatinib
Source: Ann Med. 2025 Dec 24;58(1):2598935. doi: 10.1080/07853890.2025.2598935 (PMC12777875; doi:10.1080/07853890.2025.2598935)
Supplement: Supplemental Material [file IANN_A_2598935_SM0031.zip › suppl_data/Supplementary Table 9.docx]

**Supplementary Table 9. Meta-analysis of the Respiratory System Toxicity of Lenvatinib**

| **Author (year)** | **Any Grade** | | | | | | **Grade ≥ 3** | | | | | |
| --- | --- | --- | --- | --- | --- | --- | --- | --- | --- | --- | --- | --- |
|  | **Respiratory, Thoracic, and Mediastinal n/N (%)** | | | **Respiratory Tract n/N (%)** | | | **Respiratory, Thoracic and Mediastinal n/N (%)** | | | **Respiratory Tract n/N (%)** | | |
|  | **Dyspnoea** | **Exertional Dyspnoea** | **Dysphonia** | **Pneumonitis** | **Upper-Respiratory-Tract Infection** | **Lower-Respiratory-Tract Infection** | **Dyspnoea** | **Exertional Dyspnoea** | **Dysphonia** | **Pneumonitis** | **Upper-respiratory-tract infection** | **Lower-respiratory-tract infection** |
| Casadei-Gardini et al. (2023) | NR | NR | NR | NR | NR | NR | NR | NR | NR | NR | NR | NR |
| Haddad et al. (2017) | NR | NR | NR | NR | NR | NR | NR | NR | NR | NR | NR | NR |
| Kiyota et al. (2017) | NR | NR | 95/379 (25.1%) vs 7/204 (3.4%) | NR | NR | NR | NR | NR | 4/379 (1.1%) vs 0/204 (0%) | NR | NR | NR |
| Kudo et al. (2018) | NR | NR | 113/476 (23.8%) vs 57/475 (12.0%) | NR | NR | NR | NR | NR | 1/476 (0.2%) vs 0/475 (0%) | NR | NR | NR |
| Matsubara et al. (2024) | NR | NR | 26/241 (10.8%) vs 1/242 (0.4%) | 8/241 (3.3%) vs 6/242 (2.5%) | NR | NR | NR | NR | 0/241 (0%) vs 0/242 (0%) | 3/241 (1.2%) vs 2/242 (0.8%) | NR | NR |
| Motzer et al. (2015) | 11/52 (21.2%) vs 11/50 (22.0%) | 1/52 (1.9%) vs 5/50 (10.0%) | 19/52 (36.5%) vs 2/50 (4.0%) | NR | 7/52 (13.5%) vs 3/50 (6.0%) | 4/52 (7.7%) vs 6/50 (12.0%) | 1/52 (1.9%) vs 4/50 (8.0%) | 0/52 (0%) vs 0/50 (0%) | 0/52 (0%) vs 0/50 (0%) | NR | 0/52 (0%) vs 0/50 (0%) | 4/52 (7.7%) vs 1/50 (2.0%) |
| Nair et al. (2021) | NR | NR | 116/476 (24%) vs 57/475 (12%) | NR | NR | NR | NR | NR | 1/476 (0.2%) vs 0/475 (0%) | NR | NR | NR |
| Yang et al. (2024) | NR | NR | NR | NR | NR | NR | NR | NR | NR | NR | NR | NR |
| Zheng et al. (2021) | NR | NR | NR | NR | NR | NR | NR | NR | NR | NR | NR | NR |

NR: Not Reported.
