# Supplementary figures and images for: Systematic meta-analysis of the toxicities and side effects of the targeted drug lenvatinib
Source: Ann Med. 2025 Dec 24;58(1):2598935. doi: 10.1080/07853890.2025.2598935 (PMC12777875; doi:10.1080/07853890.2025.2598935)

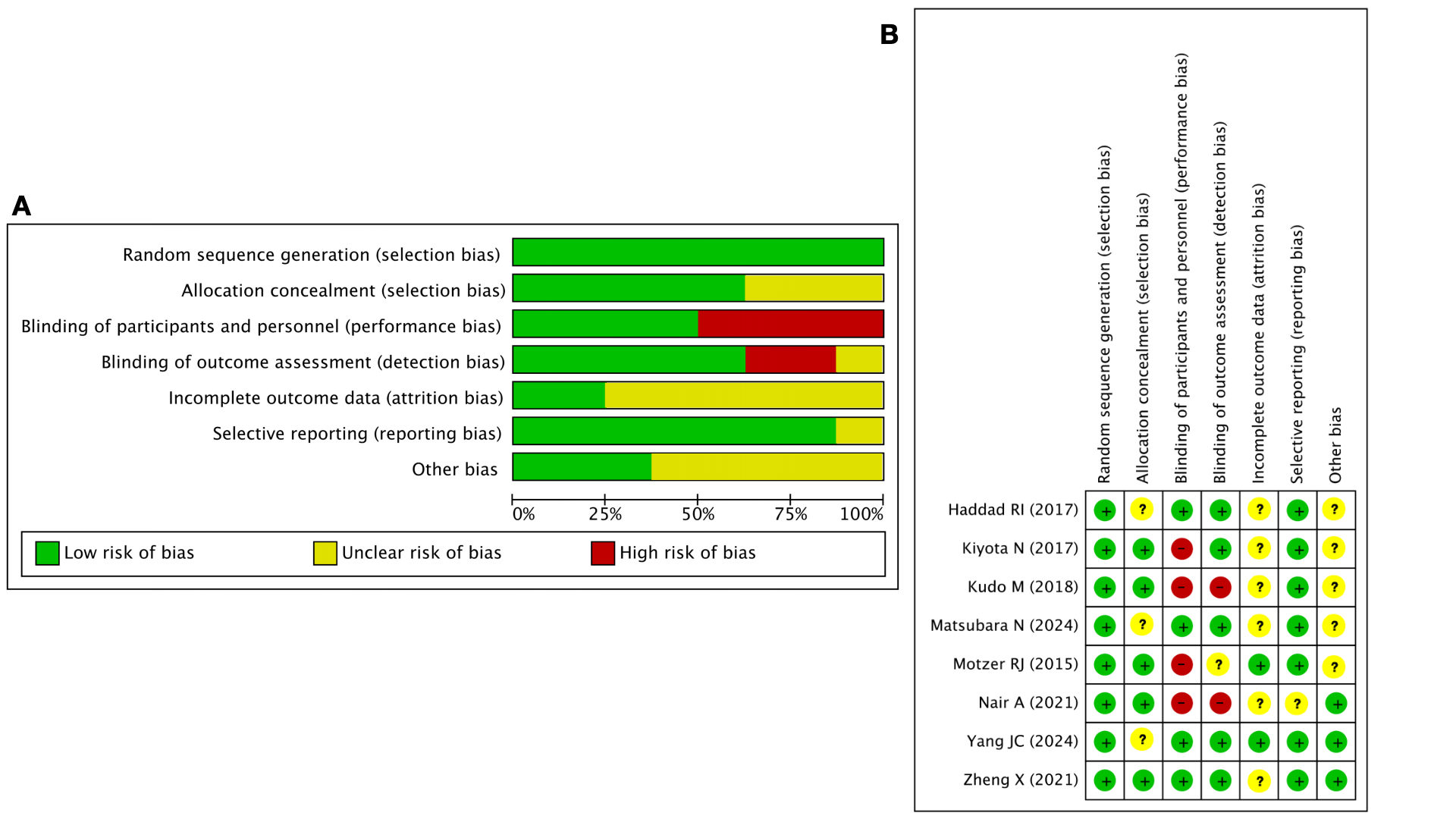

Supplement: Supplemental Material [file IANN_A_2598935_SM0031.zip › suppl_data/Supplementary Figure 1.tif]

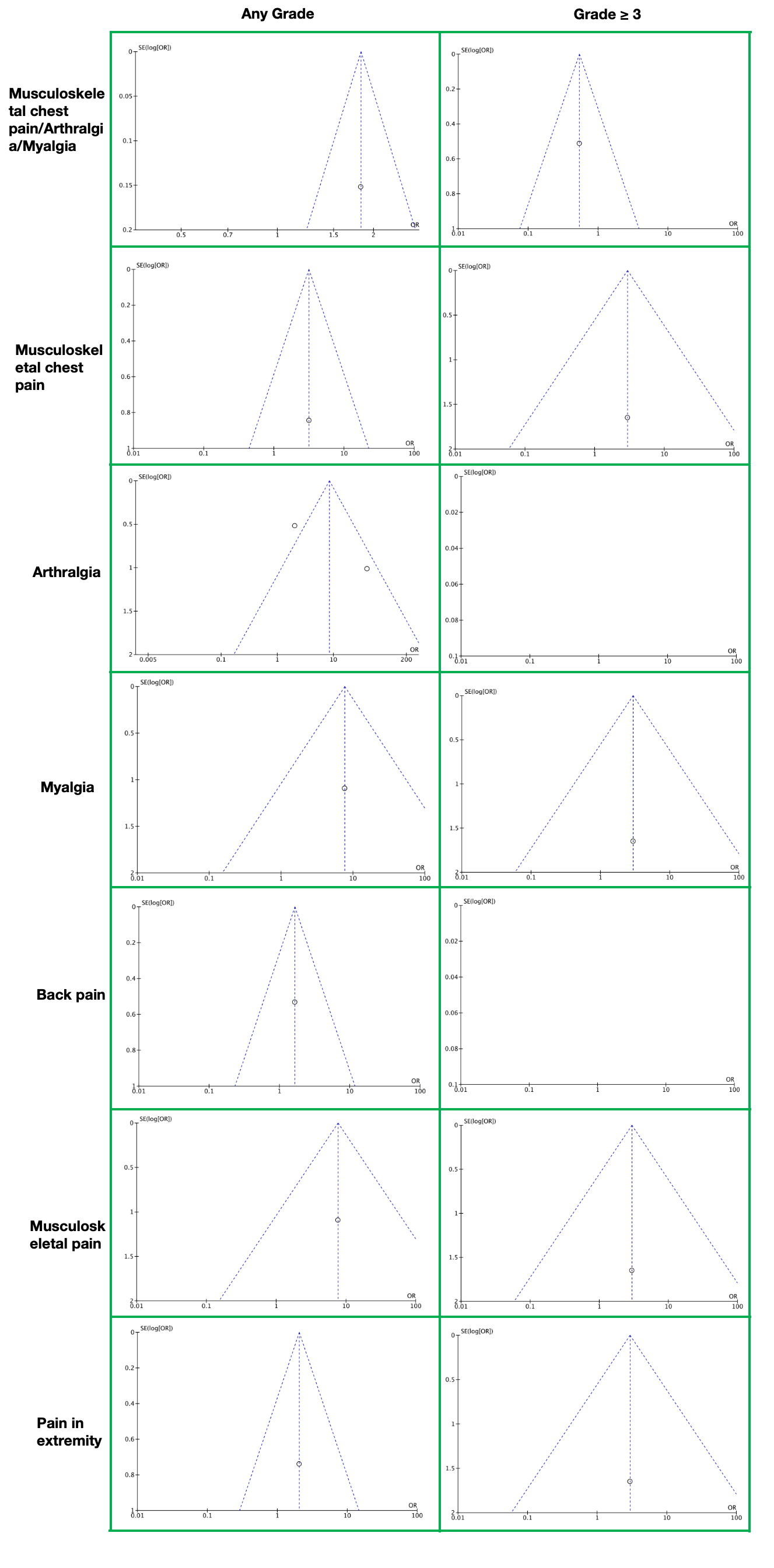

Supplement: Supplemental Material [file IANN_A_2598935_SM0031.zip › suppl_data/Supplementary Figure 10.tif]

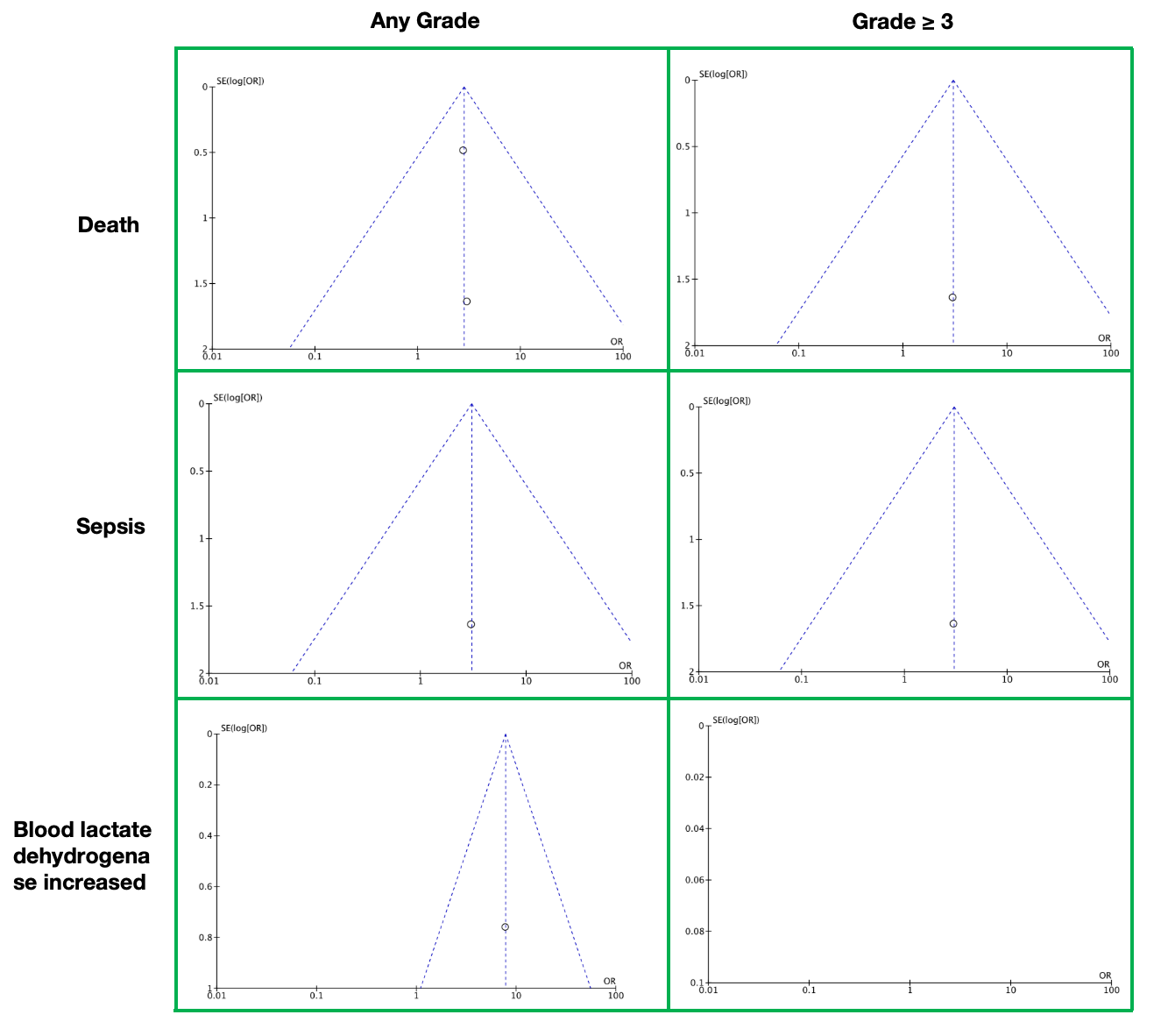

Supplement: Supplemental Material [file IANN_A_2598935_SM0031.zip › suppl_data/Supplementary Figure 11.tif]

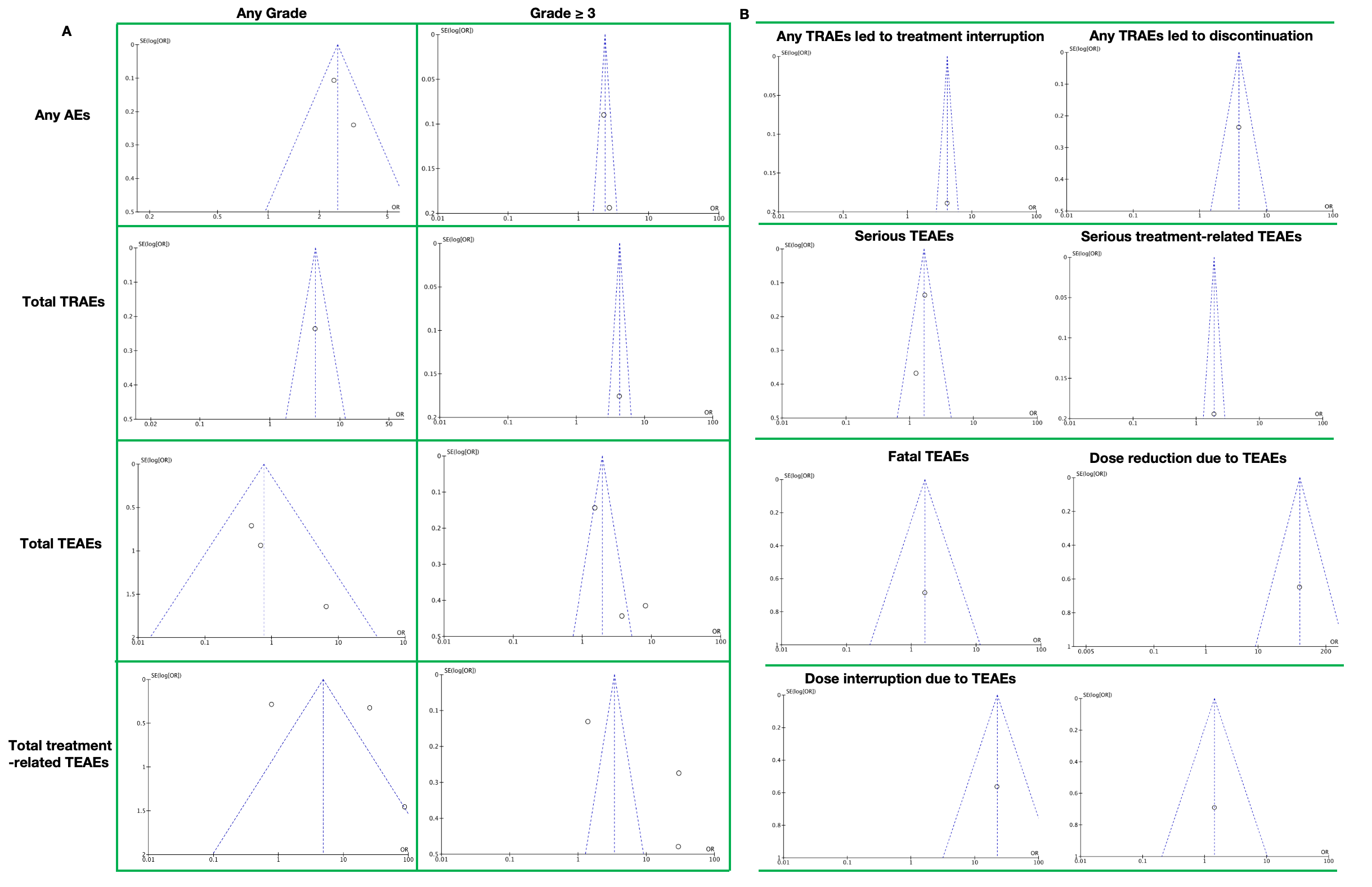

Supplement: Supplemental Material [file IANN_A_2598935_SM0031.zip › suppl_data/Supplementary Figure 2.tif]

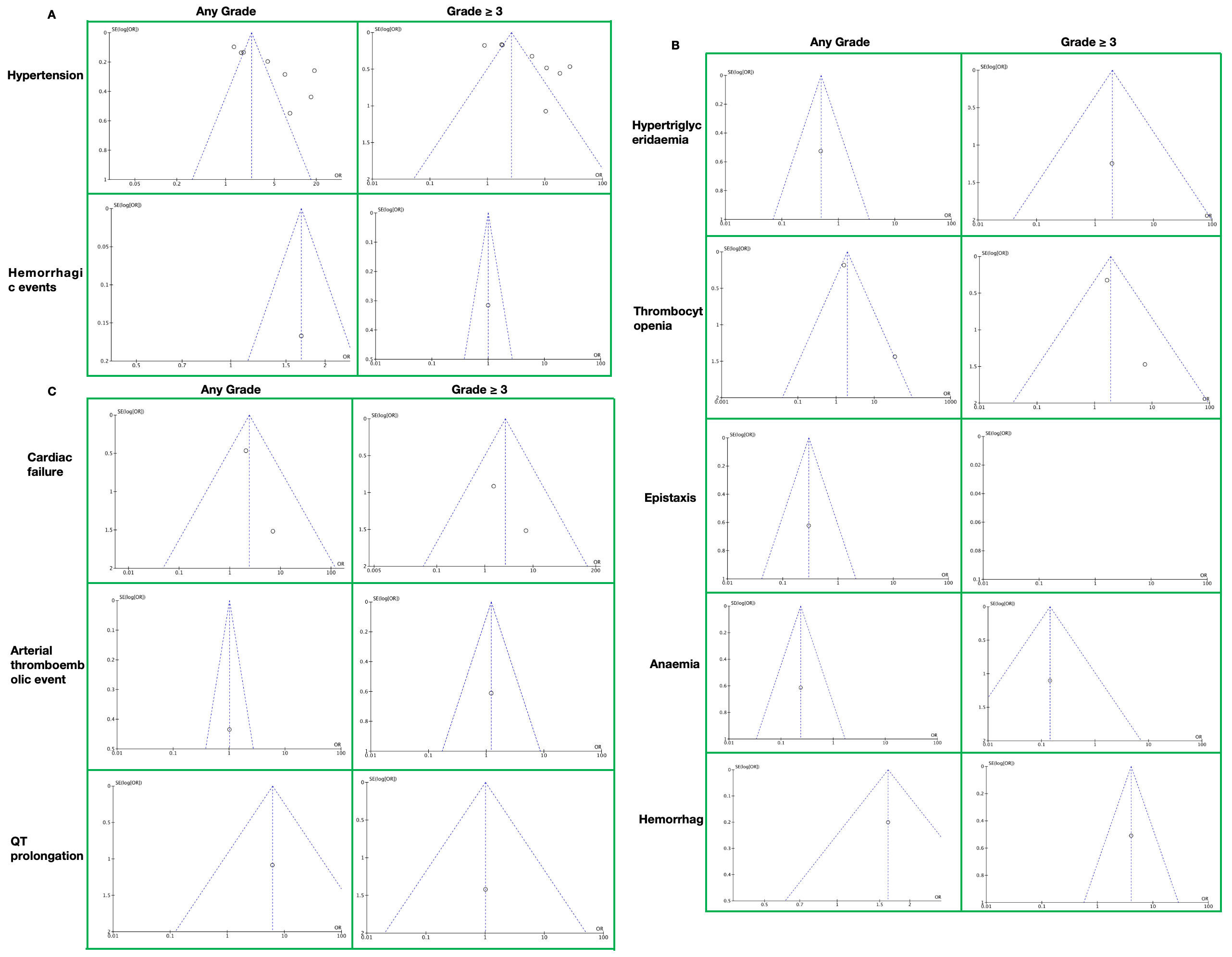

Supplement: Supplemental Material [file IANN_A_2598935_SM0031.zip › suppl_data/Supplementary Figure 3.tif]

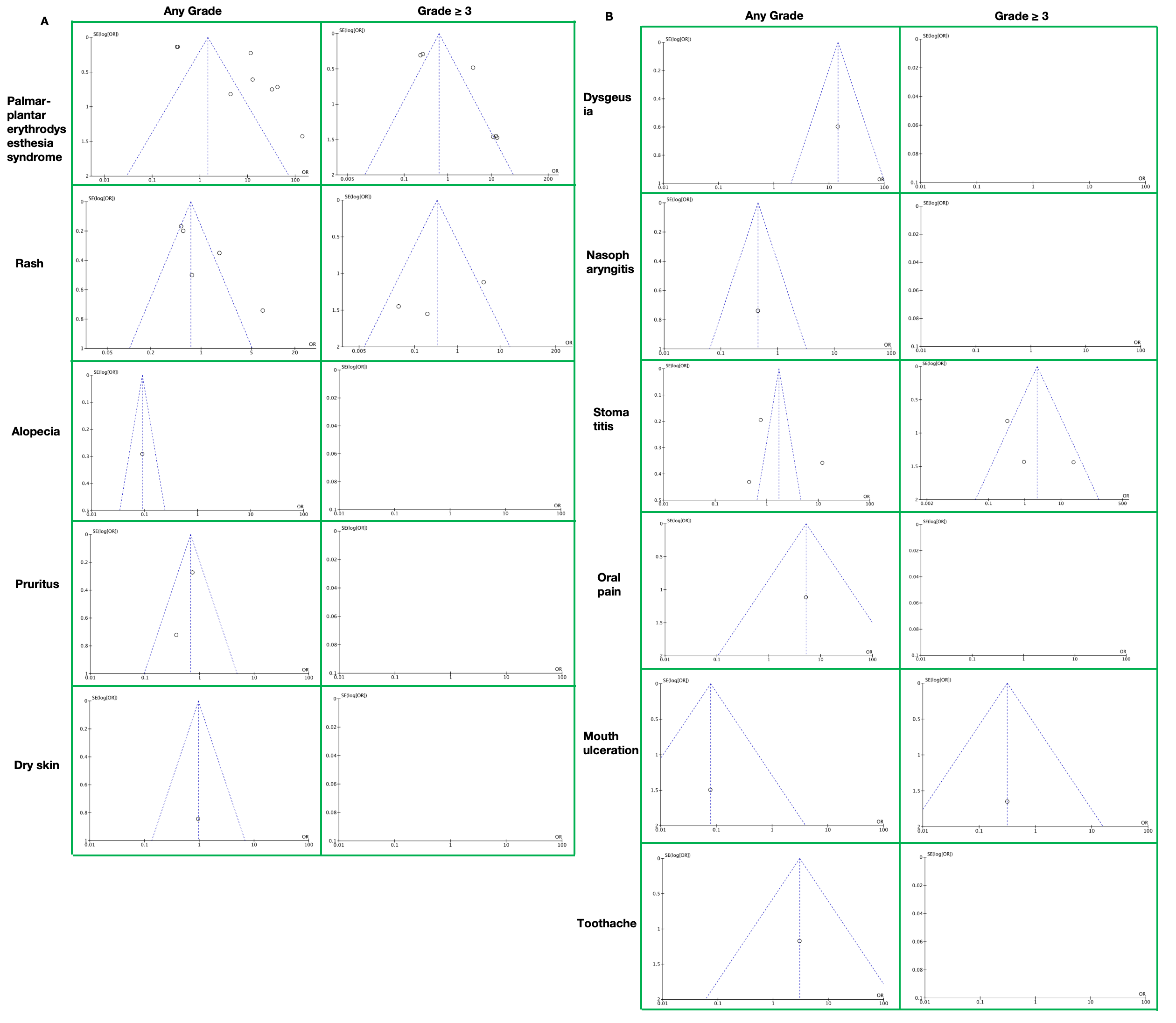

Supplement: Supplemental Material [file IANN_A_2598935_SM0031.zip › suppl_data/Supplementary Figure 4.tif]

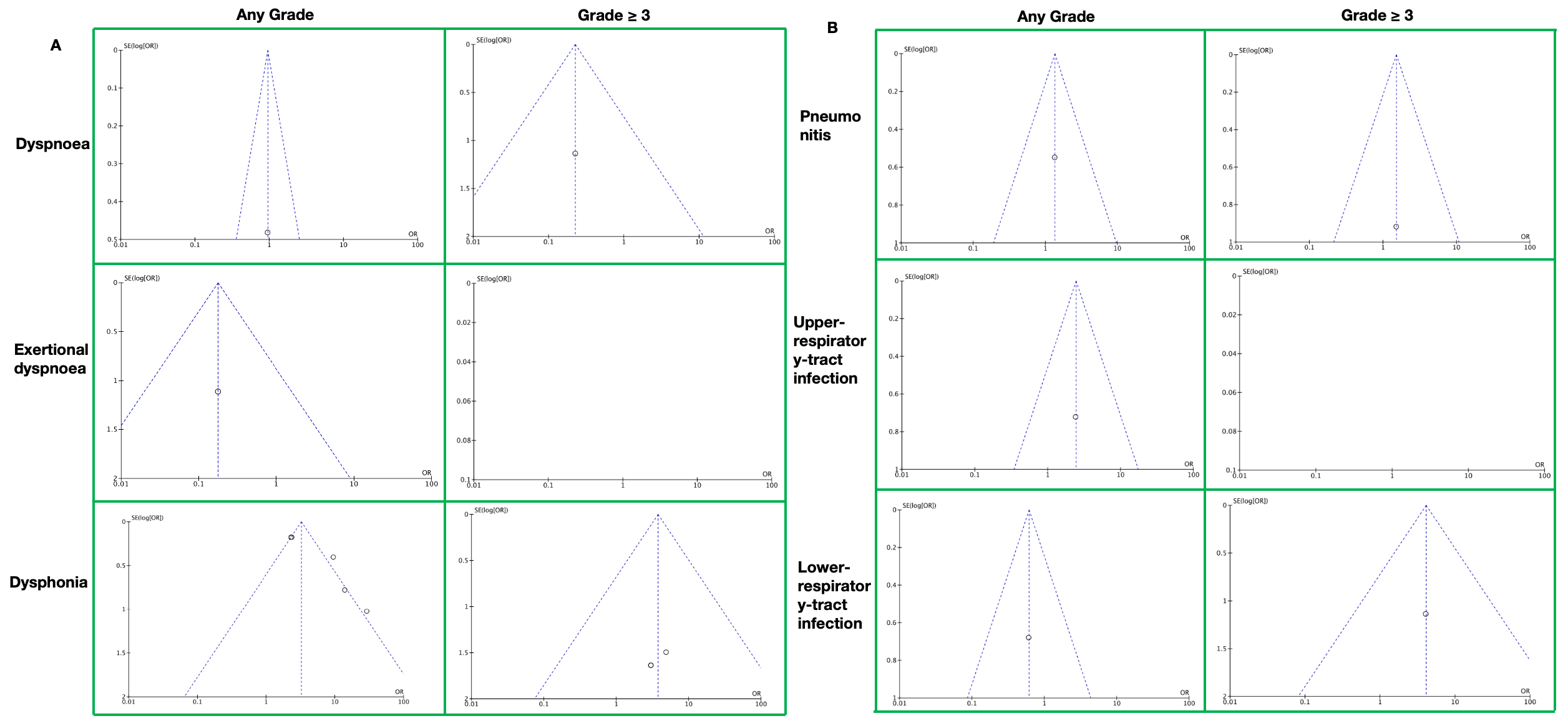

Supplement: Supplemental Material [file IANN_A_2598935_SM0031.zip › suppl_data/Supplementary Figure 5.tif]

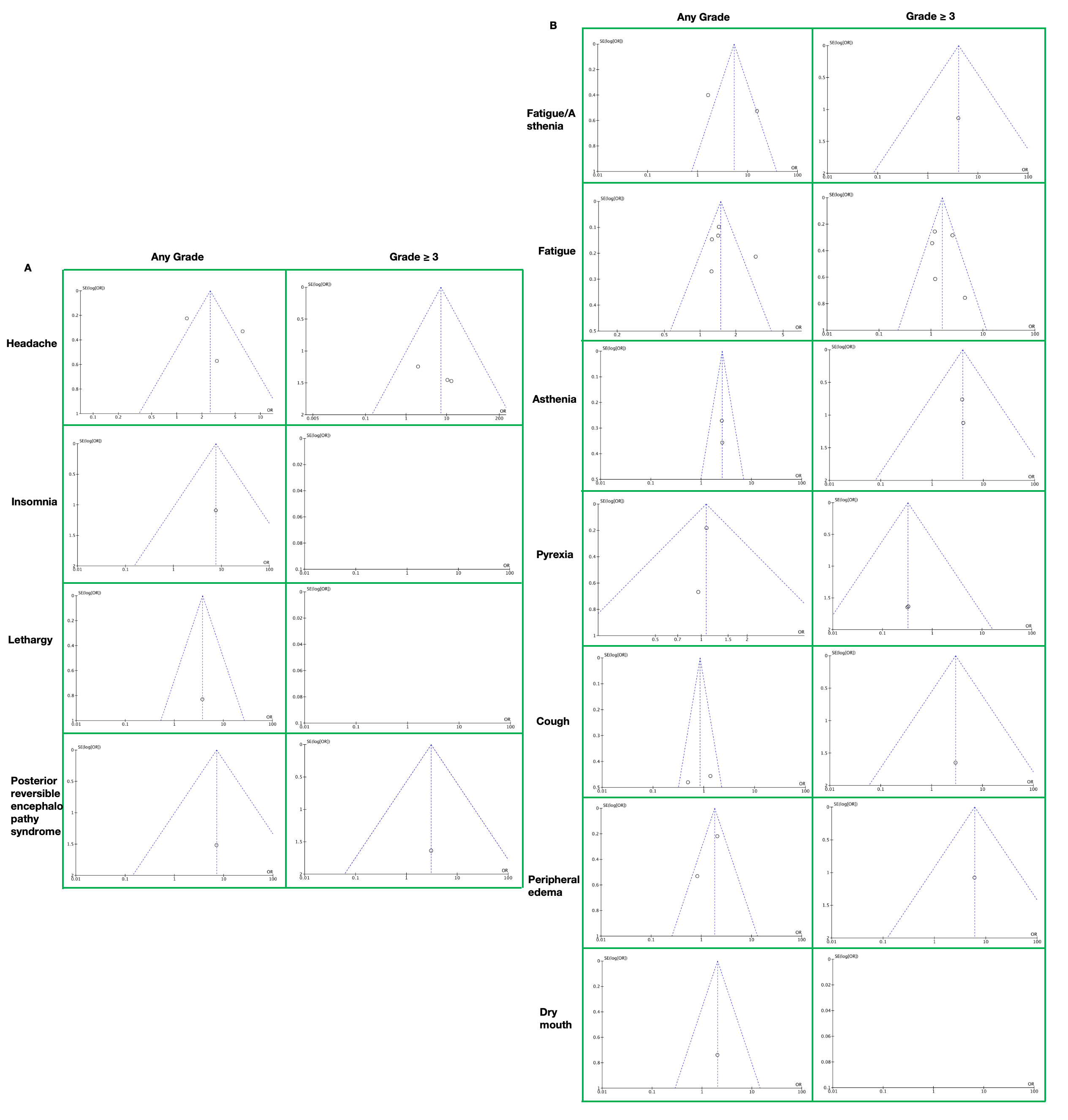

Supplement: Supplemental Material [file IANN_A_2598935_SM0031.zip › suppl_data/Supplementary Figure 6.tif]

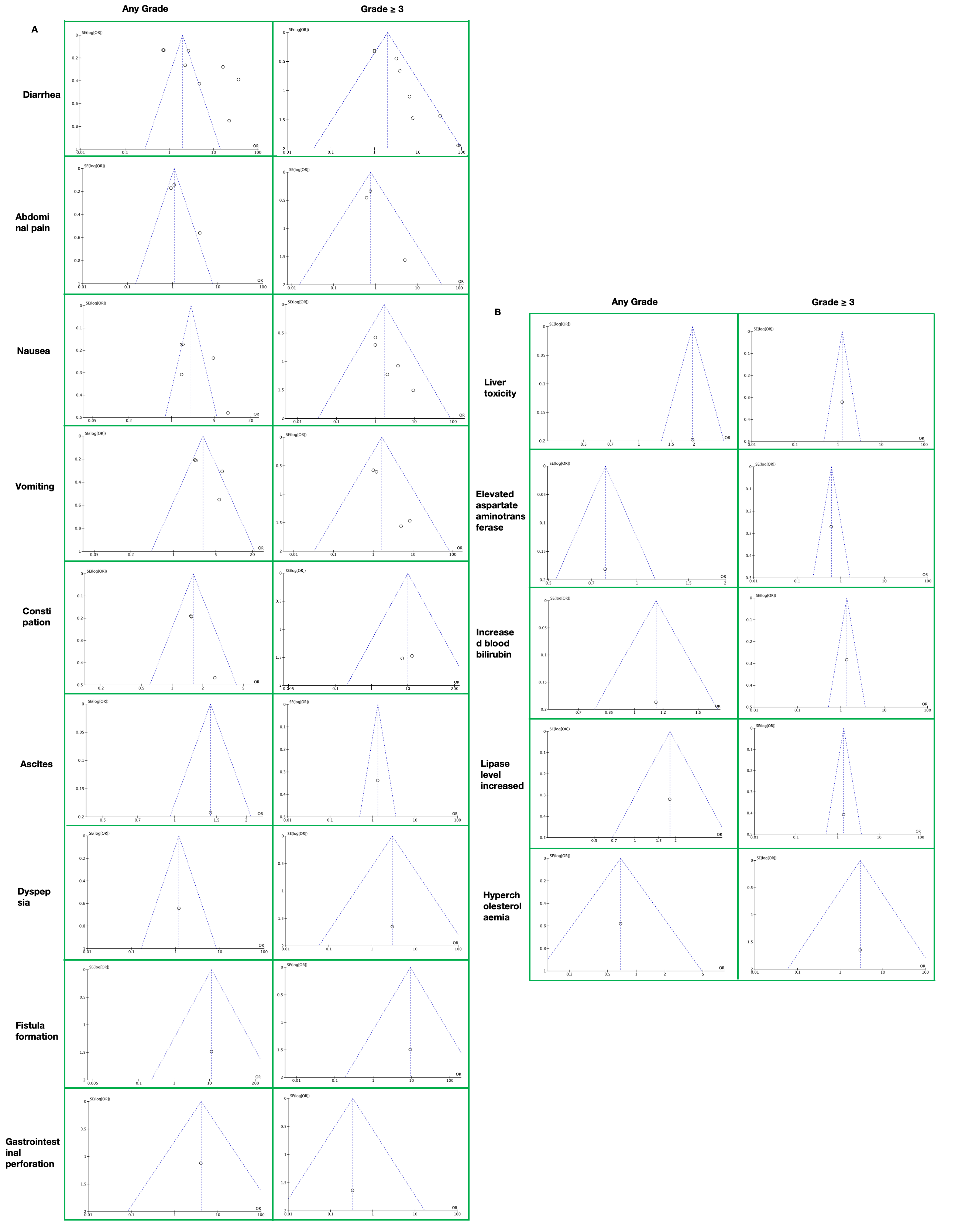

Supplement: Supplemental Material [file IANN_A_2598935_SM0031.zip › suppl_data/Supplementary Figure 7.tif]

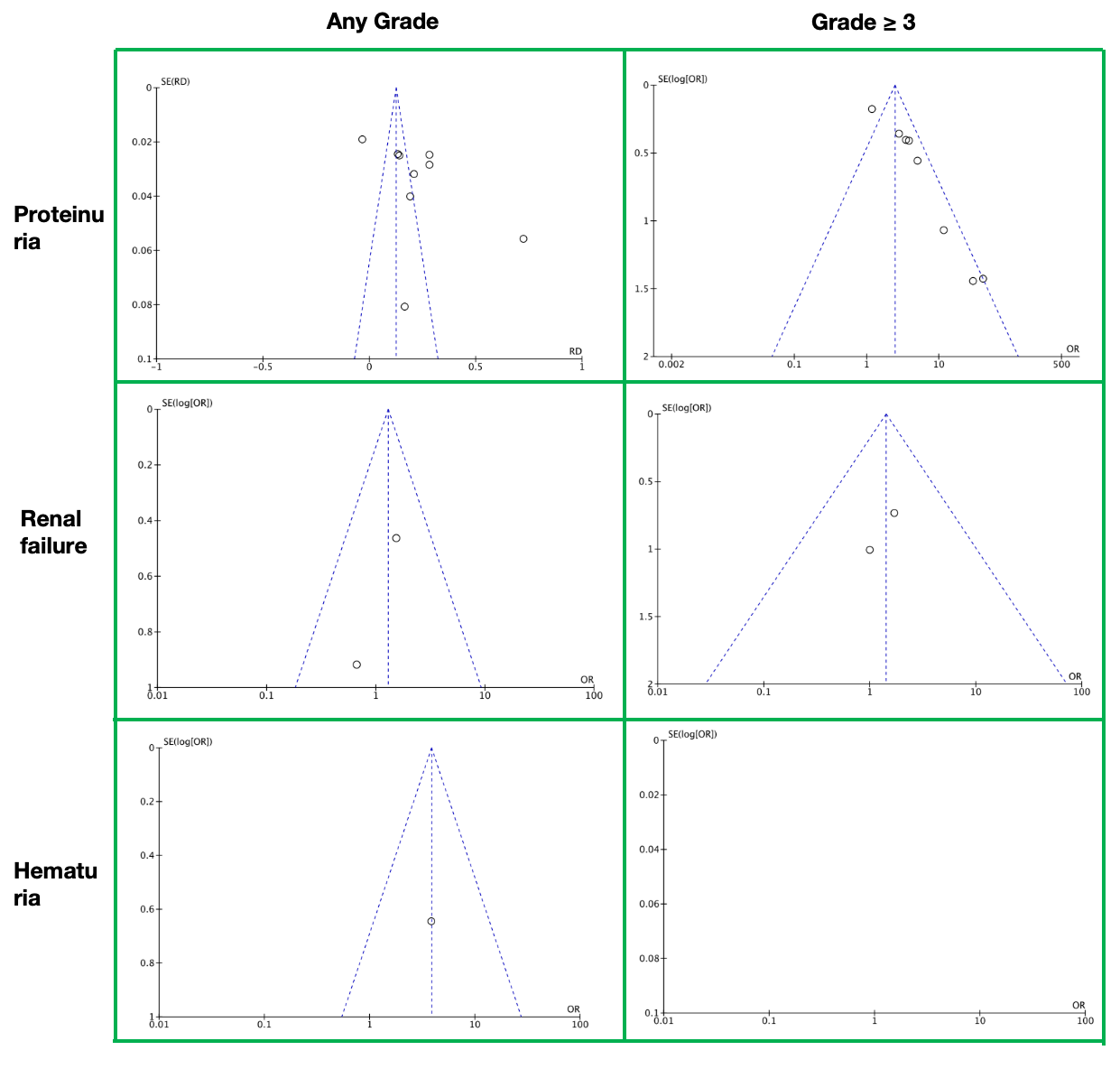

Supplement: Supplemental Material [file IANN_A_2598935_SM0031.zip › suppl_data/Supplementary Figure 8.tif]

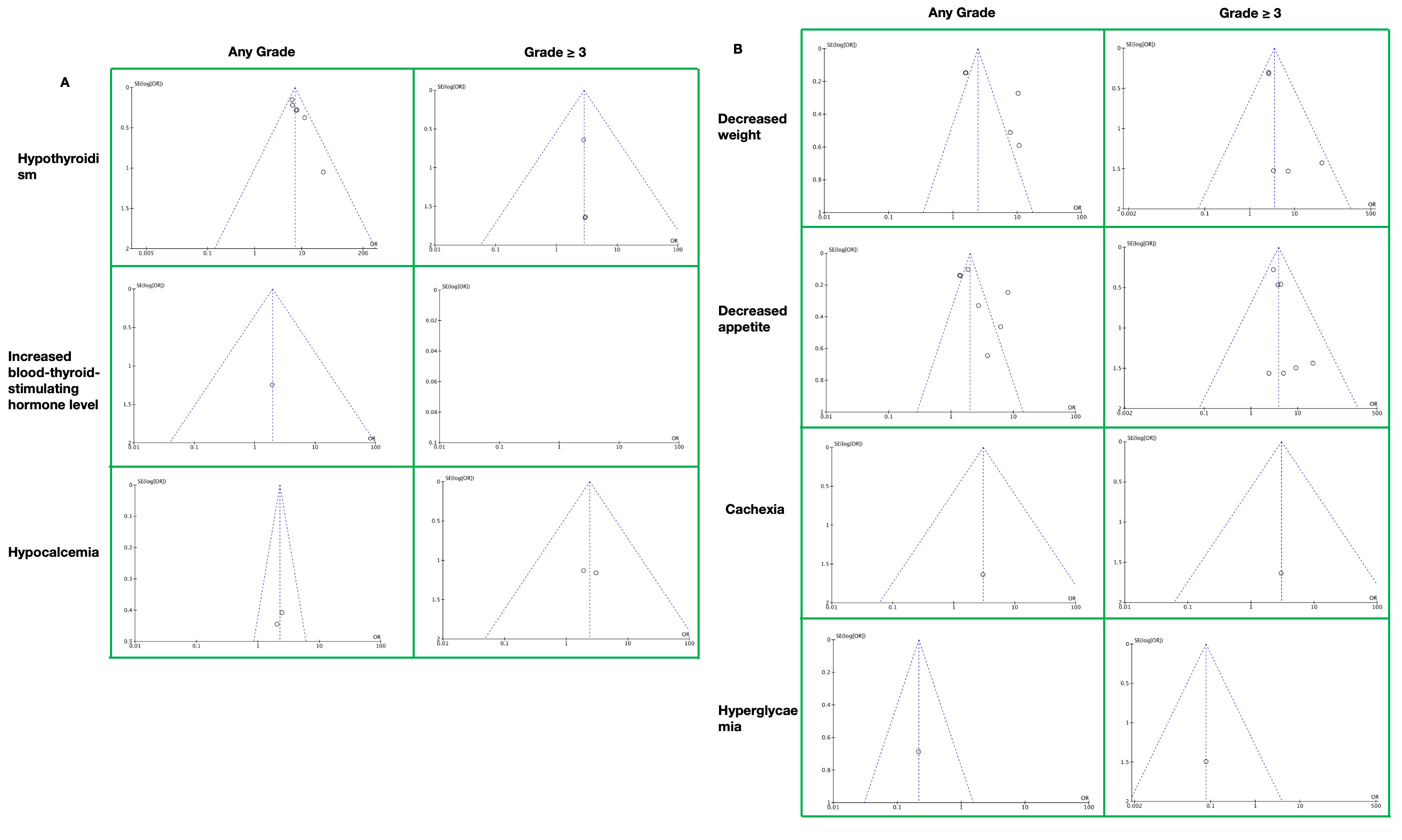

Supplement: Supplemental Material [file IANN_A_2598935_SM0031.zip › suppl_data/Supplementary Figure 9.tif]
